# Rapid transformation of sulfinate salts into sulfonates promoted by a hypervalent iodine(III) reagent

**DOI:** 10.3762/bjoc.14.101

**Published:** 2018-05-24

**Authors:** Elsa Deruer, Vincent Hamel, Samuel Blais, Sylvain Canesi

**Affiliations:** 1 Laboratoire de Méthodologie et Synthèse de Produits Naturels, Université du Québec à Montréal, C.P. 8888, Succ. Centre-Ville, Montréal, H3C 3P8 Québec, Canada

**Keywords:** hypervalent iodine, oxidation, sulfinates, sulfonation, sulfonium

## Abstract

An alternative method for forming sulfonates through hypervalent iodine(III) reagent-mediated oxidation of sodium sulfinates has been developed. This transformation involves trapping reactive sulfonium species using alcohols. With additional optimization of the reaction conditions, the method appears extendable to other nucleophiles such as electron-rich aromatic systems or cyclic ethers through a ring opening pathway.

## Introduction

Over the past few decades, hypervalent iodine reagents [1–4] have emerged as versatile and environmentally benign substitutes for heavy metal reagents. A number of iodanes with various oxidation states have been developed since the pioneering work of the German chemist Willgerodt, who synthesized PhICl_2_ [5]. Iodane reagents have been extensively used in applications such as oxidation, rearrangement, cross-coupling, functionalization, decarboxylation, and fragmentation [6–27]. The sulfonate group is a useful functionality frequently employed as a leaving group in substitution reactions. Production of sulfonates [28] from alcohols generally involves reaction with a sulfonyl chloride in the presence of a base to trap the hydrochloric acid byproduct. As an alternative method involving oxidation rather than chloride substitution, we envisaged generating an electrophilic sulfonium species through oxidation of a sulfinate salt [29] that would be subsequently trapped by the alcohol. In this paper, we demonstrate that sulfonates may be produced from alcohols in the presence of sufinates through a reaction mediated by a hypervalent iodine reagent. Under these conditions, the byproduct is a weak acid such as acetic acid rather than hydrochloric acid.

## Results and Discussion

**Scheme 1:**
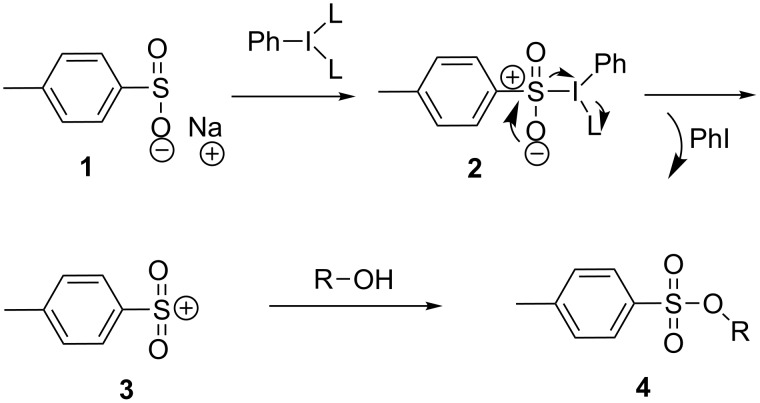
Mechanistic hypothesis.

Oxidative sulfonate production methods employing strong oxidizing agents such as chlorine have been previously reported [30]. More recently, a mild and efficient method enabling the production of aromatic sulfonates using phenols and iodine was developed [31–32]. This method uses methanol as a solvent and appears to be selective for phenols; only two primary alcohol examples were produced in 63–67% yield in the presence of a strong base. A radical pathway from the alkoxide species was proposed by the authors as an explanation for the phenol selectivity under weakly basic conditions in the presence of methanol. As a complement to this interesting method, we propose extending the process to aliphatic alcohols through activation by an iodane, acting through an alternative pathway involving a sulfonium species derived from a sulfinate **1**. It should be noted that our method would not be compatible in presence of phenols. We hypothesized that the mechanism would initially involve iodane activation of the sulfur lone pair leading to **2**. Elimination of the iodane would subsequently produce the sulfonium ion **3**, which could be trapped by an alcohol nucleophile leading to sulfonate **4** (Scheme 1).

To verify our hypothesis tosyl-sulfinate **1** was treated with iodanes such as sodium periodate (NaIO_4_), Dess-Martin periodinane (DMP) [33], 2-iodoxybenzoic acid (IBX) [34], (diacetoxyiodo)benzene (DIB), phenyliodine(III) bis(trifluoroacetate) (PIFA) in the presence of methanol. (III)-Iodanes and (V)-iodanes were both acceptable substrates, but the process was inefficient with (VII)-iodane species. We surmise that IBX and DMP are rapidly reduced to a (III)-iodane in the presence of an alcohol, and that this species is most likely the reagent promoting the formation of compound **4a**. Iodine and *N*-iodosuccinimide (NIS) were also tested; it appeared that this process was much more efficient in the presence of iodane sources (Table 1).

**Table 1:** Oxidative sulfonylation process mediated by iodine and iodine derivatives.

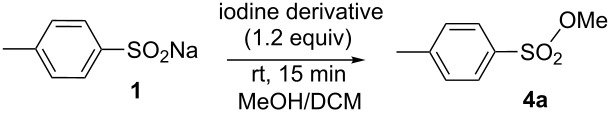		

entry	iodane	yield (%)

a	NaIO_4_	–
b	IBX	89
c	DMP	98
d	DIB	98
e	PIFA	98
f	I_2_	48
g	NIS	58

DIB was chosen as the hypervalent iodine reagent of choice since it is more compatible with alcohols than IBX or DMP. The reaction proceeded in modest to good yields depending on the structure of the alcohol. We were pleased to observe successful transformations even in the presence of poorly reactive alcohols such as trifluoroethanol (TFE, Table 2, entry c) or hexafluoroisopropanol (HFIP, Table 2, entry d). Because of the mild conditions involved, this transformation tolerates spectator functionalities such as primary halides or alkynes (Table 2).

**Table 2:** Scope and limitations of the process.

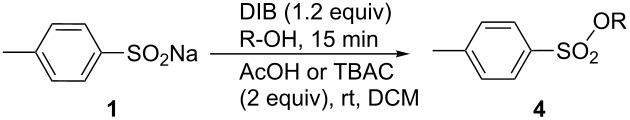		

entry	R-OH	yield (%)

a	Me-OH	99
b	Et-OH	75
c	CF_3_CH_2_-OH	95
d	(CF_3_)_2_CH-OH	48
e	(CH_3_)_2_CH-OH	74
f	ClCH_2_CH_2_-OH	51
g	BrCH_2_CH_2_CH_2_-OH	51
h	*n-*Bu-OCH_2_CH_2_-OH	57
i	HC≡CCH_2_-OH	81
j	CH_3_CHOH(CH_2_)_2_CH_3_	60
k	CH_3_CH_2_CH(CH_3_)CH_2_-OH	76
l	ClCH_2_CH(CH_3_)-OH	65
m	Ph-CH_2_-CH_2_-OH	50
n	*t-*Bu-OH	–
o	*n-*Bu-OH	70
p	cyclopentanol	63

**Scheme 2:**
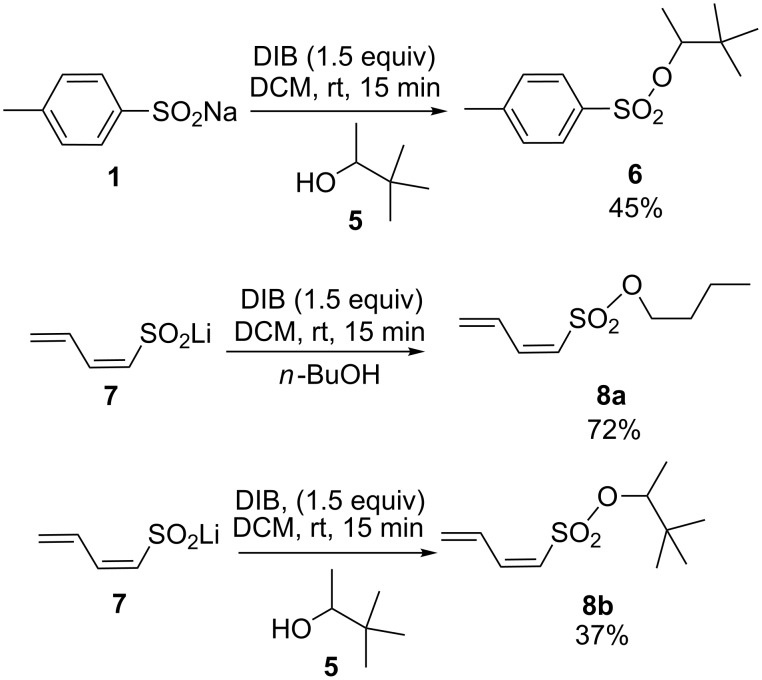
Extension of the method.

We were disappointed to observe no reaction in the presence of tertiary alcohols such as *tert*-butanol (Table 2, entry n). However, the reaction proceeded efficiently with a hindered secondary neopentylic alcohol **5** despite the presence of the neighboring *tert*-butyl group. This method could potentially be extended to other sulfinate salts, particularly aromatic or vinylic species in which the intermediate sulfonium species would be resonance stabilized. However, most commercially available sulfinates are quite expensive. It was reported in the literature that compound **7** may be easily generated from sulfolene by treatment with *n*-butyllithium [35]. This compound is further oxidized by DIB in the presence of *n*-butanol to yield sulfonate **8a** in 72% yield. The same reaction in the presence of the hindered neopentilyc alcohol **5** led to the formation of **8b** in modest yield (Scheme 2).

**Scheme 3:**
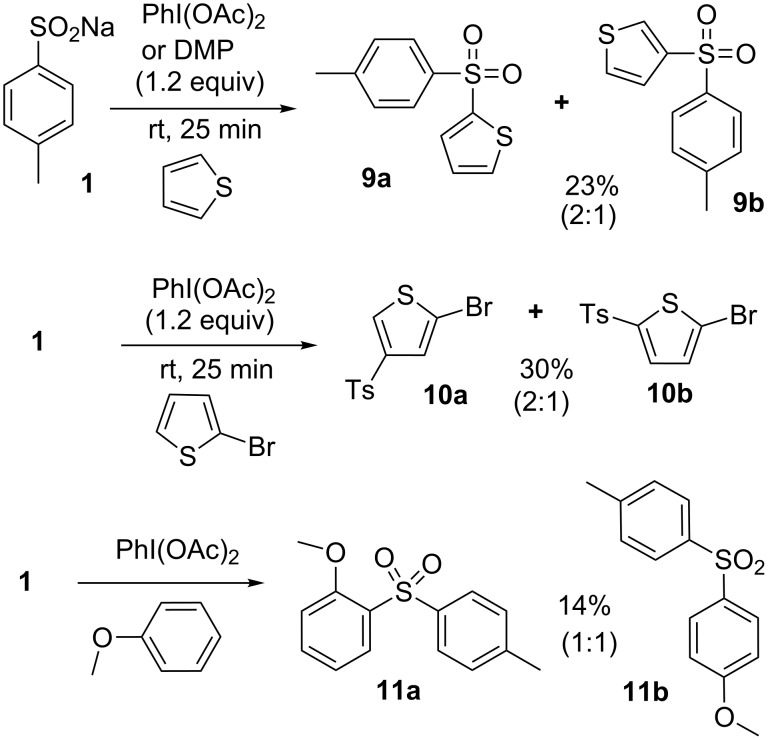
Carbon-based nucleophiles.

As a demonstration of the potential of this novel approach, we examined the possibility of involving other nucleophiles, including carbon-based nucleophiles. For instance, the alcohol in the reaction could be replaced by an electron-rich aromatic system such as thiophene or anisole. It should be stressed that the formation of substituted aromatic systems through a Friedel–Crafts type process [36] is an argument in favor of the formation of the electrophilic sulfonium species **3** (Scheme 1). In the presence of thiophene, compounds **9** were obtained in 23% yield and in a ratio (2:1) in favour of **9a**. A similar yield was observed when DMP was substituted for DIB, demonstrating that λ^5^-iodanes can also promote sulfonium activation. The reaction in the presence of 2-bromothiophene led in 30% yield to the formation of compounds **10** in a ratio (2:1) in favor of **10a**. If anisole was used instead of thiophene an expected mixture of compounds **11a** and **11b** was observed in a ratio 1:1 and in a low yield of 14%. So far, the yields observed with carbon-based nucleophiles have been low, but they clearly demonstrate the feasibility of this approach. Further investigations to extend this approach to other carbon-based nucleophiles must be developed. Presumably, the presence of an electron-donor group such as methoxy on the aromatic moiety would stabilize the sulfonium species and increase the yield obtained in these transformations (Scheme 3).

**Scheme 4:**
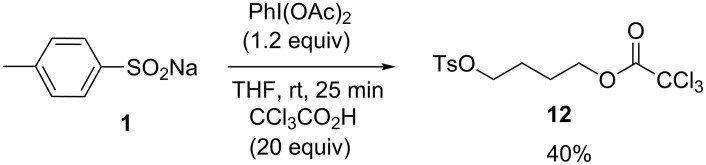
THF ring opening.

This process may also be used to open and functionalize simple heterocycles such as THF through a ring-opening approach [37]. In the presence of trichloroacetic acid and DIB, the corresponding compound **12** was obtained in 40% yield. One advantage is that this method begins with the inexpensive compound THF and produces a diol derivative containing a linear chain in only one step. One alcohol is available as a leaving group and the second is protected by conversion into a trichloroacetate moiety (Scheme 4).

## Conclusion

A novel oxidative method for producing sulfonates from sulfinates using hypervalent iodine reagents has been developed. This process involves the formation of a reactive sulfonium species that is subsequently trapped by nucleophiles. As a proof of concept, we demonstrated that the method is extendable to other nucleophiles such as electron-rich aromatics or THF. Ongoing investigations of this process and potential applications will be disclosed in due course.

## Experimental

### General procedure for the formation of sulfonate **4**

Iodobenzene diacetate (DIB, 0.24 mmol, 1.2 equiv) was added at room temperature to a vigorously stirred solution of dichloromethane (0.5 mL), alcohol (0.5 mL), sulfinate (0.2 mmol, 1 equiv) and acetic acid (0.01 to 0.05 mL) or TBAC (55.5 mg, 0.2 mmol, 2 equiv) to dissolve the sulfonate salt. The mixture was then stirred for 15 min and filtered on silica with ethyl acetate. The residue was purified using silica gel chromatography to yield sulfonate product **4**.

## Supporting Information

File 1 General procedures, synthesis of the products, spectroscopic data, and copies of ^1^H, ^13^C, NMR spectra.
