# Supplementary material for: Rapid transformation of sulfinate salts into sulfonates promoted by a hypervalent iodine(III) reagent
Source: Beilstein J Org Chem. 2018 May 24;14:1203–7. doi: 10.3762/bjoc.14.101 (PMC6009181; doi:10.3762/bjoc.14.101)
Supplement: File 1 — General procedures, synthesis of the products, spectroscopic data, and copies of 1H, 13C, NMR spectra. [file Beilstein_J_Org_Chem-14-1203-s001.pdf]

Supporting Information

for

Rapid transformation of sulfinate salts into sulfonates promoted by a hypervalent iodine(III) reagent

Elsa Deruer, Vincent Hamel, Samuel Blais and Sylvain Canesi*

Address: Laboratoire de Méthodologie et Synthèse de Produits Naturels, Université du Québec à Montréal, C.P. 8888, Succ. Centre-Ville, Montréal, H3C 3P8 Québec, Canada

Email: Sylvain Canesi - canesi.sylvain@uqam.ca

*Corresponding author

General procedures, synthesis of the products, spectroscopic data, and copies of ^{1}H, ^{13}C, NMR spectra

Table of contents

I. **General information and materials S2**

II. **Experimental procedures S2–S9.**

III. **Copies of ^{1}H and ^{13}C NMR spectra for all compounds S10–S58**

I. General information and materials:

Unless otherwise indicated, ^{1}H and ^{13}C NMR spectra were recorded at 300 and 75 MHz, respectively, in $CDCl_3$ solutions. Chemical shifts are reported in ppm on the δ scale. Multiplicities are described as s (singlet), d (doublet), dd, ddd, etc. (doublet of doublets, doublet of doublets of doublets, etc.), t (triplet), q (quartet), quin (quintuplet), sext (sextuplet), sept (septuplet),m (multiplet), and further qualified as app (apparent), br (broad). Coupling constants, J, are reported in Hz.

II. Experimental procedures:

a) General procedure for the formation of sulfonate 4:

Iodobenzene diacetate (DIB, 0.24 mmol, 1.2 equiv) was added at room temperature to a vigorously stirred solution of dichloromethane (0.5 mL), alcohol (0.5 mL), sulfinate (0.2 mmol, 1 equiv) and acetic acid (0.01 to 0.05 mL) or TBAC (55.5 mg, 0.2 mmol, 2 equiv) to solve the sulfonate salt. The mixture was then stirred for 15 min (followed by TLC with a mixture of acetic acid/ethyl acetate/hexane) and then filtered on silica with ethyl acetate. The residue was purified by silica gel chromatography with a mixture of ethyl acetate/hexane to give sulfonate product **4**, **6** and **8**.

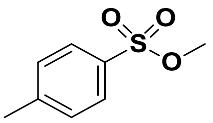

Methyl 4-methylbenzenesulfonate (4a)[1]: was prepared without dichloromethane and acetic acid, and was obtained by silica gel chromatography with a mixture of EtOAc/hexane (5/95) as a colorless oil: 0.20 mmol, 33 mg, 99% yield; ^{1}H NMR (300 MHz, $CDCl_3$) δ 7.78 (d, *J* = 8.3 Hz, 2H), 7.35 (d, *J* = 8.0 Hz, 2H), 3.73 (s, 3H), 2.44 (s, 3H), ^{13}C NMR (75 MHz, $CDCl_3$) δ 145.1, 132.3, 130.0, 128.2, 56.3, 21.7; LRMS (ESI) calcd for $C_8H_{11}O_3S$ $(M + H)^+$: 187, found 187.

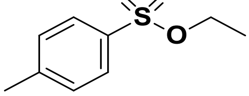

Ethyl 4-methylbenzenesulfonate (4b)[2]: was prepared without dichloromethane and acetic acid, and was obtained by silica gel chromatography with a mixture of EtOAc/hexane (5:95) as an yellow oil: 0.15 mmol, 30.5 mg, 75% yield; ^{1}H NMR (300 MHz, $CDCl_3$) δ 7.78 (d, *J* = 8.4 Hz, 2H), 7.33 (d, *J* = 8.1 Hz, 2H), 4.09 (q, *J* = 7.1 Hz, 2H),

---

[1] Chandra, J.; Chaudhuri, R.; Rao Manne, S.; Mondal, S.; Mandal, B. *ChemistrySelect* **2017**, 2, 8471

[2] Moussa, I. A.; Baniset, S. D.; Beinat, C.; Giboureau, N.; Reynolds, A. J.; Kassiou, M. *J. Med. Chem.* **2010,** 53, 6228-6239

2.43 (s, 3H), 1.28 (t, J = 7.2 Hz, 3H).^{13}C NMR (75 MHz, $CDCl_3$) δ 144.79, 133.38, 129.93, 127.94, 77.58, 77.16, 76.74, 66.92, 21.71, 14.80; LRMS (ESI) calcd for $C_9H_{13}O_3S$ $(M+H)^+$: 201; found: 201.

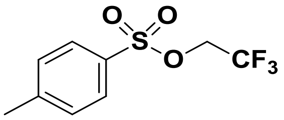

2,2,2-Trifluoroethyl 4-methylbenzenesulfonate (4c)[3]: was prepared without dichloromethane and was obtained by silica gel chromatography with a mixture of EtOAc/hexane (1:99) as a white foam: 0.190 mmol, 48.5 mg, 95% yield; ^{1}H NMR (300 MHz, $CDCl_3$) δ 7.81 (d, J = 8.3 Hz, 2H), 7.39 (d, J = 8.3 Hz, 2H), 4.35 (q, J = 7.9 Hz, 2H), 2.47 (s, 3H); ^{13}C NMR (75 MHz, $CDCl_3$) δ 146.1, 132.0, 130.3, 128.2, 122.0 (q, J = 277.7 Hz), 64.7 (q, J = 38.1 Hz), 21.8; LRMS calcd for $C_9H_9F_3O_3NaS$ $(M+Na)^+$: 277; found: 277.

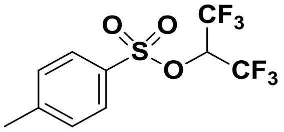

1,1,1,3,3,3-Hexafluoropropan-2-yl 4-methylbenzenesulfonate (4d)[3]: was prepared without dichloromethane and was obtained by silica gel chromatography with a mixture of EtOAc/hexane (1:99) as a white foam: 0.097 mmol, 31.2 mg, 48% yield; ^{1}H NMR (300 MHz, $CDCl_3$) δ 7.83 (d, J = 8.4 Hz, 2H), 7.40 (d, J = 8.1 Hz, 2H), 5.26 (sept, J = 5.7 Hz, 1H), 2.48 (s, 3H); ^{13}C NMR (75 MHz, $CDCl_3$) δ 146.8, 132.1, 130.3, 128.3, 120.1 (q, J = 281 Hz), 72.0 (septet, J = 35.5 Hz), 22.0; LRMS calcd for $C_{10}H_8F_6NaO_3S$ $(M+Na)^+$: 345; found: 345.

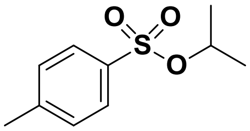

Isopropyl 4-methylbenzenesulfonate (4e)[4]: was prepared without dichloromethane and was obtained by silica gel chromatography with a mixture of EtOAc/hexane (5:95) as an yellow oil: 0.147 mmol, 31.5 mg, 74% yield; ^{1}H NMR (300 MHz, $CDCl_3$) δ 7.78 (d, J = 8.3 Hz, 2H), 7.32 (d, J = 8.0 Hz, 2H), 4.72 (sept, J = 6.3 Hz, 1H), 2.43 (s, 3H), 1.26 (d, J = 6.3 Hz, 6H), ^{13}C NMR (75 MHz, $CDCl_3$) δ 144.5, 134.7, 129.9, 127.8, 77.2, 22.9, 21.7; LRMS calcd for $C_{10}H_{15}O_3S$ $(M+H)^+$: 215; found: 215.

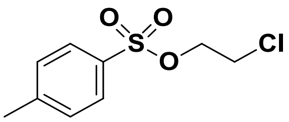

2-Chloroethyl 4-methylbenzenesulfonate (4f)[5]: was obtained by silica gel chromatography with a mixture of EtOAc/hexane (5:95) as an yellow oil: 0.102 mmol, 24.0 mg, 51% yield; ^{1}H NMR (300 MHz, $CDCl_3$) δ 7.81 (d, J = 8.3 Hz, 2H), 7.36 (d, J = 8.1 Hz,

---

[3] Miller, S. C. *J. Org. Chem.* **2010**, *75(13)*, 4632-4635
[4] Comagic, S.; Schirrmacher, R. *Synthesis* **2004**, *6*, 885-888
[5] Ameri David, R. L.; Kornfield, J. A. *Macromolecules* **2008**, *41*, 1151-1161

2H), 4.23 (t, *J* = 6.0 Hz, 2H), 3.65 (t, *J* = 6.0 Hz, 2H), 2.45 (s, 3H), ^{13}C NMR (75 MHz, $CDCl_3$) δ 145.4, 133.7, 130.1, 128.1, 69.0, 40.9, 21.8; LRMS calcd for $C_9H_{11}ClNaO_3S$ $(M+Na)^+$: 257; found: 257.

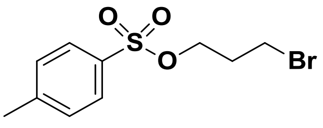

3-Bromopropyl 4-methylbenzenesulfonate (4g)[6]: was obtained by silica gel chromatography with a mixture of EtOAc/hexane (8:92) as a colorless oil: 0.102 mmol, 30.0 mg, 51% yield; ^{1}H NMR (300 MHz, $CDCl_3$) δ 7.79 (d, *J* = 8.3 Hz, 2H), 7.35 (d, *J* = 8.0 Hz, 2H), 4.17 (t, *J* = 5.8 Hz, 2H), 3.41 (t, *J* = 6.3 Hz, 2H), 2.45 (s, 3H), 2.23 – 2.11 (quin, *J* = 6.3 Hz, 2H), ^{13}C NMR (75 MHz, $CDCl_3$) δ 145.1, 132.8, 130.1, 128.1, 67.9, 31.9, 28.6, 21.8; LRMS calcd for $C_{10}H_{13}BrNaO_3S$ $(M+Na)^+$: 315; found: 315.

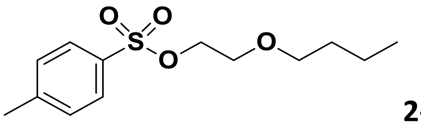

2-Butoxyethyl 4-methylbenzenesulfonate (4h)[7]: was obtained by silica gel chromatography with a mixture of EtOAc/hexane (15:85) as a colorless oil: 0.114 mmol, 31.0 mg, 57% yield; ^{1}H NMR (300 MHz, $CDCl_3$) δ 7.79 (d, *J* = 8.3 Hz, 2H), 7.33 (d, *J* = 8.0 Hz, 2H), 4.15 (dd, *J* = 5.4, 4.3 Hz, 2H), 3.59 (dd, *J* = 5.4, 4.3 Hz, 2H), 3.37 (t, *J* = 6.5 Hz, 2H), 2.44 (s, 3H), 1.53– 1.37 (m, 2H), 1.37 – 1.20 (m, 2H), 0.88 (t, *J* = 7.3 Hz, 3H); ^{13}C NMR (75 MHz, $CDCl_3$) δ 144.9, 133.2, 129.9, 128.1, 71.4, 69.5, 68.2, 31.7, 21.8, 19.3, 14.0; LRMS calcd for $C_{13}H_{21}O_4S$ $(M+H)^+$: 273; found: 273.

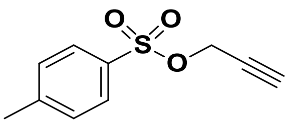

Prop-2-yn-1-yl 4-methylbenzenesulfonate (4i)[8]: was prepared without dichloromethane and was obtained by silica gel chromatography with a mixture of EtOAc/hexane (15:85) as a colorless oil: 0.162 mmol, 34.0 mg, 81% yield; ^{1}H NMR (300 MHz, $CDCl_3$) δ 7.84 (d, *J* = 8.3 Hz, 2H), 7.37 (d, *J* = 8.0 Hz, 2H), 4.72 (d, *J* = 2.5 Hz, 2H), 2.50 (t, *J* = 2.5 Hz, 1H), 2.48 (s, 3H); ^{13}C NMR (75 MHz, $CDCl_3$) δ 145.4, 133.0, 130.0, 128.3, 77.4, 75.5, 57.5, 21.8; LRMS calcd for $C_{10}H_{11}O_3S$ $(M+H)^+$: 211; found: 211.

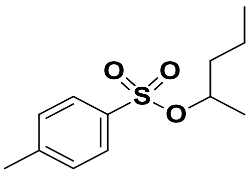

Pentan-2-yl 4-methylbenzenesulfonate (4j)[9]: was obtained by silica gel chromatography with a mixture of EtOAc/hexane (2:98) as a colorless oil: 0.120 mmol, 29.0

[6] Hromatka, O.; Stehlik, G.; Sauter, F. *MonatsheftefuerChemie* **1960**, *91*, 107-116

[7] Hermet, P.; Lois-Sierra, S.; Bantignies, J.-L.; Rols, S.; Sauvajol, J.-L.; Serein-Spirau, F.; Lère-Porte, J.-P.; Moreau, J. J. E. *J. Phys. Chem. B*. **2009**, *113*, 4197–4202

[8] Asano, K.; Matsubara, S. *Org. Lett.*, **2009**, *11*(8), 1757-1759

[9] Roque Pena, J. E., Alexanian, E. J. *Org. Lett.* **2017**, *19(17)*, 4413-4415

mg, 60% yield; ^1H NMR (300 MHz, $CDCl_3$) δ 7.79 (d, *J* = 8.3 Hz, 2H), 7.33 (d, *J*= 8.0 Hz, 2H), 4.63 (sext, *J* = 6.2 Hz, 1H), 2.44 (s, 3H), 1.70 – 1.37 (m, 2H), 1.37 – 1.15 (m, 5H), 0.82 (t, *J* = 7.3 Hz, 3H); ^{13}C NMR (75 MHz, $CDCl_3$) δ 144.5, 134.8, 129.8, 127.9, 80.6, 38.8, 21.8, 21.0, 18.3, 13.8; LRMS calcd for $C_{12}H_{19}O_3S$ $(M+H)^+$: 243; found: 243.

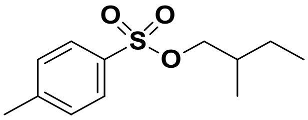

2-Methylbutyl 4-methylbenzenesulfonate (4k)[10]: was obtained by silica gel chromatography with a mixture of EtOAc/hexane (2:98) as an yellow oil: 0.152 mmol, 36.9 mg, 76% yield; ^1H NMR (300 MHz, $CDCl_3$) δ 7.78 (d, *J* = 8.3 Hz, 2H), 7.33 (d, *J* = 8.0 Hz, 2H), 3.84 (qd, *J* = 9.4, 6.1 Hz, 2H), 2.44 (s, 3H), 1.77 – 1.59 (m, 1H), 1.46 – 1.28 (m, 1H), 1.22 – 1.05 (m, 1H), 0.87 (d, *J* = 6.8 Hz, 3H), 0.82 (t, *J* = 7.4 Hz, 3H); ^{13}C NMR (75 MHz, $CDCl_3$) δ 144.7, 133.3, 129.9, 128.0, 74.9, 34.4, 25.5, 21.7, 16.1, 11.1; LRMS calcd for $C_{12}H_{19}O_3S$ $(M+H)^+$: 243; found: 243.

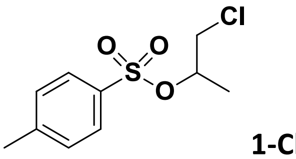

1-Chloropropan-2-yl 4-methylbenzenesulfonate (4l)[11]: was obtained obtained by silica gel chromatography with a mixture of EtOAc/hexane (5:95) as a colorless oil: 0.129 mmol, 32.0 mg, 65% yield; ^1H NMR (300 MHz, $CDCl_3$) δ 7.81 (d, *J* = 8.4 Hz, 2H), 7.35 (d, *J* = 8.0 Hz, 2H), 4.73 (sext, *J* = 6.0 Hz, 1H), 3.53 (qd, *J* = 11.7, 5.4 Hz, 2H), 2.45 (s, 3H), 1.37 (d, *J* = 6.4 Hz, 3H); ^{13}C NMR (75 MHz, $CDCl_3$) δ 145.1, 133.8, 130.0, 128.0, 77.7, 46.5, 21.8, 18.7; LRMS calcd for $C_{10}H_{13}ClNaO_3S$ $(M+Na)^+$: 271; found: 271.

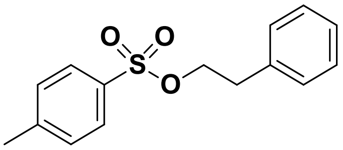

Phenylethyl 4-methylbenzenesulfonate (4m)[12]: was obtained by silica gel chromatography with a mixture of EtOAc/hexane (10/90) as a colorless oil: 0.10 mmol, 27 mg, 50% yield; ^1H NMR (300 MHz, $CDCl_3$) δ 7.72 (d, *J* = 8.3 Hz, 2H), 7.31 (d, *J* = 8.0 Hz, 2H), 7.28 - 7.24 (m, 3H), 7.14 (dd, *J* = 7.5, 1.8 Hz, 2H), 4.24 (t, *J* = 7.1 Hz, 2H), 2.98 (t, *J* = 7.1 Hz, 2H), 2.45 (d, *J* = 4.9 Hz, 3H), ^{13}C NMR (75 MHz, $CDCl_3$) δ 144.8, 136.3, 133.1, 129.9, 129.0, 128.7, 127.9, 127.0, 70.7, 35.4, 21.7. LRMS (ESI) calcd for $C_{15}H_{17}O_3S$ $(M + H)^+$: 277, found 277.

[10] Yang, Z.; Jin, X.; Guaciaro, M., Molino, B. F. *J. Org. Chem.* **2012**, *77*, 3191–3196
[11] Sletzinger, M.; Chamberlin, E. M.; Tishl, M. *J. Am. Chem. Soc.*, **1952**, *74*, 5619-5620
[12] Gao, J.; Pan, X.; Liu, J.; Lai, J.; Chang, L.; Yuan, G. *RSC Adv.*, **2015**, *5*, 27439

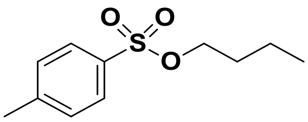

Butyl 4-methylbenzenesulfonate (4o)[1]: was obtained by silica gel chromatography with a mixture of EtOAc/hexane (10/90) as a colorless oil: 0.14 mmol, 32 mg, 70% yield; ^{1}H NMR (300 MHz, $CDCl_3$) δ 7.79 (d, *J* = 8.3 Hz, 2H), 7.34 (d, *J* = 8.0 Hz, 2H), 4.03 (t, *J* = 6.5 Hz, 2H), 2.45 (s, 3H), 1.68 – 1.56 (m, 2H), 1.34 (m, 2H), 0.86 (t, *J* = 7.4 Hz, 3H), ^{13}C NMR (75 MHz, $CDCl_3$) δ 144.8, 133.3, 129.9, 128.0, 70.5, 30.9, 21.7, 18.7, 13.5. LRMS (ESI) calcd for $C_{11}H_{17}O_3S$ $(M + H)^+$: 229, found 229.

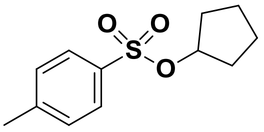

Cyclopentyl 4-methylbenzenesulfonate (4p)[9]:was obtained by silica gel chromatography with a mixture of EtOAc/hexane (2:98) as a colorless foam: 0.125 mmol, 30.0 mg, 63% yield; ^{1}H NMR (300 MHz, $CDCl_3$) δ 7.78 (d, *J* = 8.3 Hz, 2H), 7.33 (d, *J* = 8.0 Hz, 2H), 5.01 – 4.90 (m, 1H), 2.45 (s, 3H), 1.89 – 1.64 (m, 6H), 1.60 – 1.44 (m, 2H); ^{13}C NMR (75 MHz, $CDCl_3$) δ 144.5, 134.7, 129.9, 127.8, 85.6, 33.2, 23.2, 21.7; LRMS calcd for $C_{12}H_{17}O_3S$ $(M+H)^+$: 241; found: 241.

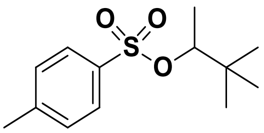

3,3-Dimethylbutan-2-yl 4-methylbenzenesulfonate (6)[13]: was obtained by silica gel chromatography with a mixture of EtOAc/hexane (98:2) as an yellow foam: 0.090 mmol, 23.0 mg, 45% yield; ^{1}H NMR (300 MHz, $CDCl_3$) δ 7.80 (d, *J* = 8.3 Hz, 2H), 7.33 (d, *J* = 8.0 Hz, 2H), 4.40 (q, *J* = 6.4 Hz, 1H), 2.45 (s, 3H), 1.22 (d, *J* = 6.4 Hz, 3H), 0.85 (s, 9H); ^{13}C NMR (75 MHz, $CDCl_3$) δ 144.4, 134.9, 129.8, 127.9, 87.8, 35.0, 25.7, 21.6, 16.1; LRMS Calc. for $C_{13}H_{20}NaO_3S$ $(M+Na)^+$: 279; found: 279.

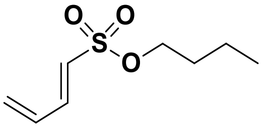

(*E*)-Butyl buta-1,3-diene-1-sulfonate (8a): was obtained by silica gel chromatography with a mixture of EtOAc/hexane (90/10) as a colorless oil: 0.14 mmol, 27 mg, 72% yield; ^{1}H NMR (300 MHz, $CDCl_3$) δ 7.43 – 7.27 (m, 1H), 6.71 (t, *J* = 11.2 Hz, 1H), 6.09 (dd, *J* = 11.0, 0.7 Hz, 1H), 5.68 (s, 1H), 5.66 – 5.62 (m, 1H), 4.14 (t, *J* = 6.5 Hz, 2H), 1.77 – 1.64 (m, 2H), 1.42 (m, 2H), 0.93 (t, *J* = 7.4 Hz, 3H), ^{13}C NMR (75 MHz, $CDCl_3$) δ 143.2, 130.4, 129.1, 122.6, 70.5, 31.1, 18.8, 13.6; LRMS (ESI) calcd for $C_8H_{15}O_3S$ $(M + H)^+$ 191, found 191.

[13]Yoshida, Y.; Sakakura, Y.; Aso, N.; Okada, S.; Tanabe, Yo. *Tetrahedron* **1999**, *55 (8)*, 2183-2192

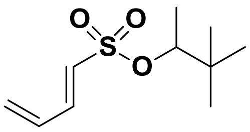

(*E*) 3,3-Dimethylbutan-2-yl buta-1,3-diene-1-sulfonate (8b): was obtained by silica gel chromatography with a mixture of EtOAc/hexane (10/90) as a colorless oil: 0.74 mmol, 16 mg, 37% yield; ^{1}H NMR (300 MHz, $CDCl_3$) δ 7.45 – 7.30 (m, 1H), 6.64 (t, *J* = 11.2 Hz, 1H), 6.12 (dd, *J* = 11.1, 0.8 Hz, 1H), 5.66 (d, *J* = 0.6 Hz, 1H), 5.61 (dd, *J* = 3.2, 2.4 Hz, 1H), 4.43 (q, *J* = 6.4 Hz, 1H), 1.36 (d, *J* = 6.4 Hz, 3H), 0.95 (s, 9H), ^{13}C NMR (75 MHz, $CDCl_3$) δ 141.8, 130.5, 128.6, 124.2, 88.0, 35.1, 25.8, 16.3; LRMS (ESI) calcd for $C_{10}H_{18}O_3SNa$ $(M + Na)^+$ 241, found 241.

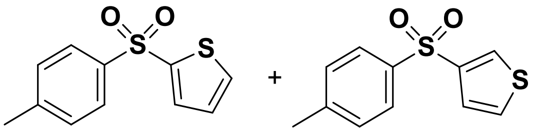

2-Tosylthiophene and 3-tosylthiophene (9a and 9b) : Iodobenzene diacetate (DIB, 0.24 mmol, 1.2 equiv) in solution of thiophene (1.0 mL) was added at room temperature to a vigorously stirred solution of thiophene (1.0 mL) with sulfinate (0.2 mmol, 1 equiv) and acetic acid (0.05 mL) to dissolve the sulfonate salt. The mixture was then stirred for 25 min (followed by TLC with a mixture of acetic acid/ethyl acetate/hexane) and then concentrated under vacuum. The residue was purified by silica gel chromatography with a mixture of (50/50) dichloromethane/hexane.

9a[14] was obtained as a white foam: 0.031mmol, 7.4 mg, 15% yield. ^{1}H NMR (300 MHz, $CDCl_3$) δ 7.87 (d, *J* = 8.1 Hz, 2H), 7.67 (dd, *J* = 3.9, 1.3 Hz, 1H), 7.62 (dd, *J* = 5.0, 1.5 Hz, 1H), 7.31 (d, *J* = 8.1 Hz, 2H), 7.06 (dd, *J* = 5.1, 3.9 Hz, 1H), 2.41 (s, 3H).^{13}C NMR (75 MHz, $CDCl_3$) δ 144.47, 143.68, 139.29, 133.68, 133.18, 130.09, 127.89, 127.54, 77.58, 77.16, 76.74, 21.75; LRMS (ESI) calcd for $C_{11}H_{11}O_2S_2$ $(M+H)^+$: 239; found: 239.

9b[15] was obtained as a white foam: 0.016mmol, 3.8 mg, 8% yield.^{1}H NMR (300 MHz, $CDCl_3$) δ 8.07 (dd, *J* = 3.0, 1.5 Hz, 1H), 7.84 (d, *J* = 8.1 Hz, 2H), 7.38-7.35 (m, 1H), 7.34 – 7.28 (m, 2H), 2.41 (s, 3H). ^{13}C NMR (75 MHz, $CDCl_3$) δ 144.41, 142.60, 138.84, 131.32, 130.07, 128.37, 127.68, 125.96, 77.58, 77.16, 76.74, 21.73; LRMS (ESI) calcd for $C_{11}H_{11}O_2S_2$ $(M+H)^+$: 239; found: 239.

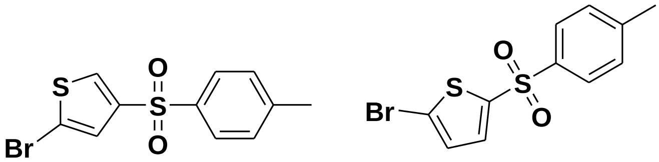

2-Bromo-4-tosylthiophene and 2-bromo-5-tosylthiophene (10a and 10b): Iodobenzene diacetate (DIB, 0.40 mmol, 2 equiv) in DCM

[14] Cacchi, S.; Fabrizi, G.; Goggiamani, A.; Parisi, L. M. *Org. Lett.* **2002**, *4*, 4719-4721
[15] Olah, G. A.; Kobayashi, S.; Nishimura, J. *J. Am. Chem. Soc*. **1973**, *95*, 564-569

(0.5 mL) was added to a vigorously stirred solution of sulfinate 1 (0.2 mmol, 1 equiv) in (0.5 mL) 2-bromothiophene and (0.5 mL) dichloromethane and trifluoroacetic acid (0.05 mL) was introduced to dissolve the sulfinate, at room temperature. The mixture was then stirred for 25 min (followed by TLC with a mixture of acetic acid/ethyl acetate/hexane) and then concentrated under vacuum. The residue was purified by silica gel chromatography with a mixture of 5/95 ethyl acetate/hexane. **10a** was obtained as a brown solid, 13 mg, 20% yield and **10b** 6mg 10% yield.

(10a): ^{1}H NMR (300 MHz, $CDCl_3$) δ 7.84 (d, *J* = 8.3 Hz, 2H), 7.42 (d, *J* = 4.0 Hz, 1H), 7.32 (d, *J* = 8.1 Hz, 2H), 7.03 (d, *J* = 4.0 Hz, 1H), 2.42 (s, 3H).^{13}C NMR (75 MHz, $CDCl_3$) δ 144.85, 144.46, 138.77, 133.20, 130.88, 130.23, 127.57, 121.90, 21.77. LRMS (ESI) calcd for $C_{11}H_{10}BrO_2S_2$ (M + H)$^+$: 316, found: 316.

(10b): ^{1}H NMR (300 MHz, $CDCl_3$) δ 7.90 (d, *J* = 8.3 Hz, 2H), 7.44 (d, *J* = 5.8 Hz, 1H), 7.33 (d, *J* = 8.0 Hz, 2H), 7.29 (d, *J* = 5.8 Hz, 1H), 2.43 (s, 3H); ^{13}C NMR (151 MHz, $CDCl_3$) δ 144.8, 140.4, 138.0, 129.9, 128.8, 128.1, 126.9, 117.7, 21.8; LRMS (ESI) calcd for $C_{11}H_{10}BrO_2S_2$ (M + H)$^+$: 316, found: 316.

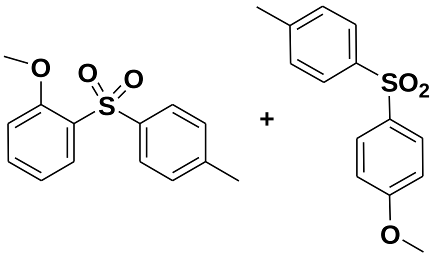

***ortho*-Tosylanisole and *para*-tosylanisole (11a and 11b):** Iodobenzene diacetate (DIB, 0.24 mmol, 1.2 equiv) in solution in (1.0 mL) of anisole was added to a vigorously stirred solution of sulfinate (0.2 mmol, 1 equiv) in anisole (1.0 mL) and acetic acid (0.05 mL) was introduced at room temperature. The mixture was then stirred for 25 min (followed by TLC with a mixture of acetic acid/ethyl acetate/hexane) and then concentrated under vacuum. The residue was purified by silica gel chromatography with a mixture of 5:95 ethyl acetate/hexane (for compound **11**). It was obtained as a white foam: 0.027mmol, (3.6 mg each) 7.2 mg, 14% total yield.

(11a) ^{1}H NMR (300 MHz, $CDCl_3$) δ 8.14 (dd, *J* = 6.3, 1.5 Hz, 1H), 7.85 (d, *J* = 8.4 Hz, 2H), 7.50 (dt, *J* = 5.7, 1.8 Hz, 1H), 7.28 (d, *J* = 8.4 Hz, 2H), 7.09 (dt, *J* = 7.5, 09 Hz, 1H), 6.89 (d, *J* = 8.7 Hz, 1H), 3.77 (s, 3H), 2.41 (s, 3H). ^{13}C NMR (75 MHz, $CDCl_3$) δ 157.19, 143.89, 138.73, 135.45, 129.95, 129.49, 129.26, 128.63, 120.64, 112.57, 77.58, 77.16, 76.74, 56.00, 21.74.

(11b) ^{1}H NMR (300 MHz, $CDCl_3$) δ 7.86 (d, *J* = 8.7 Hz, 2H), 7.79 (d, *J* = 8.4 Hz, 2H), 7.28 (d, *J* = 8.7 Hz, 2H), 3.83 (s, 3H), 2.38 (s, 3H). ^{13}C NMR (75 MHz, $CDCl_3$) δ 163.4, 143.9, 139.6, 133.7, 130.0, 129.9, 127.5, 114.6, 77.6, 77.2, 76.7, 55.8, 21.7. LRMS (ESI) calcd for $C_{14}H_{15}O_3S$ (M + H)$^+$: 263, found : 263.

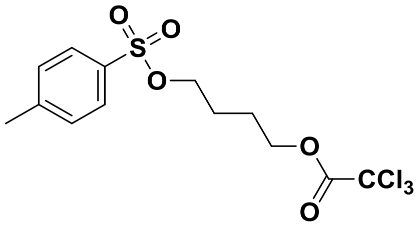

4-(Tosyloxy)butyl 2,2,2 trichloroacetate (12): Iodobenzene diacetate (DIB, 0.24 mmol, 1.2 equiv) in solution of tetrahydrofuran (1.0 mL) was added at room temperature to a vigorously stirred solution of tetrahydrofuran (1.0 mL) with sulfinate (0.2 mmol, 1 equiv) and trichloroacetic acid (0.05 mL) to dissolve the sulfonate salt. The mixture was then stirred for 25 min (followed by TLC with a mixture of acetic acid/ethyl acetate/hexane) and then concentrated under vacuum. The residue was purified by silica gel chromatography with a mixture of 5/95 ethyl acetate/hexane. **12** was obtained as a white foam: 0.80mmol, 31.4 mg, 40% yield; ^{1}H NMR (300 MHz, $CDCl_3$) δ 7.79 (d, J = 8.4 Hz, 2H), 7.35 (d, J = 8.1 Hz, 2H), 4.33 (t, J = 6.0 Hz, 2H), 4.09 (t, J = 5.8 Hz, 2H), 2.45 (s, 3H), 1.95 – 1.66 (m, 4H); ^{13}C NMR (75 MHz, $CDCl_3$) δ 162.02, 145.09, 133.07, 130.06, 128.03, 89.87, 77.58, 77.16, 76.74, 69.59, 68.52, 25.48, 24.65, 21.79.

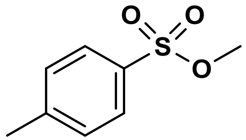

4a

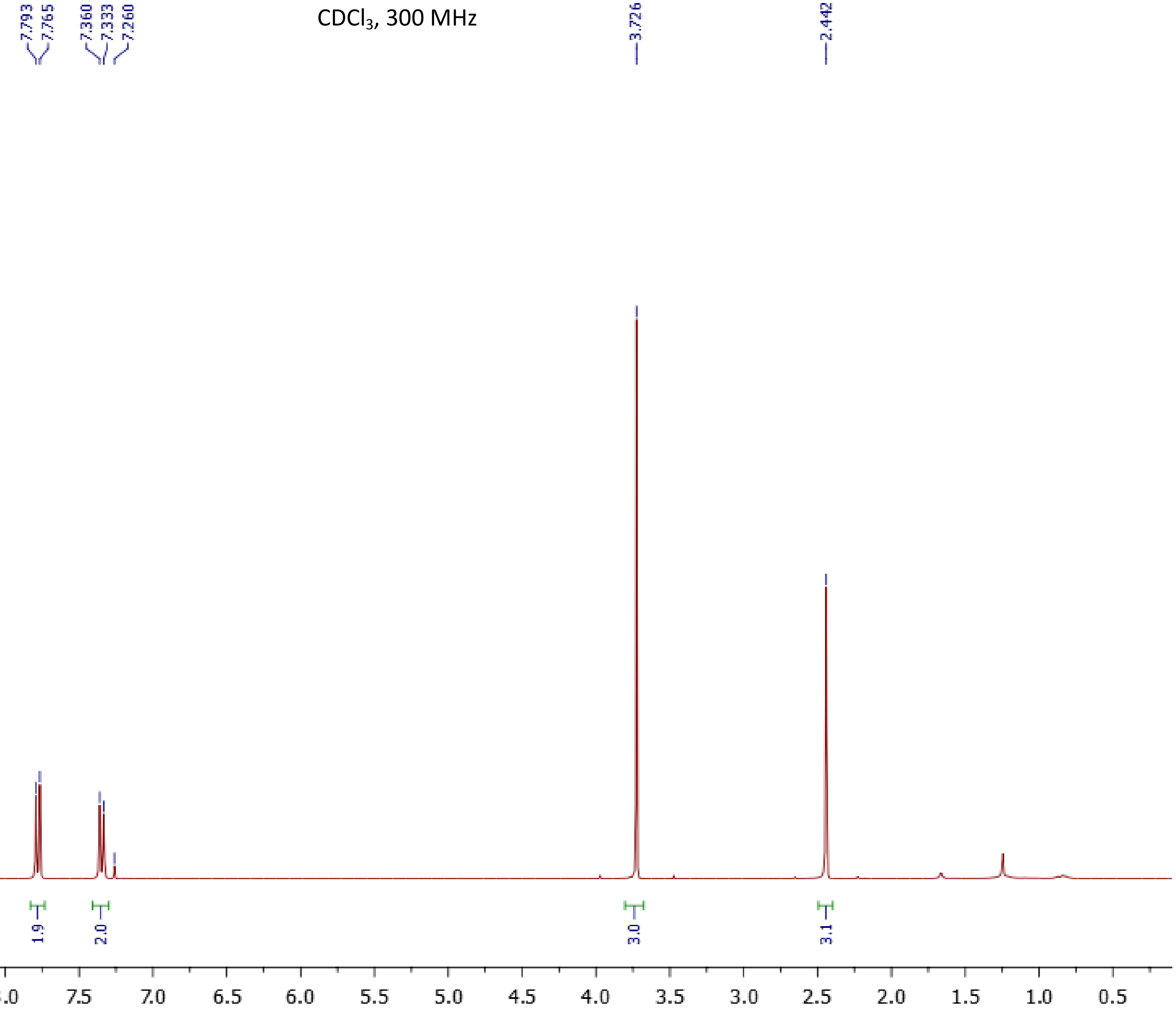
CDCl3, 300 MHz
7.793
7.765
7.360
7.333
7.260
3.726
2.442
1.9
2.0
3.0
3.1
10.0 9.5 9.0 8.5 8.0 7.5 7.0 6.5 6.0 5.5 5.0 4.5 4.0 3.5 3.0 2.5 2.0 1.5 1.0 0.5

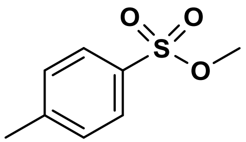

4a

CDCl$_3$, 75 MHz

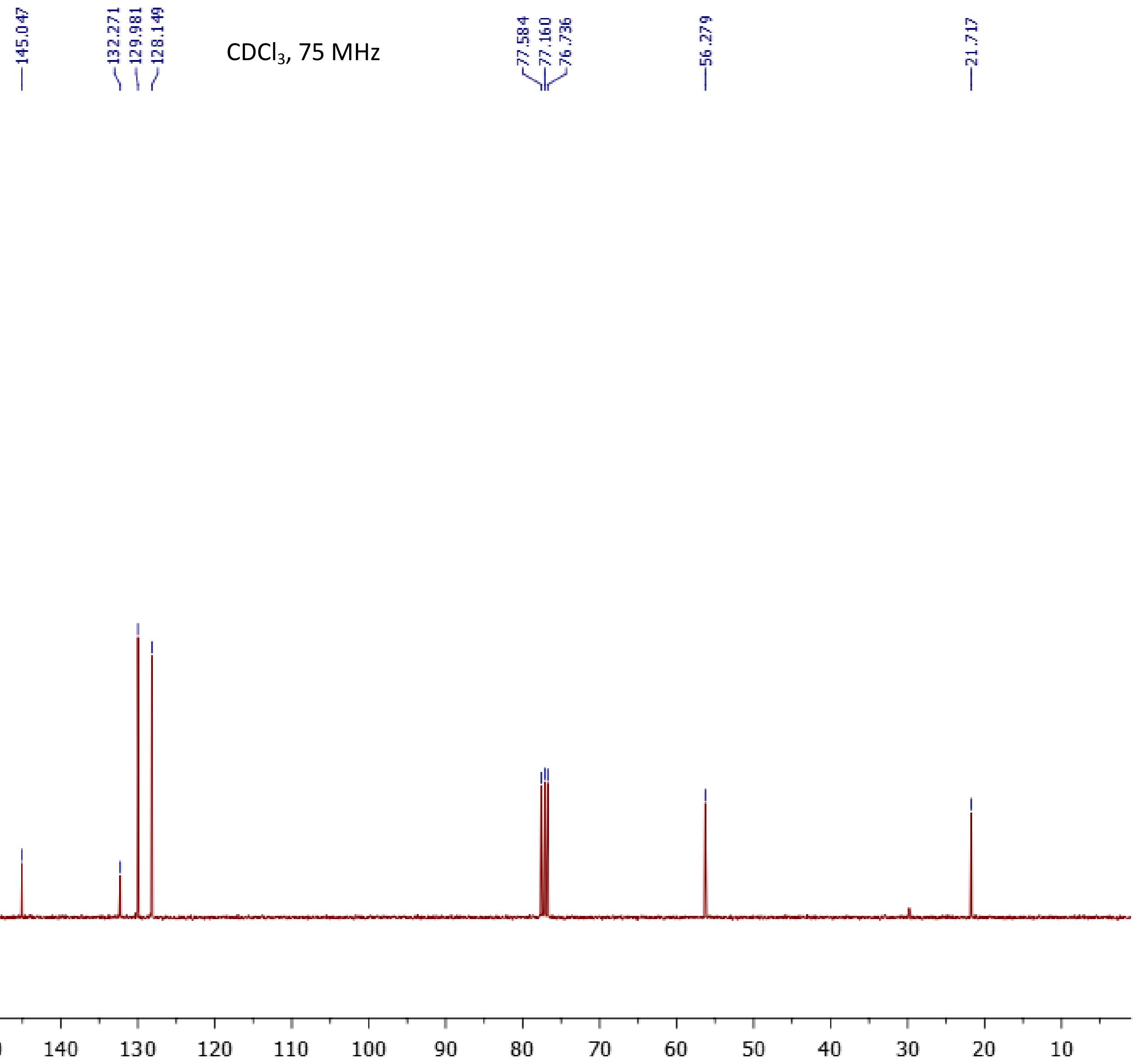

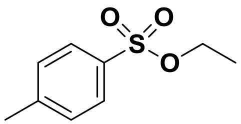

4b

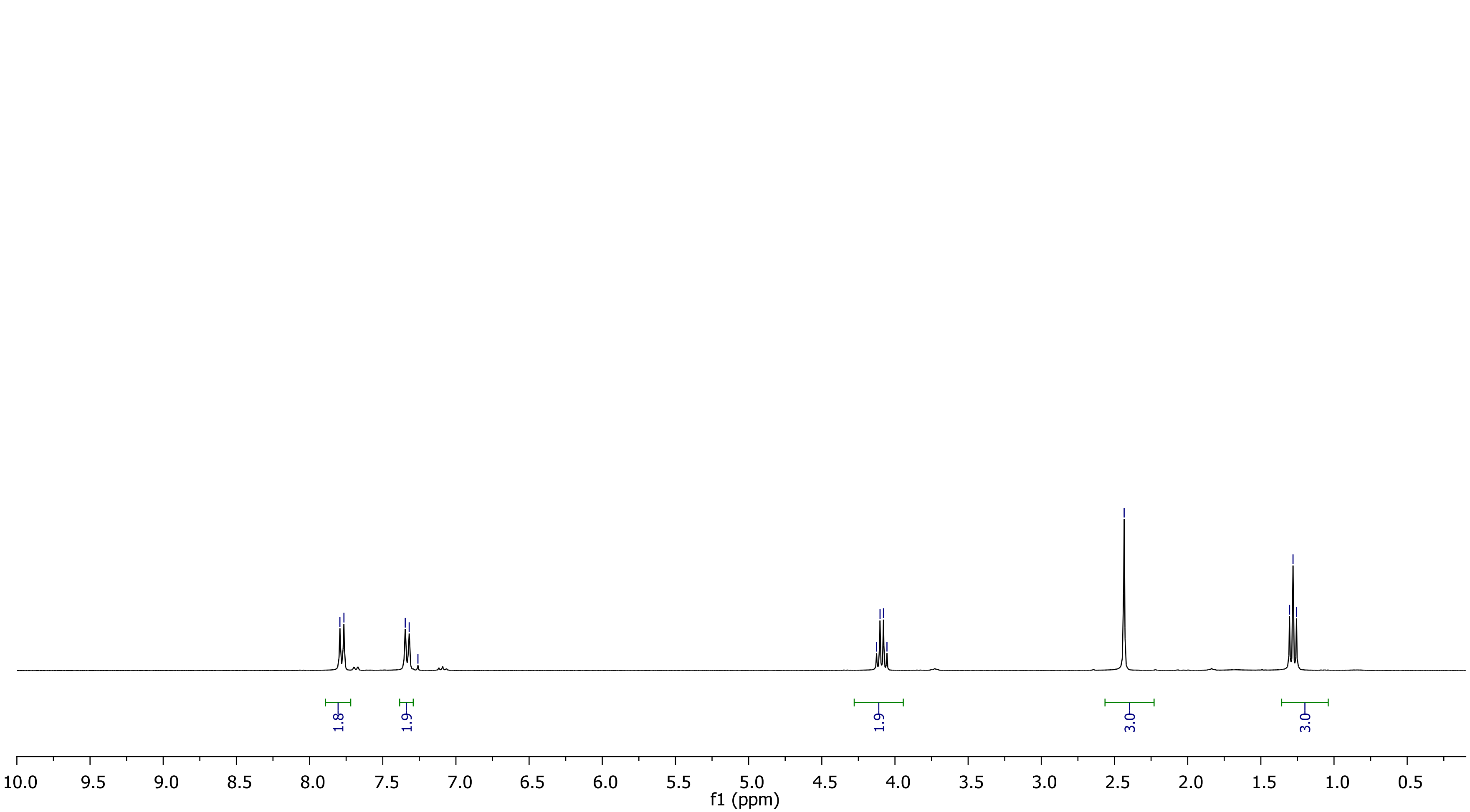
CDCl$_3$, 300 MHz
7.793
7.765
7.346
7.319
7.260
4.126
4.102
4.078
4.055
2.434
1.304
1.280
1.256
1.8
1.9
1.9
3.0
3.0
10.0 9.5 9.0 8.5 8.0 7.5 7.0 6.5 6.0 5.5 5.0 4.5 4.0 3.5 3.0 2.5 2.0 1.5 1.0 0.5
f1 (ppm)

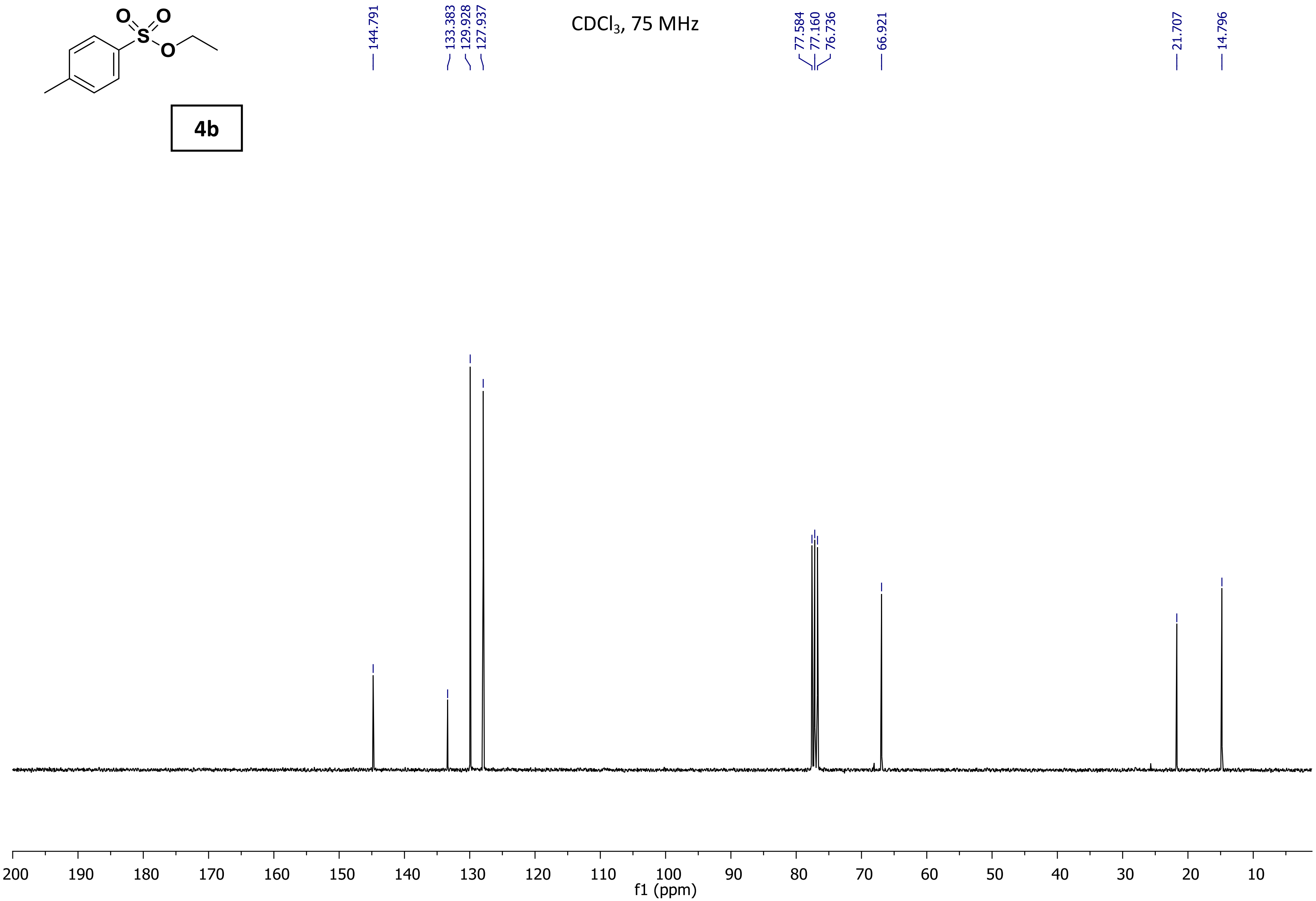

4b
CDCl3, 75 MHz
144.791
133.383
129.928
127.937
77.584
77.160
76.736
66.921
21.707
14.796
200 190 180 170 160 150 140 130 120 110 100 90 80 70 60 50 40 30 20 10
f1 (ppm)

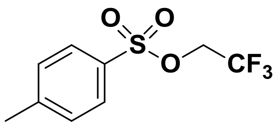

4c

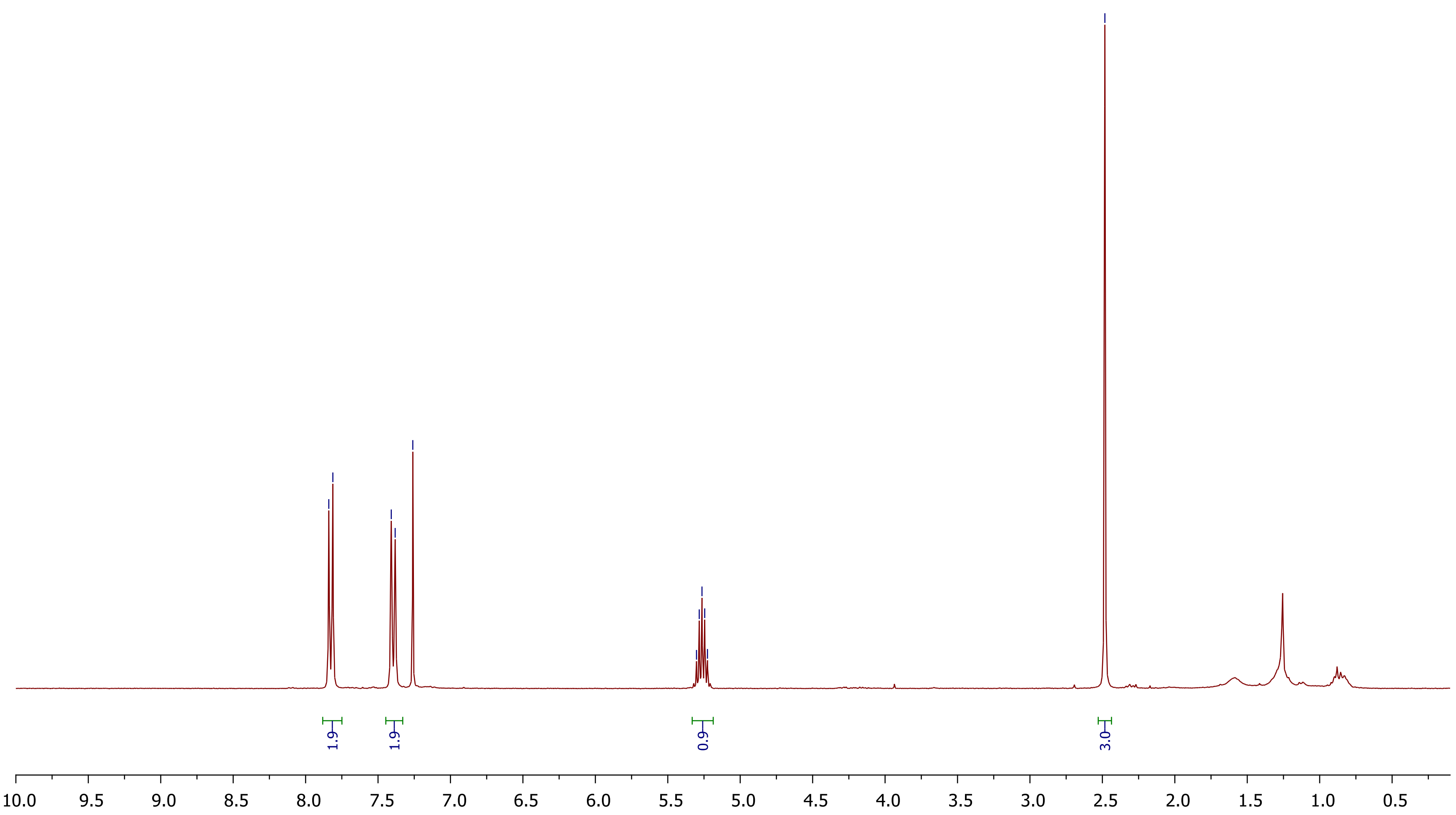

CDCl$_3$, 300 MHz
7.840
7.812
7.409
7.382
7.260
5.302
5.283
5.264
5.245
5.226
2.483
1.9
1.9
0.9
3.0
10.0 9.5 9.0 8.5 8.0 7.5 7.0 6.5 6.0 5.5 5.0 4.5 4.0 3.5 3.0 2.5 2.0 1.5 1.0 0.5

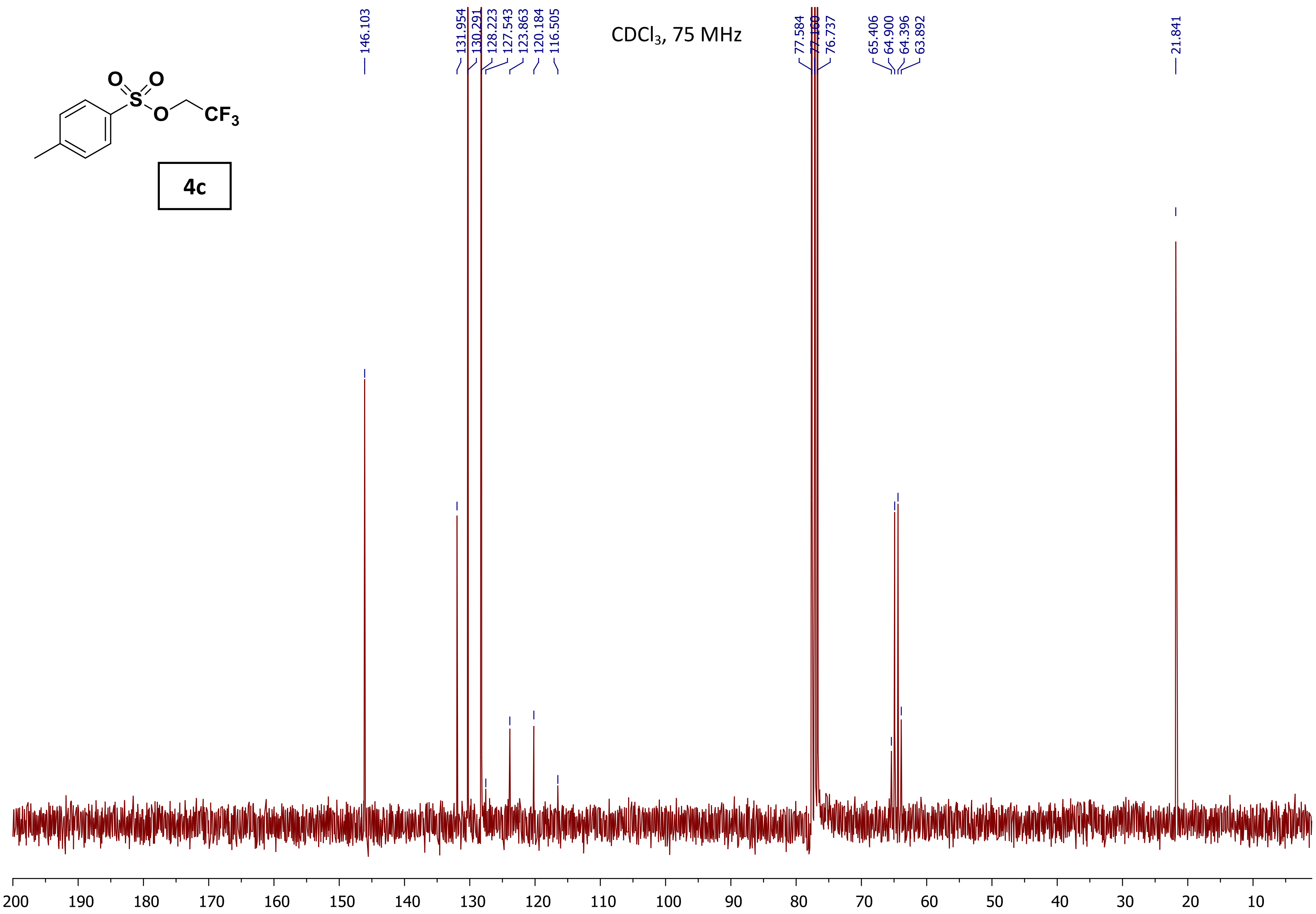

146.103
131.954
130.291
128.223
127.543
123.863
120.184
116.505
CDCl3, 75 MHz
77.584
77.160
76.737
65.406
64.900
64.396
63.892
21.841
200 190 180 170 160 150 140 130 120 110 100 90 80 70 60 50 40 30 20 10

4c

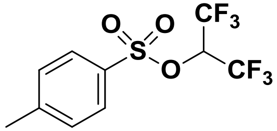
CF3
O
O
S
O
CF3

4d

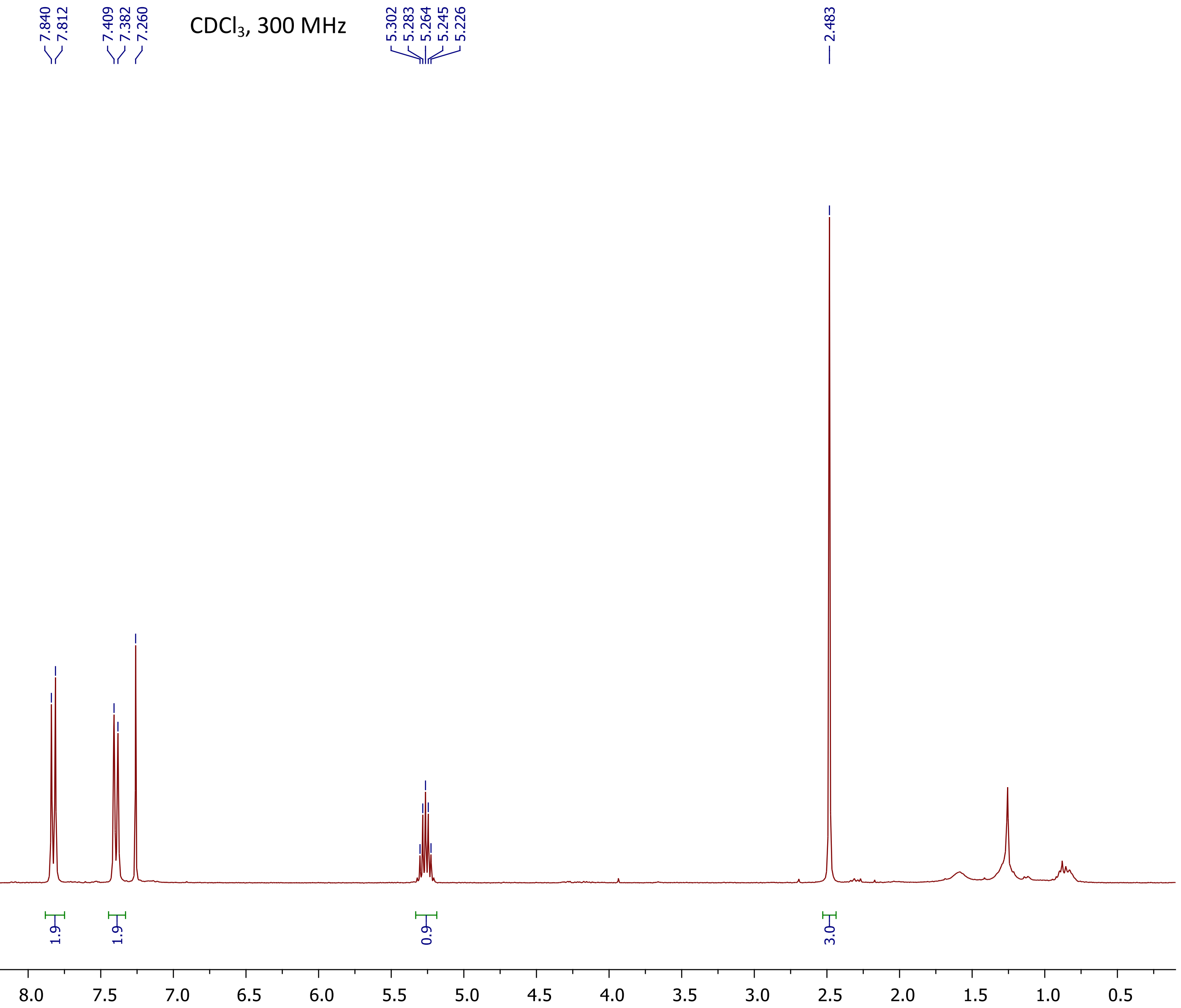
CDCl3, 300 MHz
7.840
7.812
7.409
7.382
7.260
5.302
5.283
5.264
5.245
5.226
2.483
1.9
1.9
0.9
3.0
10.0
9.5
9.0
8.5
8.0
7.5
7.0
6.5
6.0
5.5
5.0
4.5
4.0
3.5
3.0
2.5
2.0
1.5
1.0
0.5

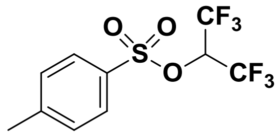

4d

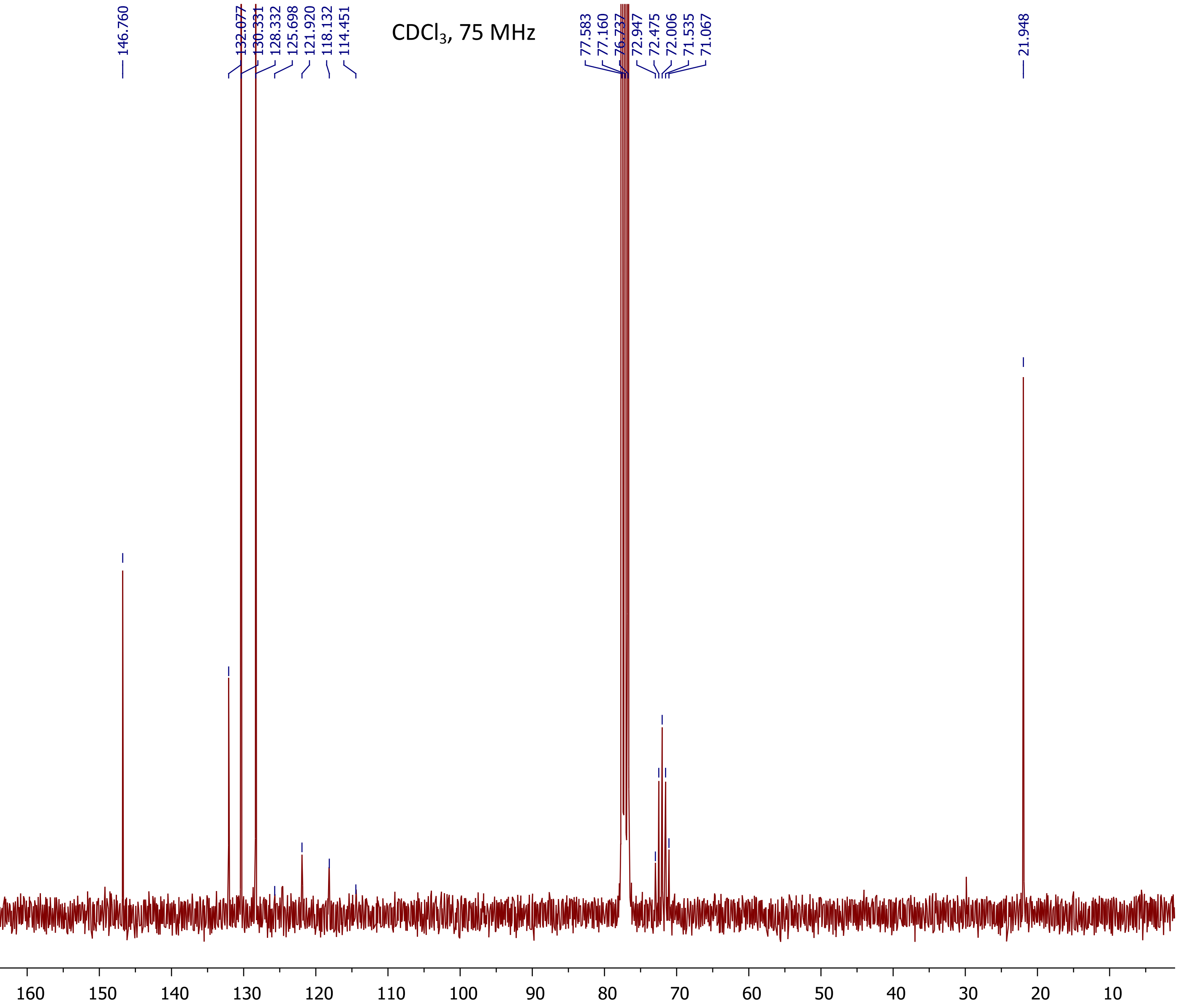
146.760
132.077
130.331
128.332
125.698
121.920
118.132
114.451
CDCl3, 75 MHz
77.583
77.160
76.737
72.947
72.475
72.006
71.535
71.067
21.948
200 190 180 170 160 150 140 130 120 110 100 90 80 70 60 50 40 30 20 10

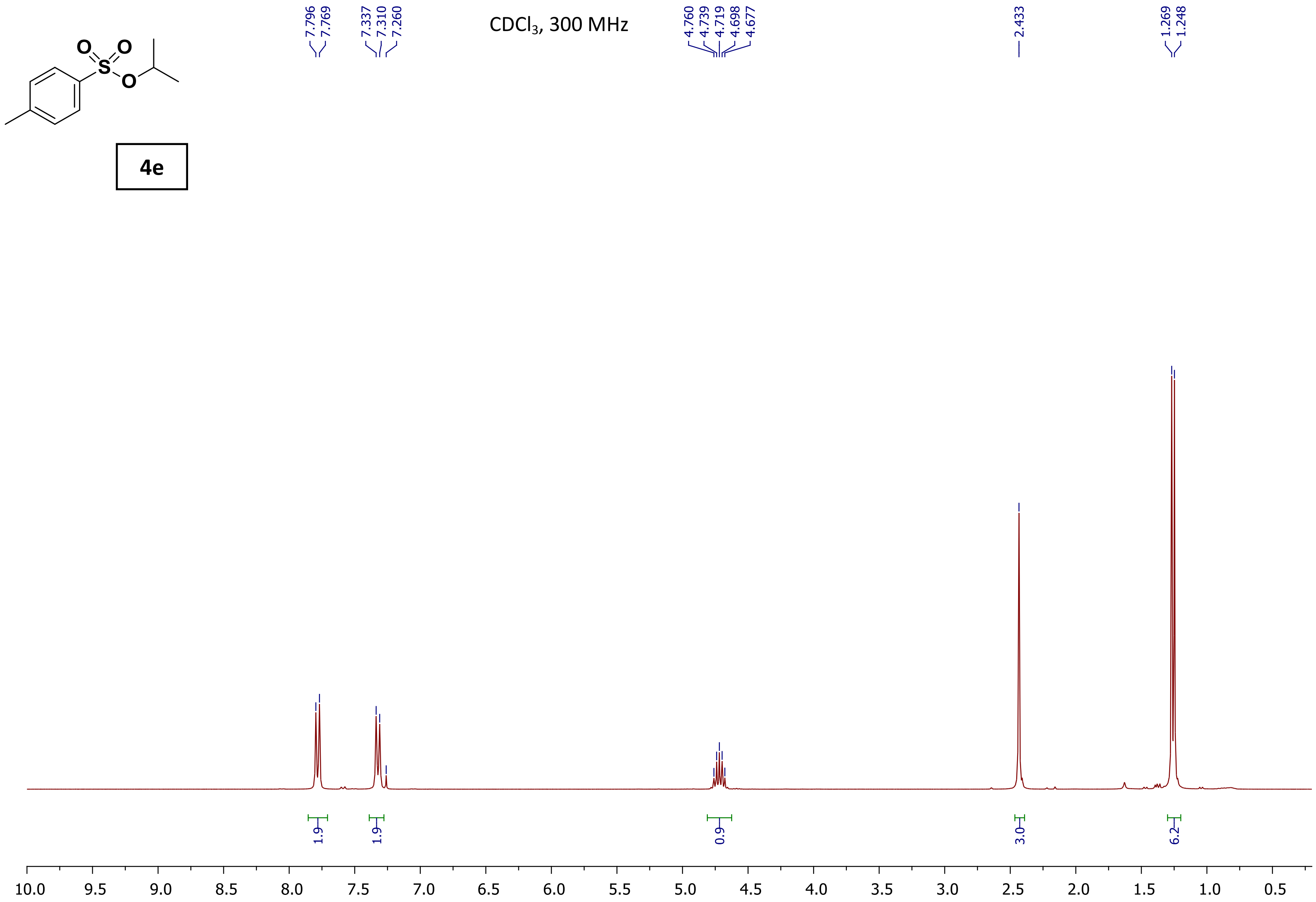

4e
CDCl$_3$, 300 MHz
7.796
7.769
7.337
7.310
7.260
4.760
4.739
4.719
4.698
4.677
2.433
1.269
1.248
1.9
1.9
0.9
3.0
6.2
10.0 9.5 9.0 8.5 8.0 7.5 7.0 6.5 6.0 5.5 5.0 4.5 4.0 3.5 3.0 2.5 2.0 1.5 1.0 0.5

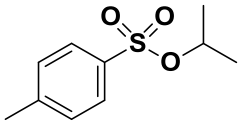

4e

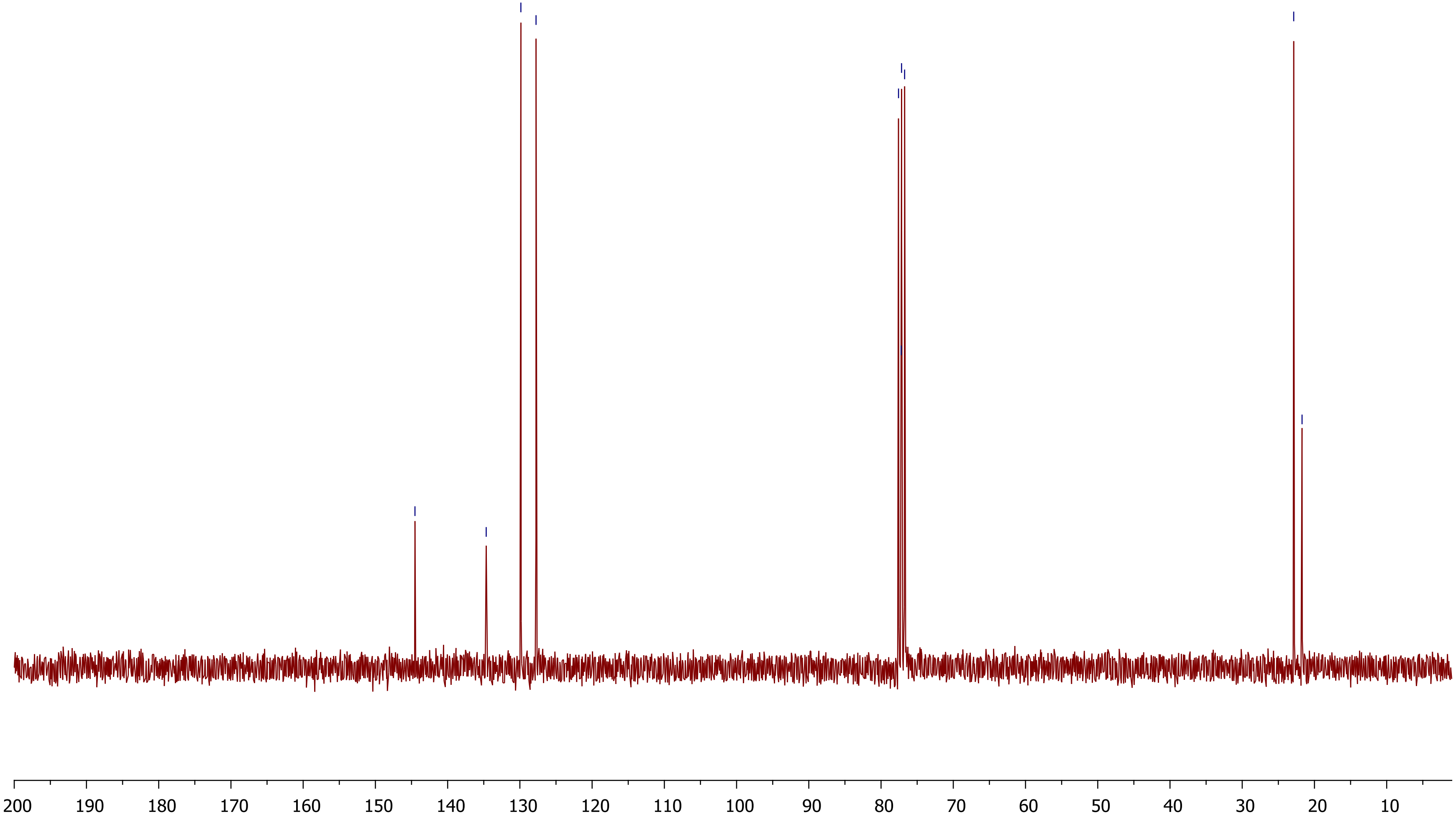
CDCl$_3$, 75 MHz
144.525
134.663
129.862
127.762
77.583
77.216
77.160
76.735
22.870
21.717
200 190 180 170 160 150 140 130 120 110 100 90 80 70 60 50 40 30 20 10

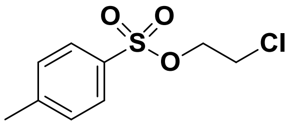

4f

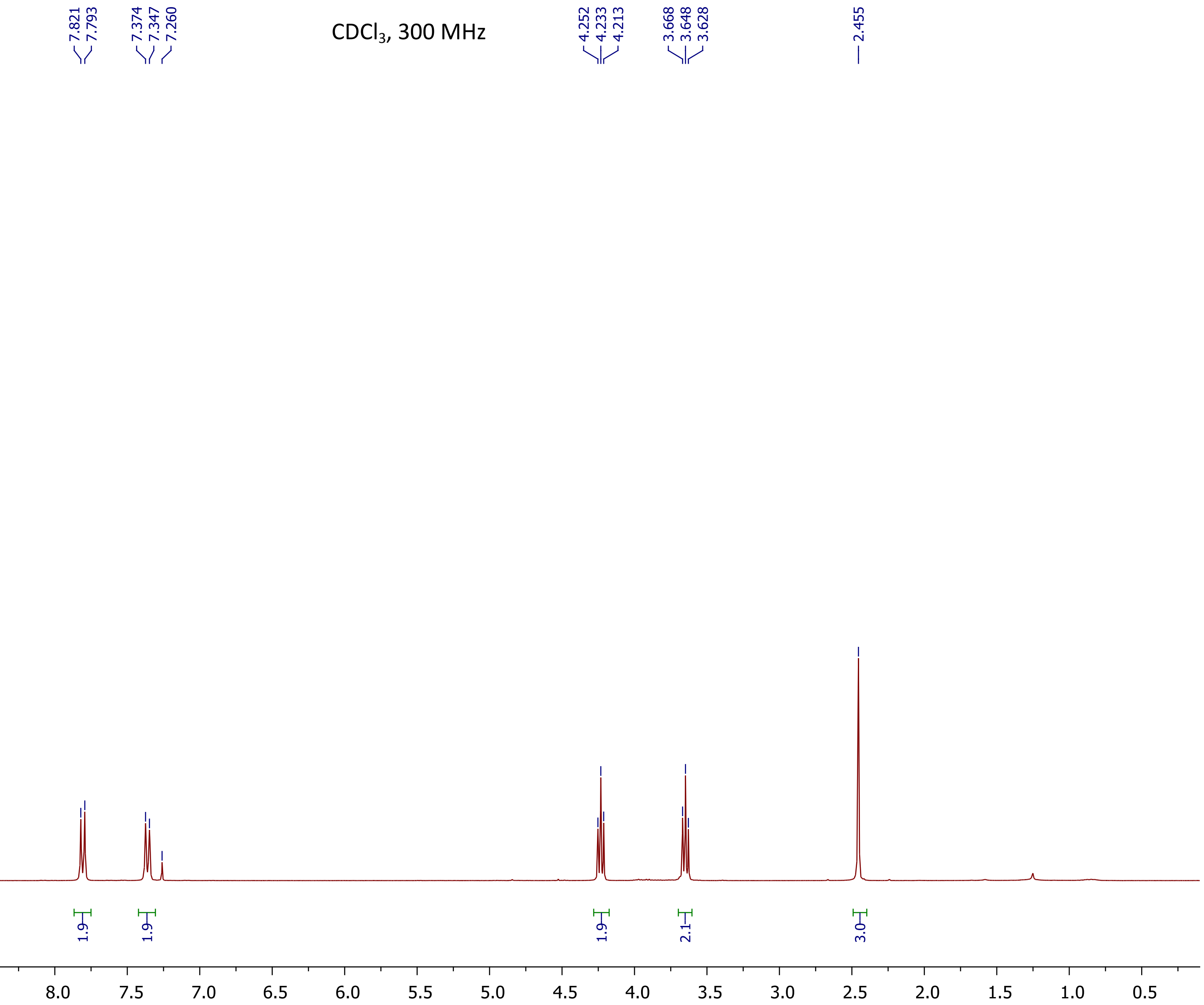

7.821
7.793
7.374
7.347
7.260
CDCl$_3$, 300 MHz
4.252
4.233
4.213
3.668
3.648
3.628
2.455
1.9
1.9
1.9
2.1
3.0
10.0 9.5 9.0 8.5 8.0 7.5 7.0 6.5 6.0 5.5 5.0 4.5 4.0 3.5 3.0 2.5 2.0 1.5 1.0 0.5

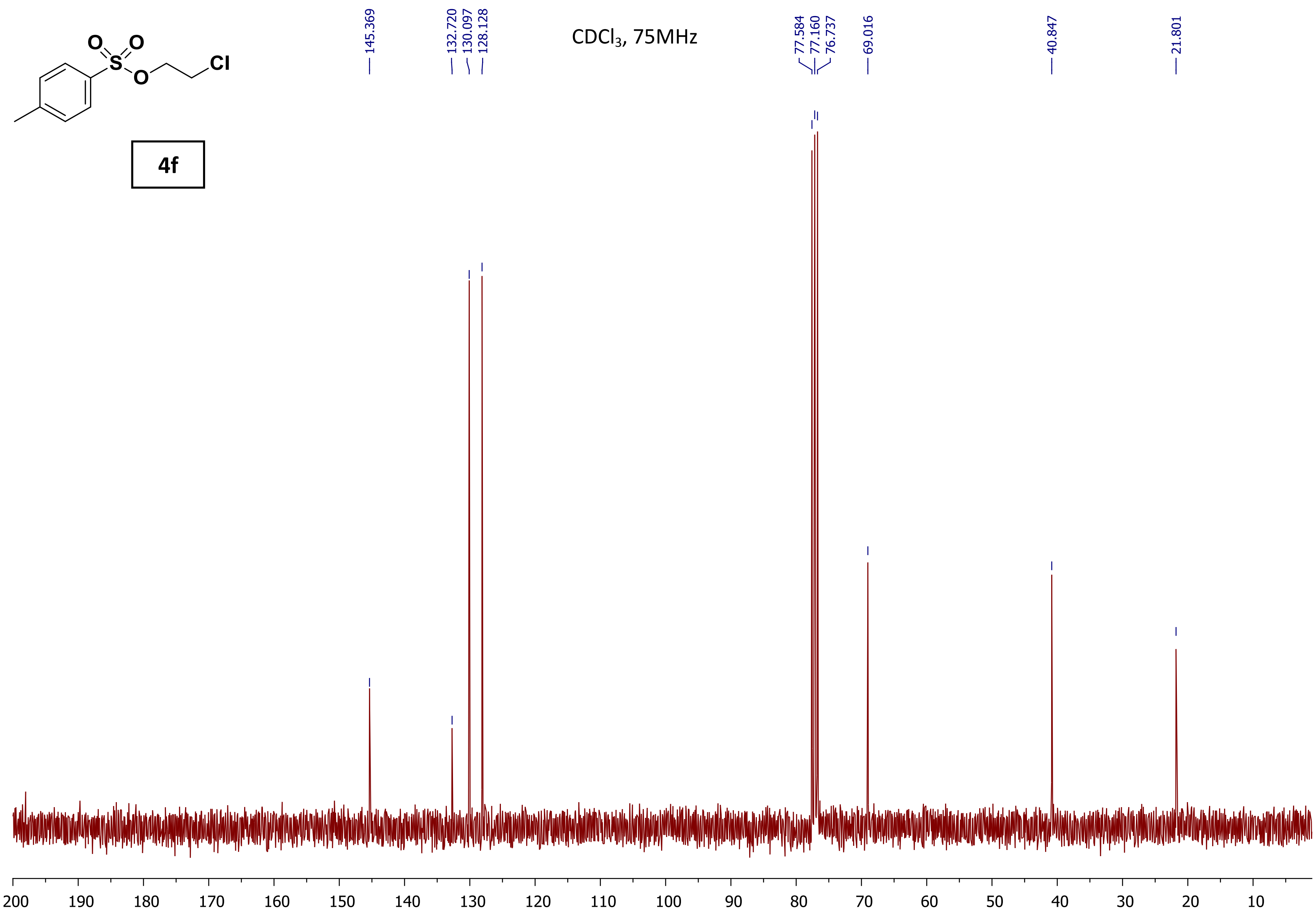
145.369
132.720
130.097
128.128
CDCl3, 75MHz
77.584
77.160
76.737
69.016
40.847
21.801
200
190
180
170
160
150
140
130
120
110
100
90
80
70
60
50
40
30
20
10

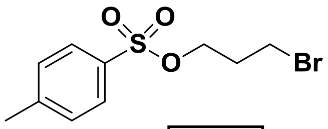
O
O
S
O
Br

4g

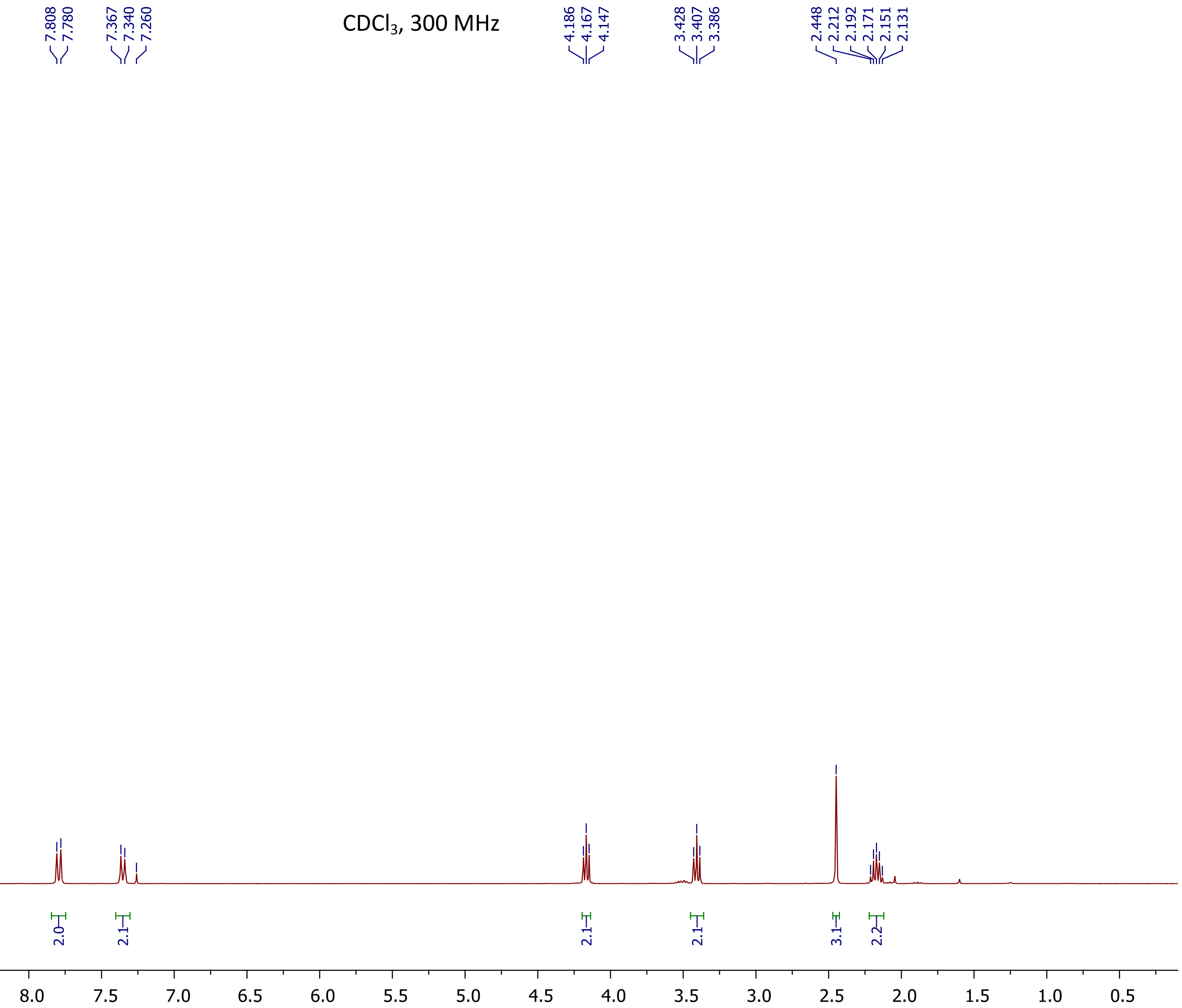
CDCl$_3$, 300 MHz
7.808
7.780
7.367
7.340
7.260
4.186
4.167
4.147
3.428
3.407
3.386
2.448
2.212
2.192
2.171
2.151
2.131
2.0
2.1
2.1
2.1
3.1
2.2
10.0
9.5
9.0
8.5
8.0
7.5
7.0
6.5
6.0
5.5
5.0
4.5
4.0
3.5
3.0
2.5
2.0
1.5
1.0
0.5

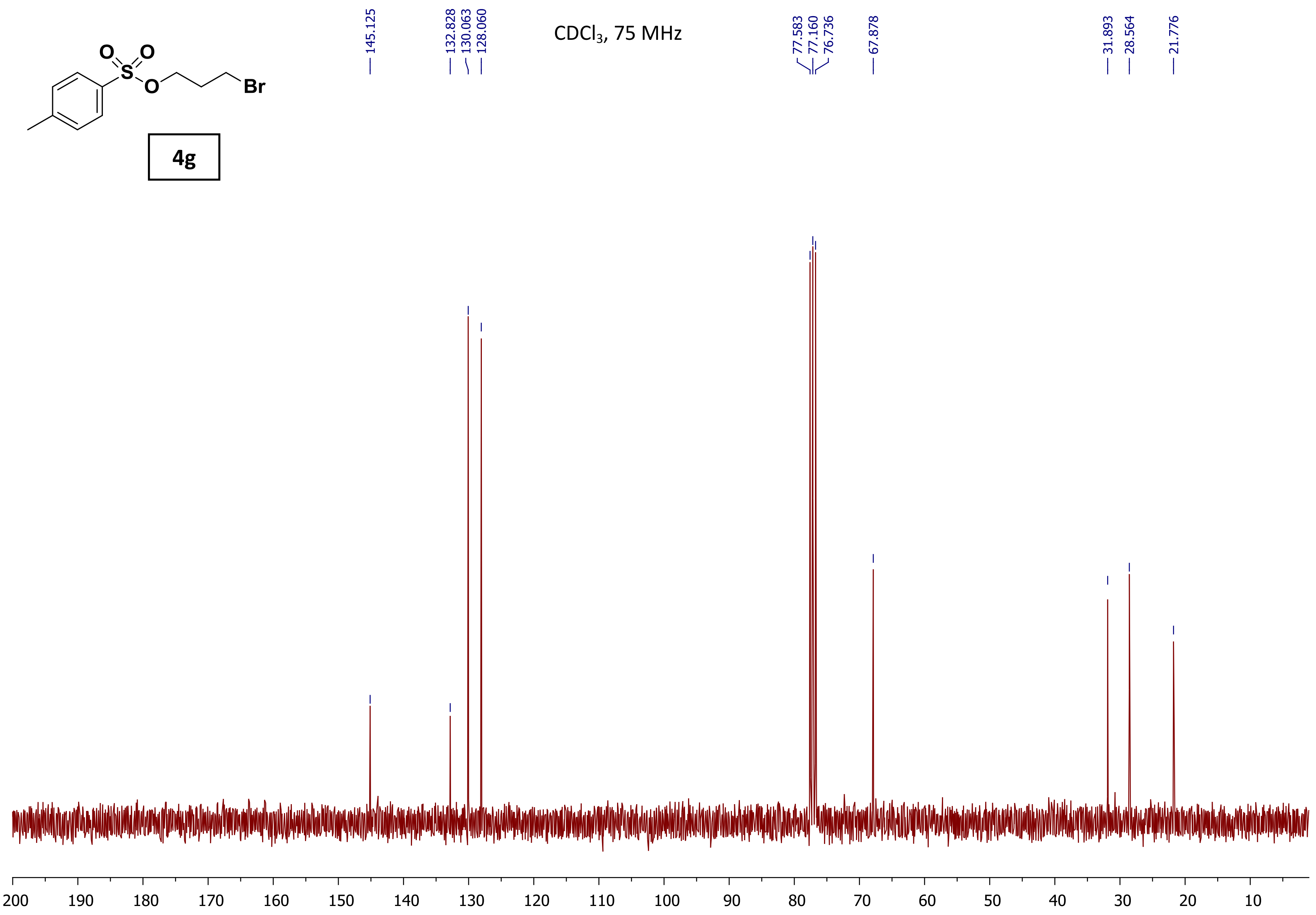
4g
145.125
132.828
130.063
128.060
CDCl$_3$, 75 MHz
77.583
77.160
76.736
67.878
31.893
28.564
21.776
200 190 180 170 160 150 140 130 120 110 100 90 80 70 60 50 40 30 20 10

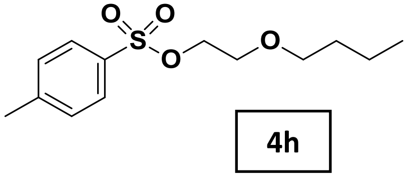

4h

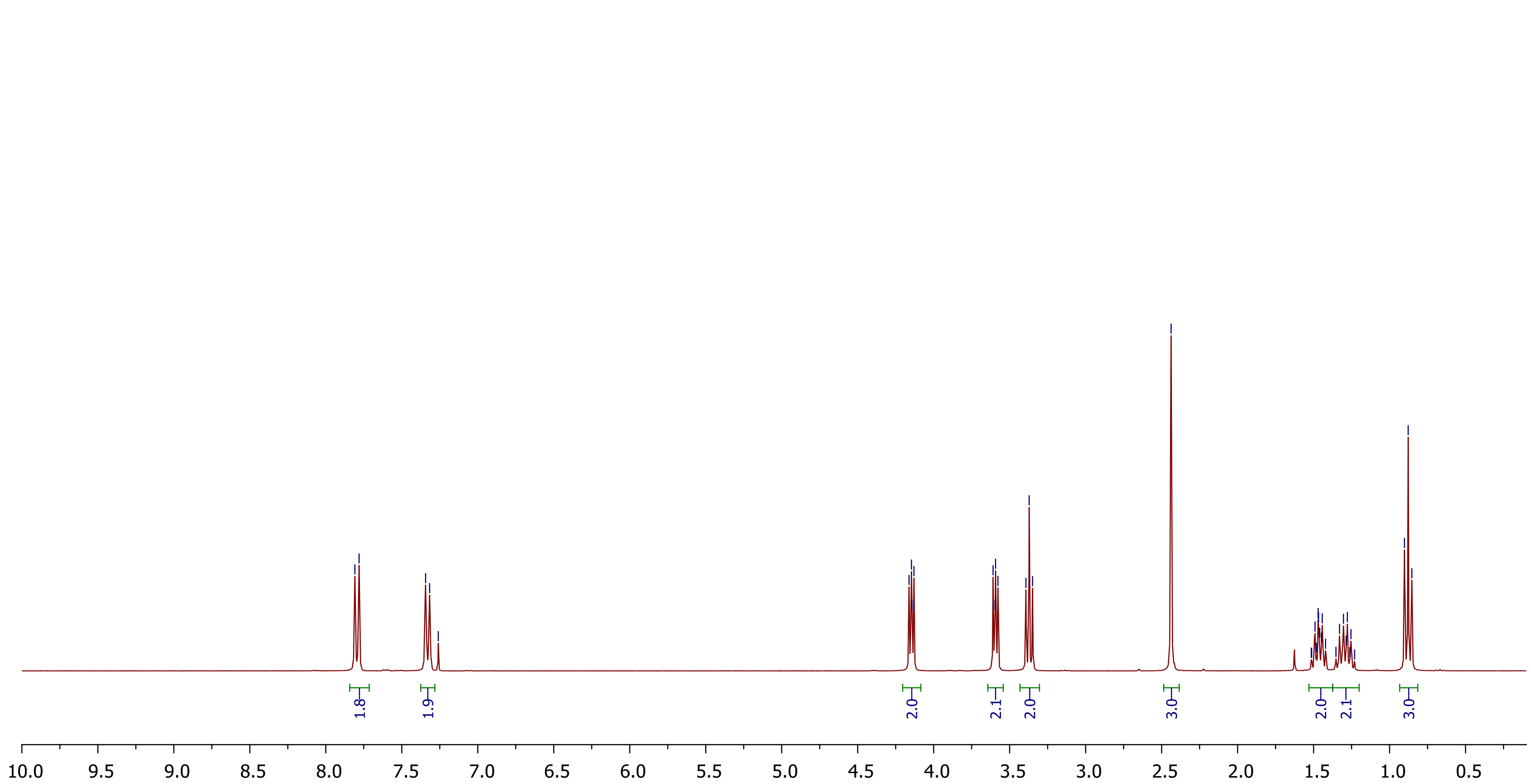
CDCl$_3$, 300 MHz
7.809
7.781
7.343
7.317
7.260
4.162
4.146
4.143
4.130
3.609
3.596
3.593
3.577
3.393
3.371
3.349
2.438
1.491
1.470
1.467
1.460
1.448
1.443
1.329
1.303
1.286
1.278
1.254
0.902
0.878
0.853
1.8
1.9
2.0
2.1
2.0
3.0
2.0
2.1
3.0
10.0 9.5 9.0 8.5 8.0 7.5 7.0 6.5 6.0 5.5 5.0 4.5 4.0 3.5 3.0 2.5 2.0 1.5 1.0 0.5

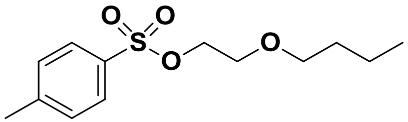

4h

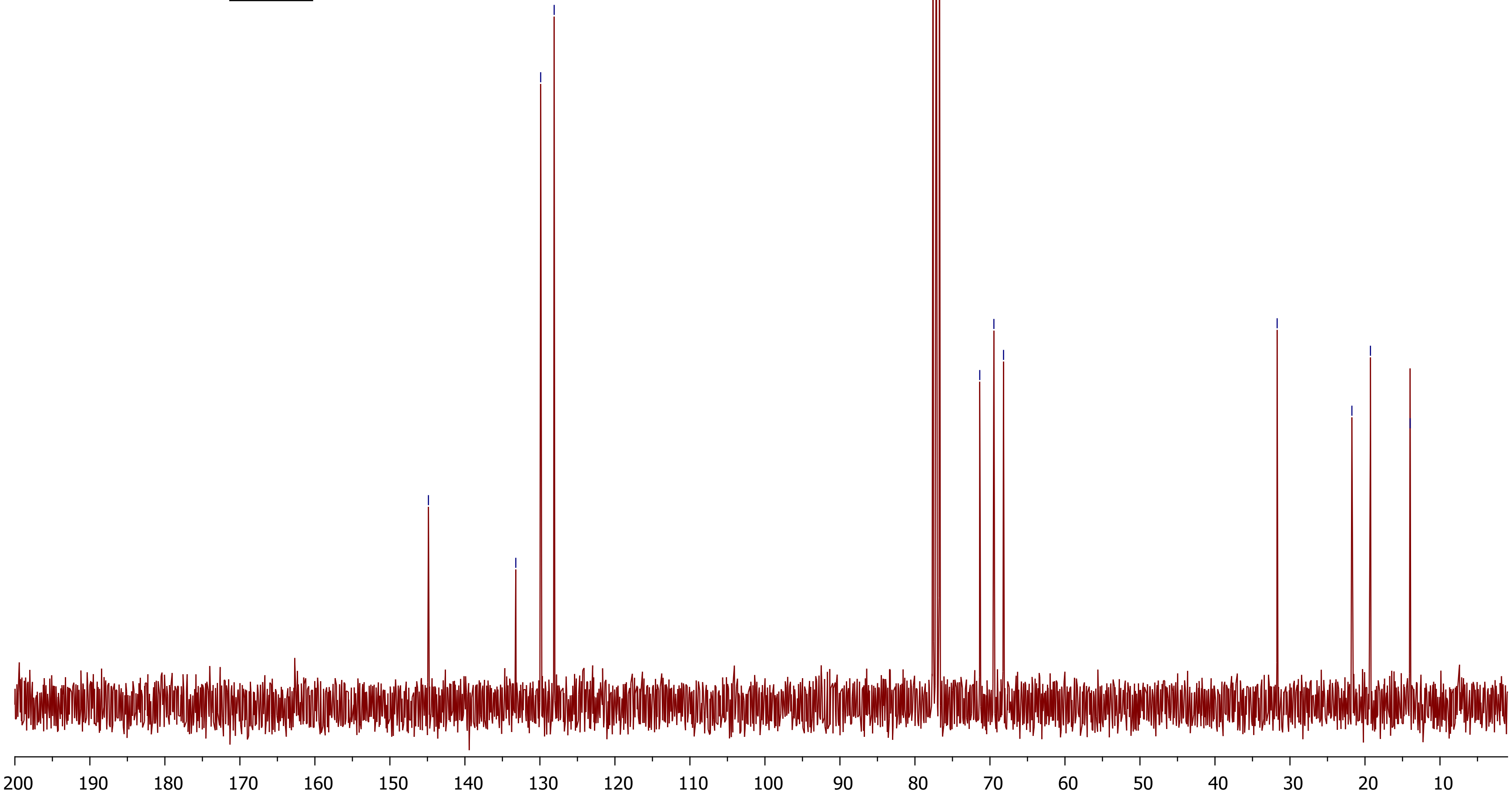

CDCl$_3$, 75 MHz
144.861
133.215
129.897
128.099
77.583
77.160
77.160
76.736
71.359
69.475
68.190
31.712
21.746
19.269
13.979
200 190 180 170 160 150 140 130 120 110 100 90 80 70 60 50 40 30 20 10

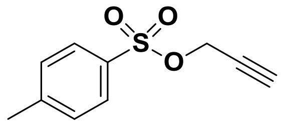

4i

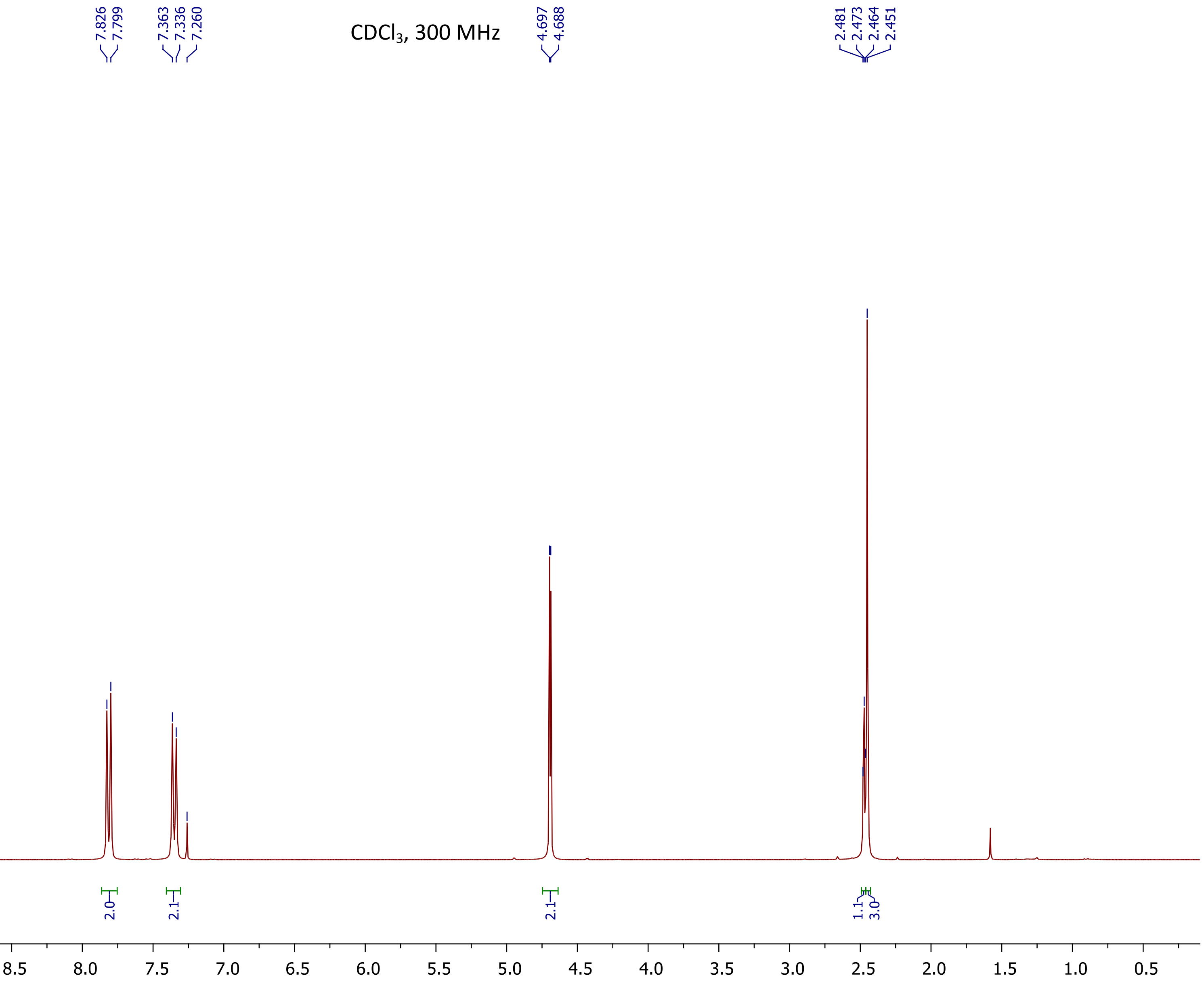
CDCl$_3$, 300 MHz
7.826
7.799
7.363
7.336
7.260
4.697
4.688
2.481
2.473
2.464
2.451
2.0
2.1
2.1
1.1
3.0
10.0 9.5 9.0 8.5 8.0 7.5 7.0 6.5 6.0 5.5 5.0 4.5 4.0 3.5 3.0 2.5 2.0 1.5 1.0 0.5

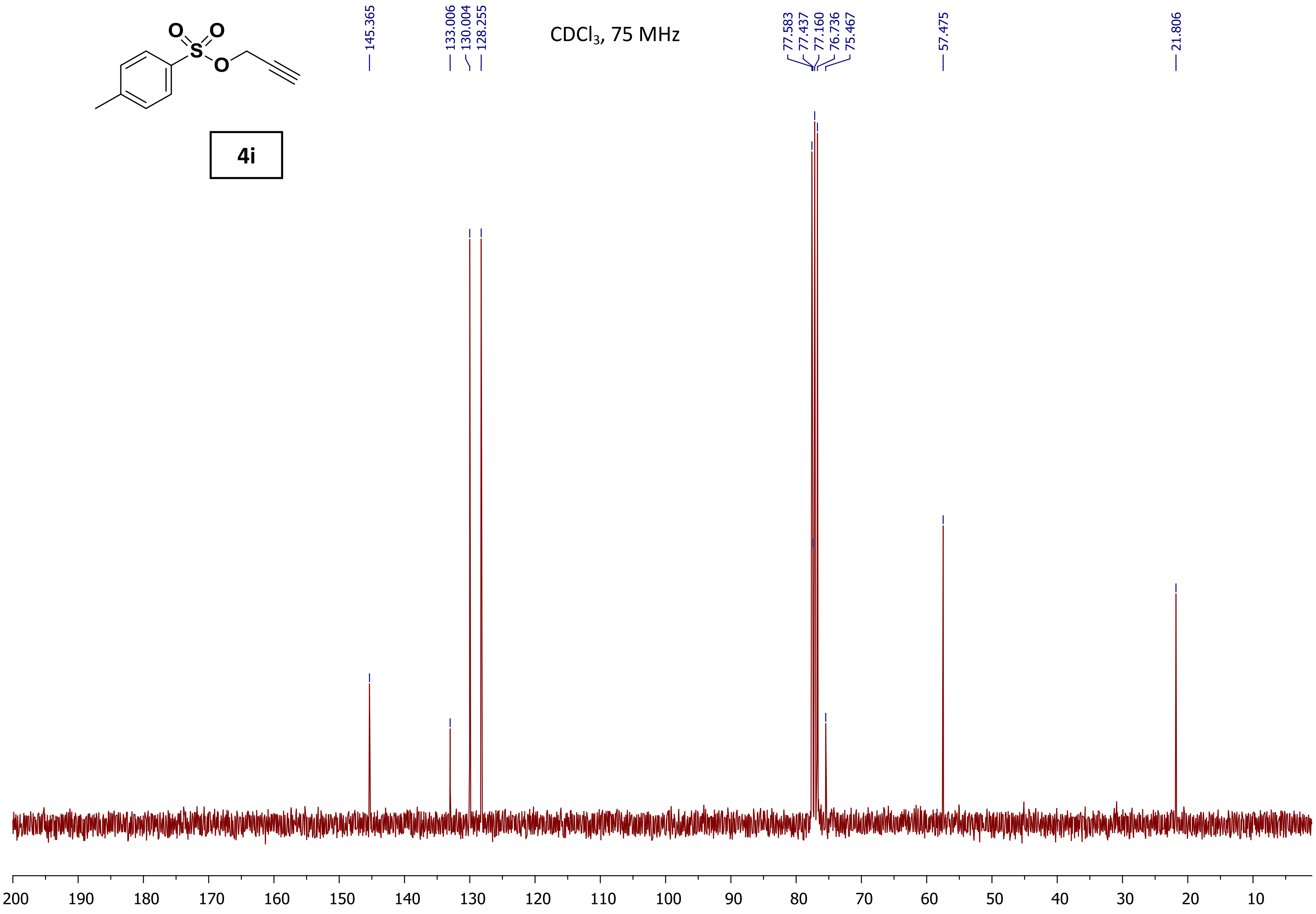

4i
CDCl3, 75 MHz
145.365
133.006
130.004
128.255
77.583
77.437
77.160
76.736
75.467
57.475
21.806
200 190 180 170 160 150 140 130 120 110 100 90 80 70 60 50 40 30 20 10

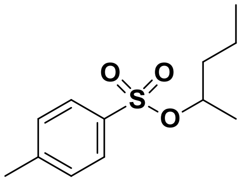

4j

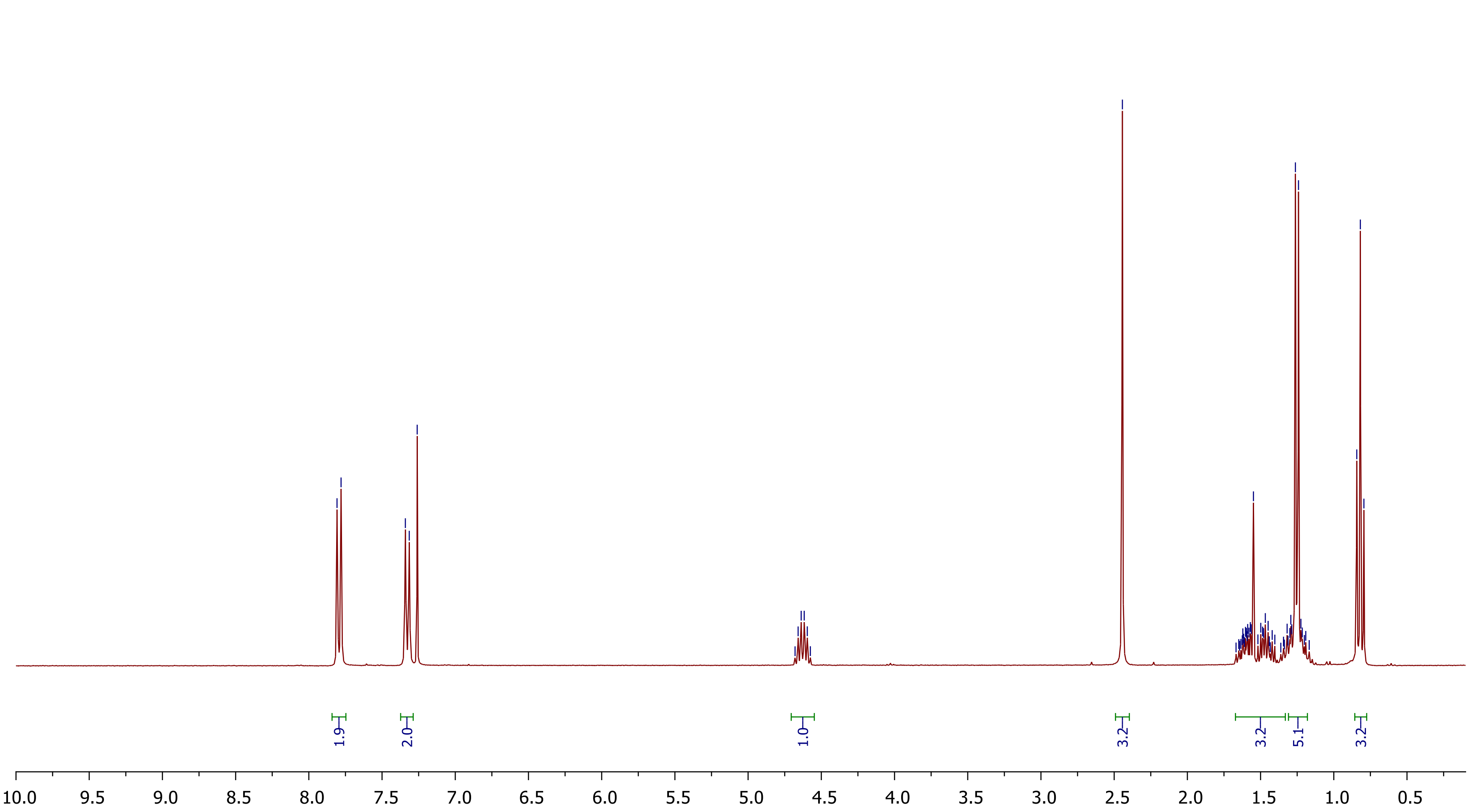

CDCl$_3$, 300 MHz
7.808
7.780
7.341
7.314
7.260
4.679
4.658
4.637
4.617
4.596
4.575
2.444
1.588
1.570
1.549
1.499
1.468
1.448
1.319
1.293
1.262
1.241
1.225
0.843
0.818
0.794
1.9
2.0
1.0
3.2
3.2
5.1
3.2
10.0
9.5
9.0
8.5
8.0
7.5
7.0
6.5
6.0
5.5
5.0
4.5
4.0
3.5
3.0
2.5
2.0
1.5
1.0
0.5

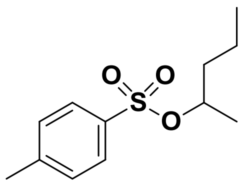

4j

CDCl$_3$, 75 MHz

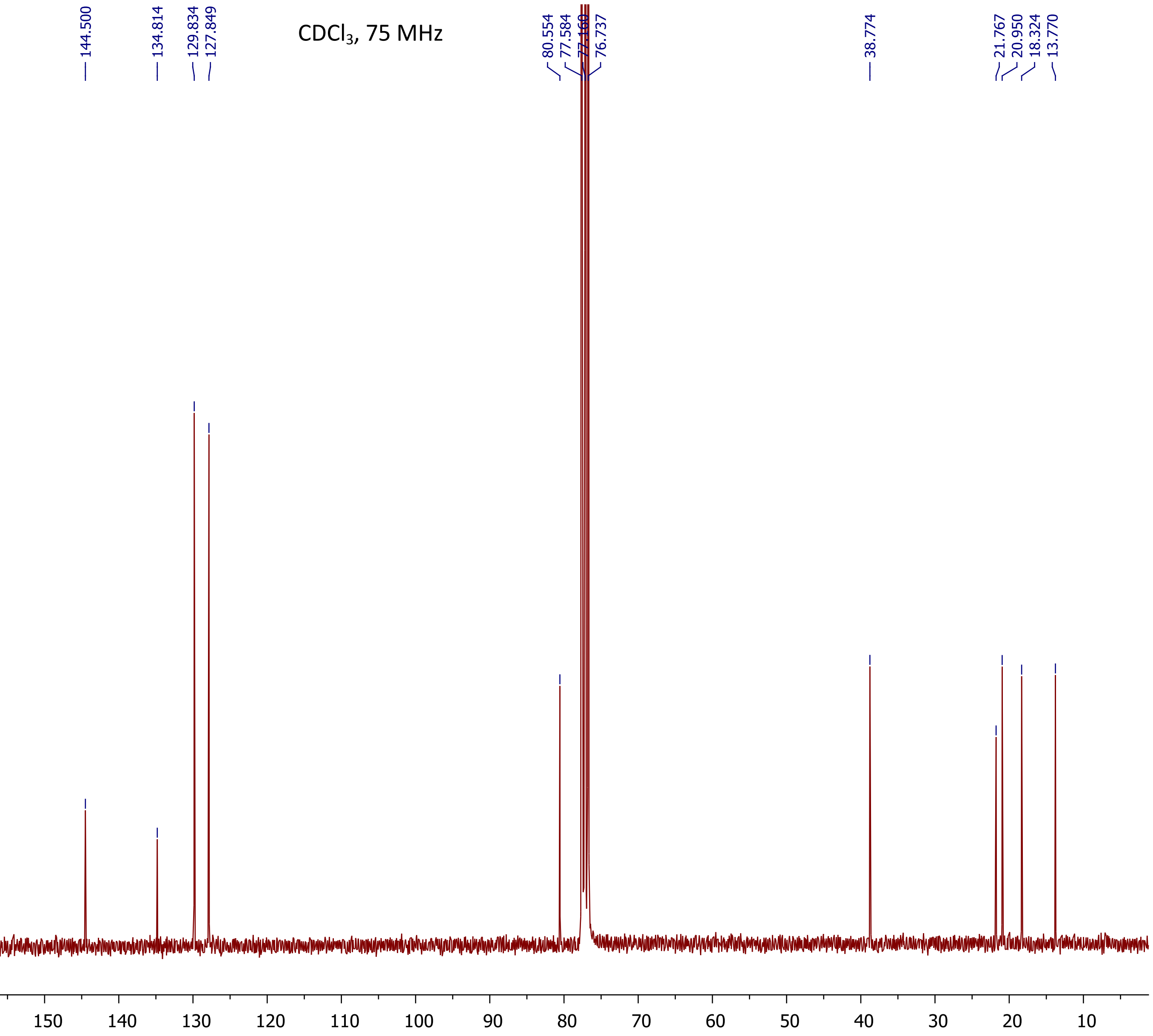

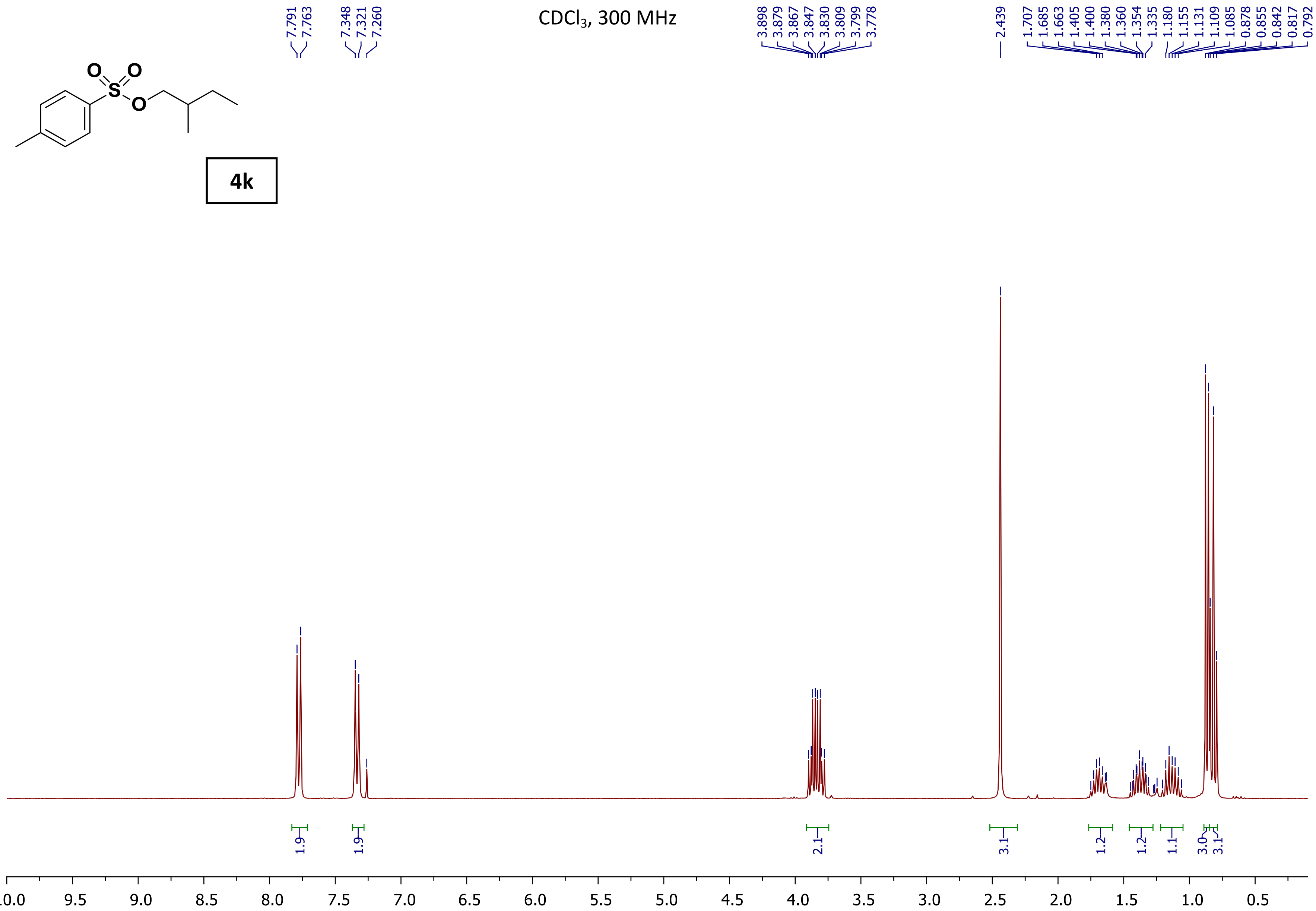

4k
CDCl$_3$, 300 MHz
7.791
7.763
7.348
7.321
7.260
3.898
3.879
3.867
3.847
3.830
3.809
3.799
3.778
2.439
1.707
1.685
1.663
1.405
1.400
1.380
1.360
1.354
1.335
1.180
1.155
1.131
1.109
1.085
0.878
0.855
0.842
0.817
0.792
1.9
1.9
2.1
3.1
1.2
1.2
1.1
3.0
3.1
10.0 9.5 9.0 8.5 8.0 7.5 7.0 6.5 6.0 5.5 5.0 4.5 4.0 3.5 3.0 2.5 2.0 1.5 1.0 0.5

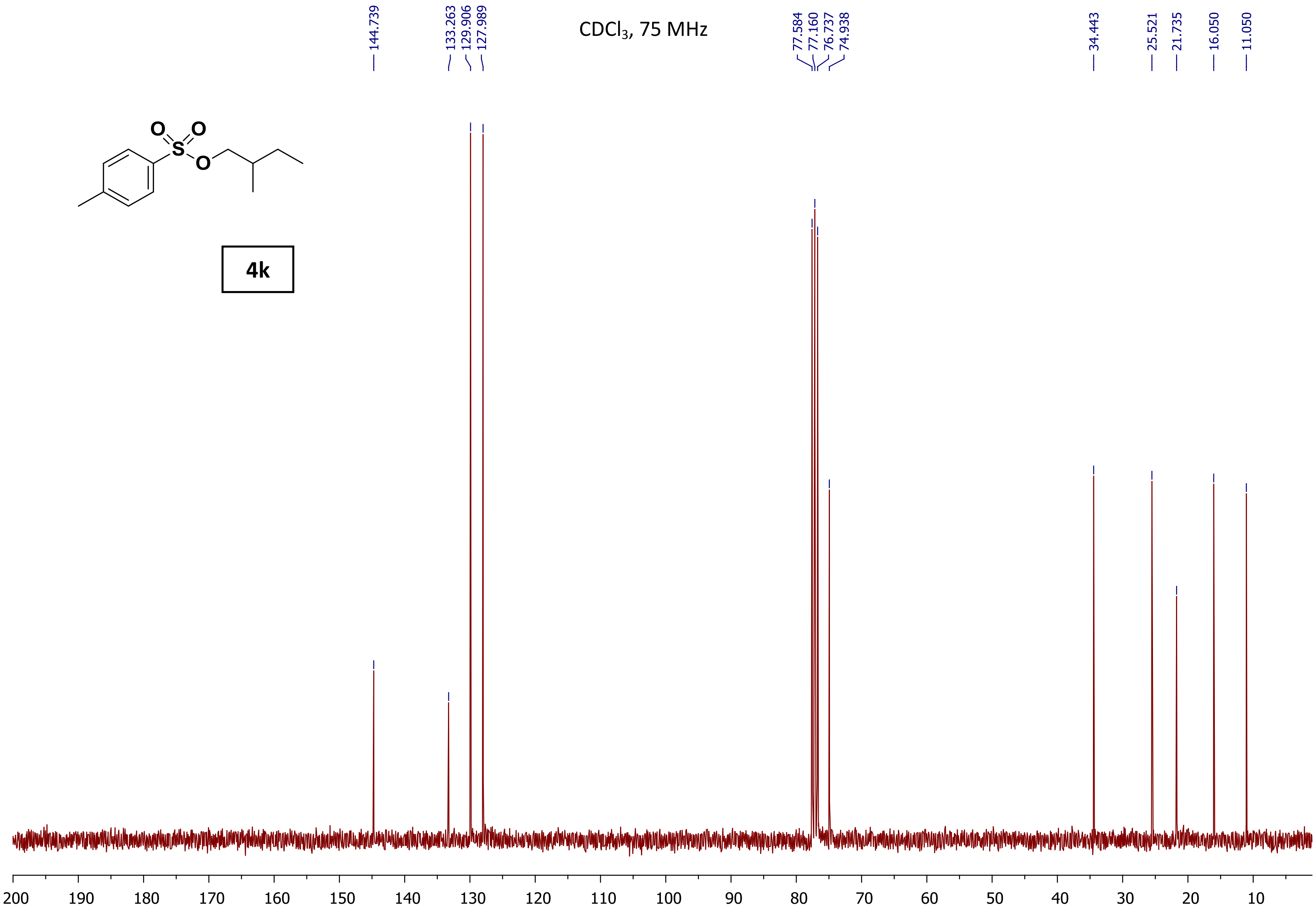

CDCl$_3$, 75 MHz
144.739
133.263
129.906
127.989
77.584
77.160
76.737
74.938
34.443
25.521
21.735
16.050
11.050
4k
200 190 180 170 160 150 140 130 120 110 100 90 80 70 60 50 40 30 20 10

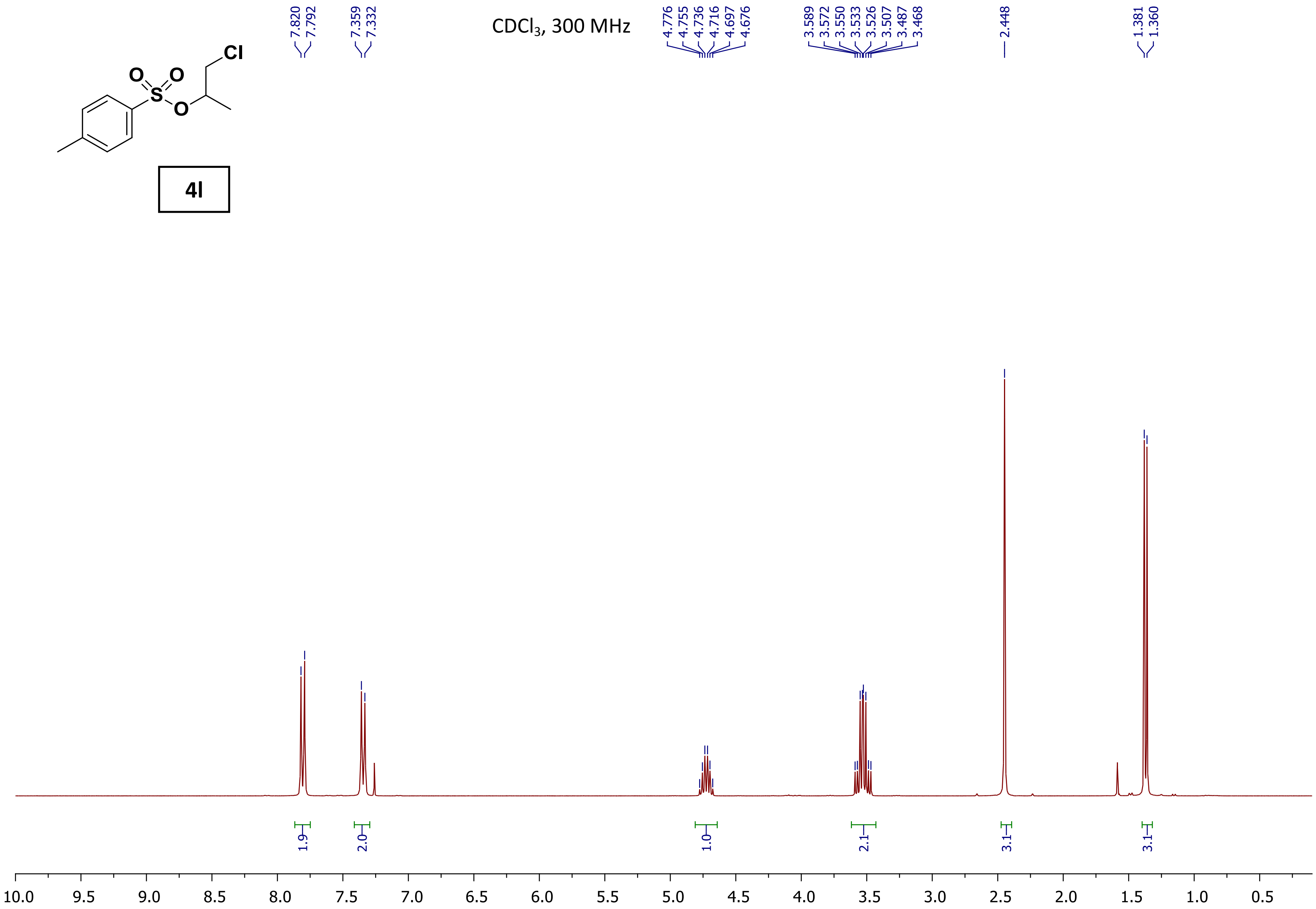

4l
CDCl$_3$, 300 MHz
7.820
7.792
7.359
7.332
4.776
4.755
4.736
4.716
4.697
4.676
3.589
3.572
3.550
3.533
3.526
3.507
3.487
3.468
2.448
1.381
1.360
1.9
2.0
1.0
2.1
3.1
3.1
10.0 9.5 9.0 8.5 8.0 7.5 7.0 6.5 6.0 5.5 5.0 4.5 4.0 3.5 3.0 2.5 2.0 1.5 1.0 0.5

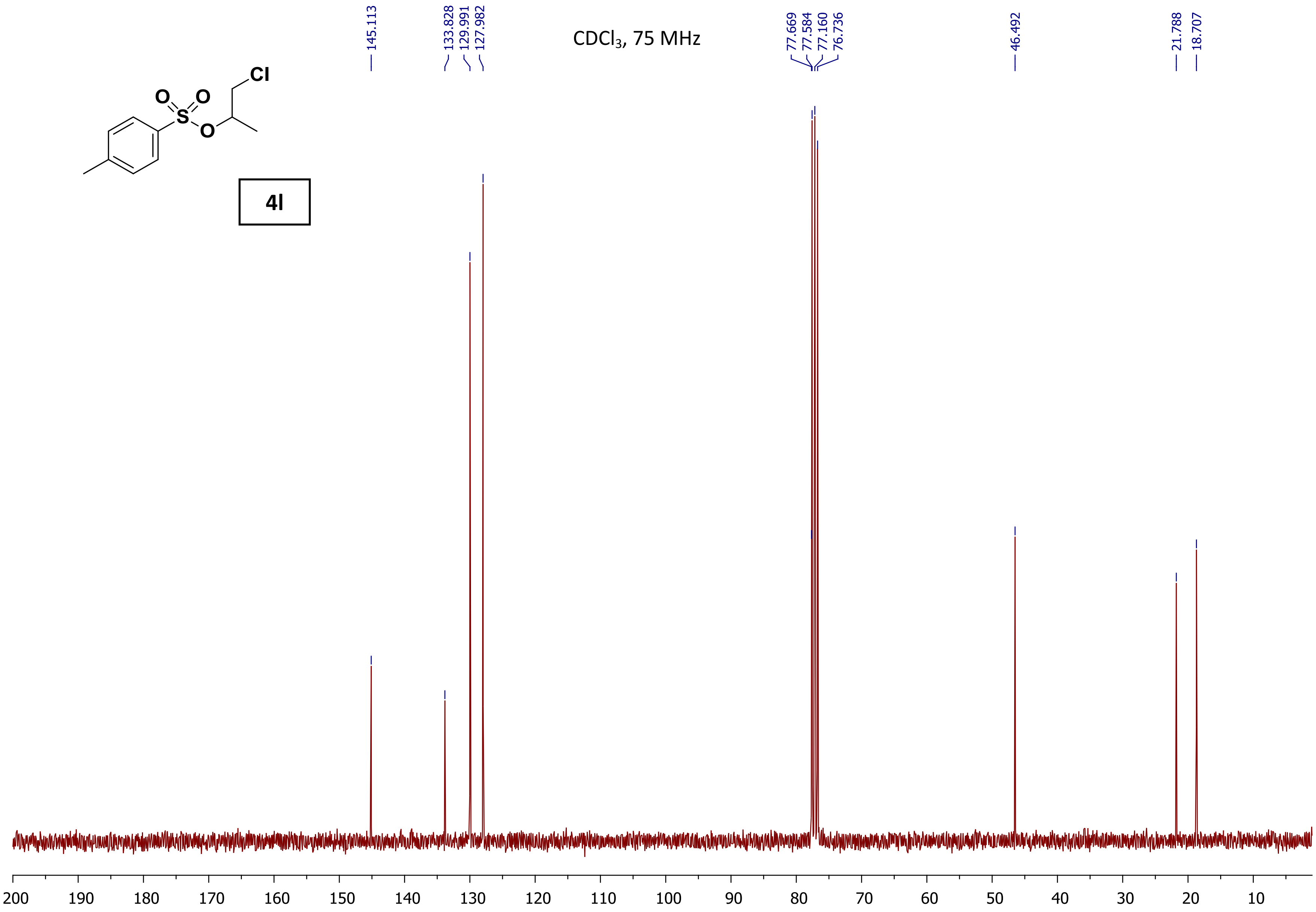
4l
CDCl$_3$, 75 MHz
145.113
133.828
129.991
127.982
77.669
77.584
77.160
76.736
46.492
21.788
18.707
200 190 180 170 160 150 140 130 120 110 100 90 80 70 60 50 40 30 20 10

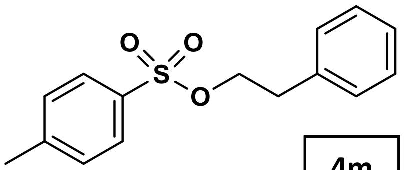

4m

CDCl$_3$, 300 MHz

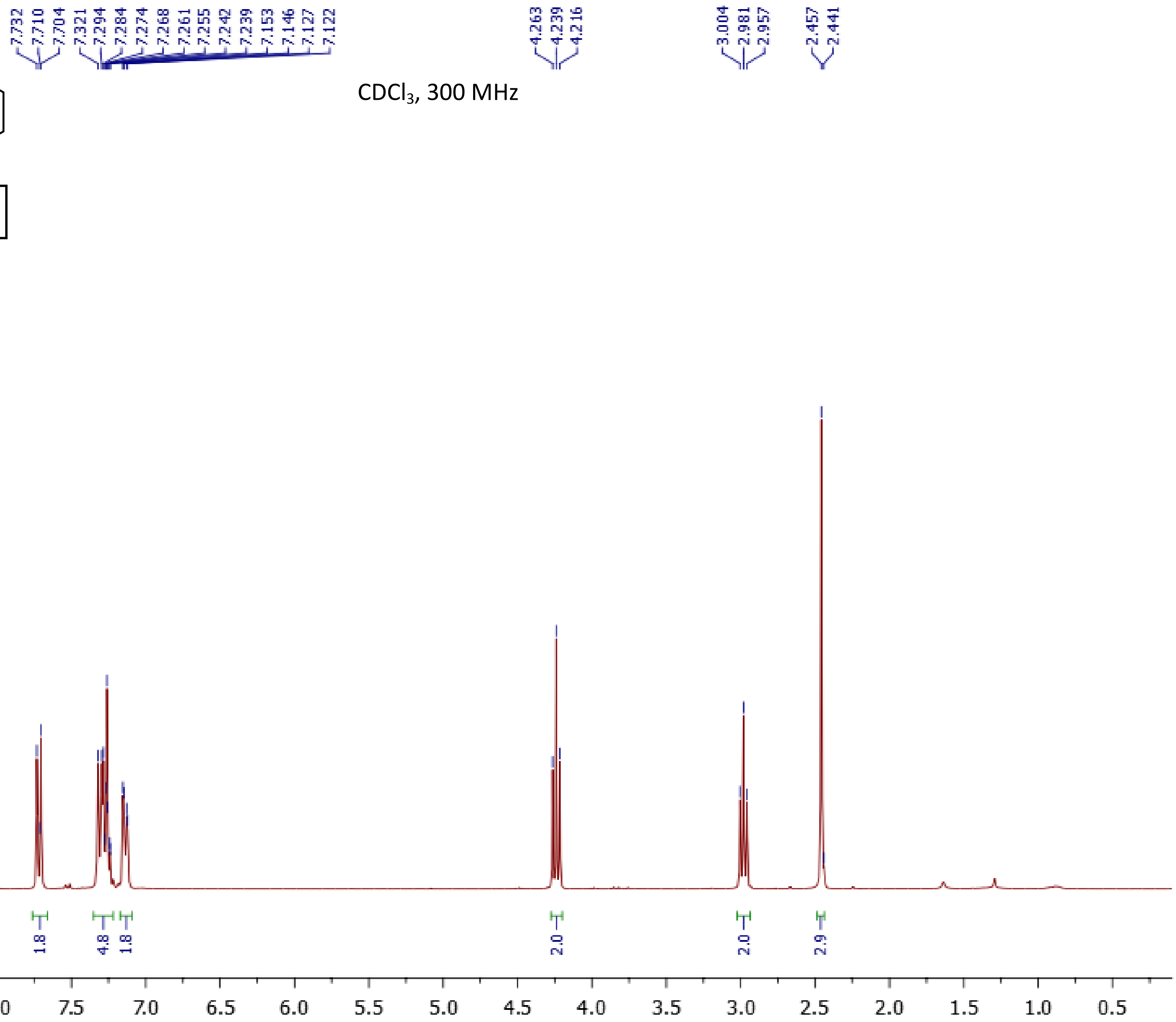

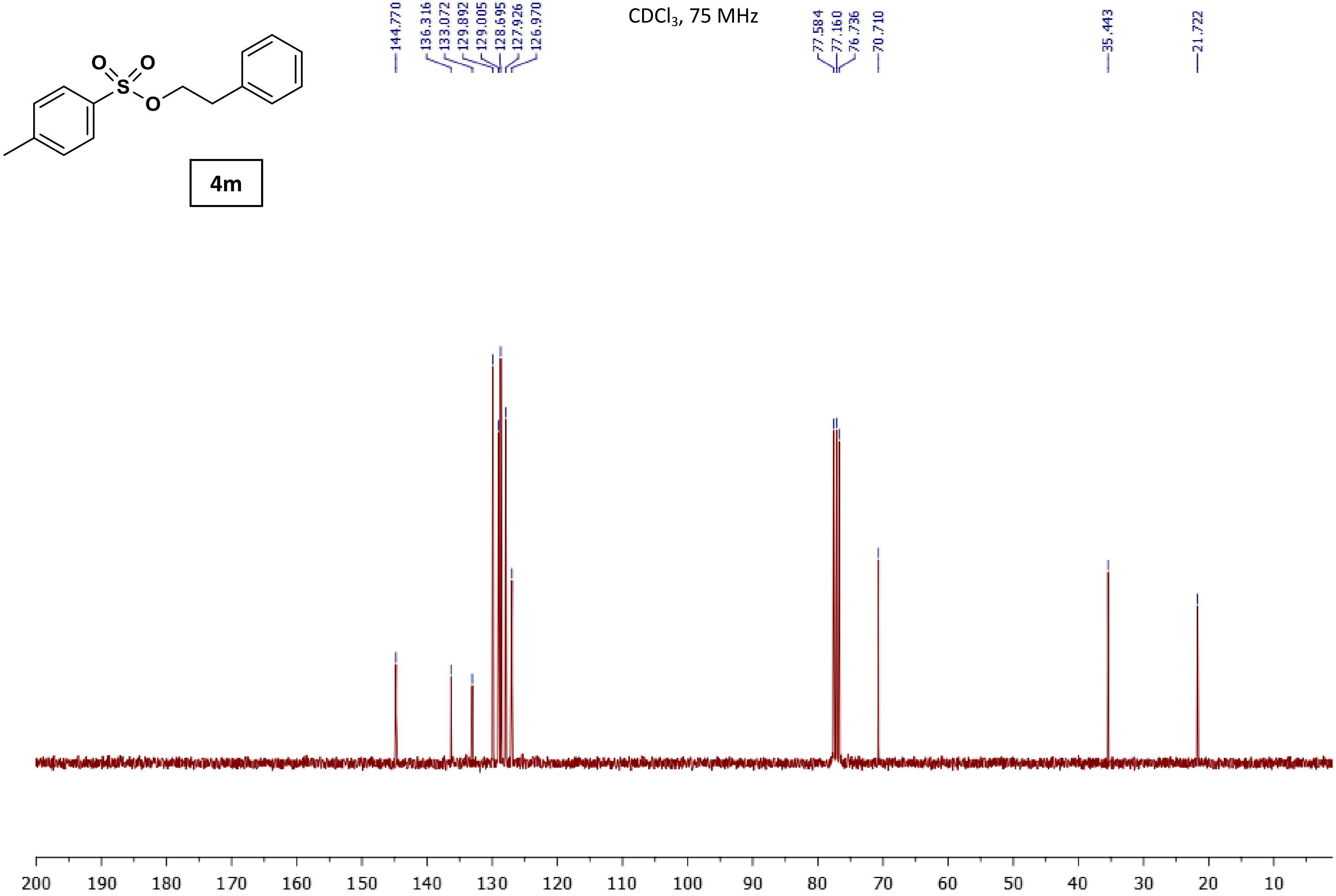

4m
144.770
136.316
133.072
129.892
129.005
128.695
127.926
126.970
CDCl$_3$, 75 MHz
77.584
77.160
76.736
70.710
35.443
21.722
200 190 180 170 160 150 140 130 120 110 100 90 80 70 60 50 40 30 20 10

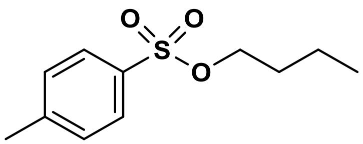

4o

CDCl$_3$, 300 MHz

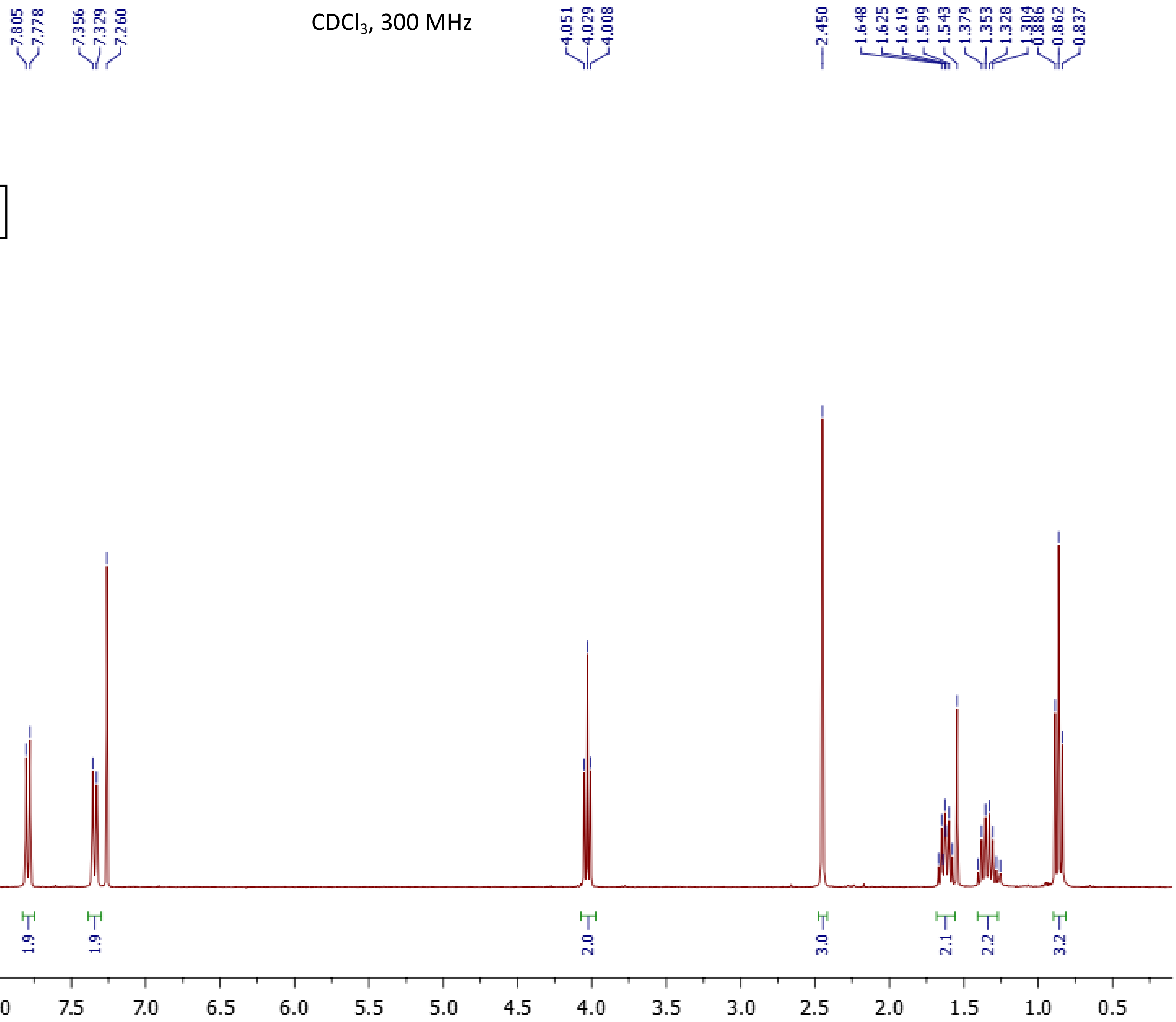

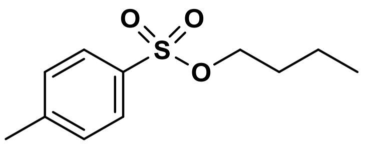

4o

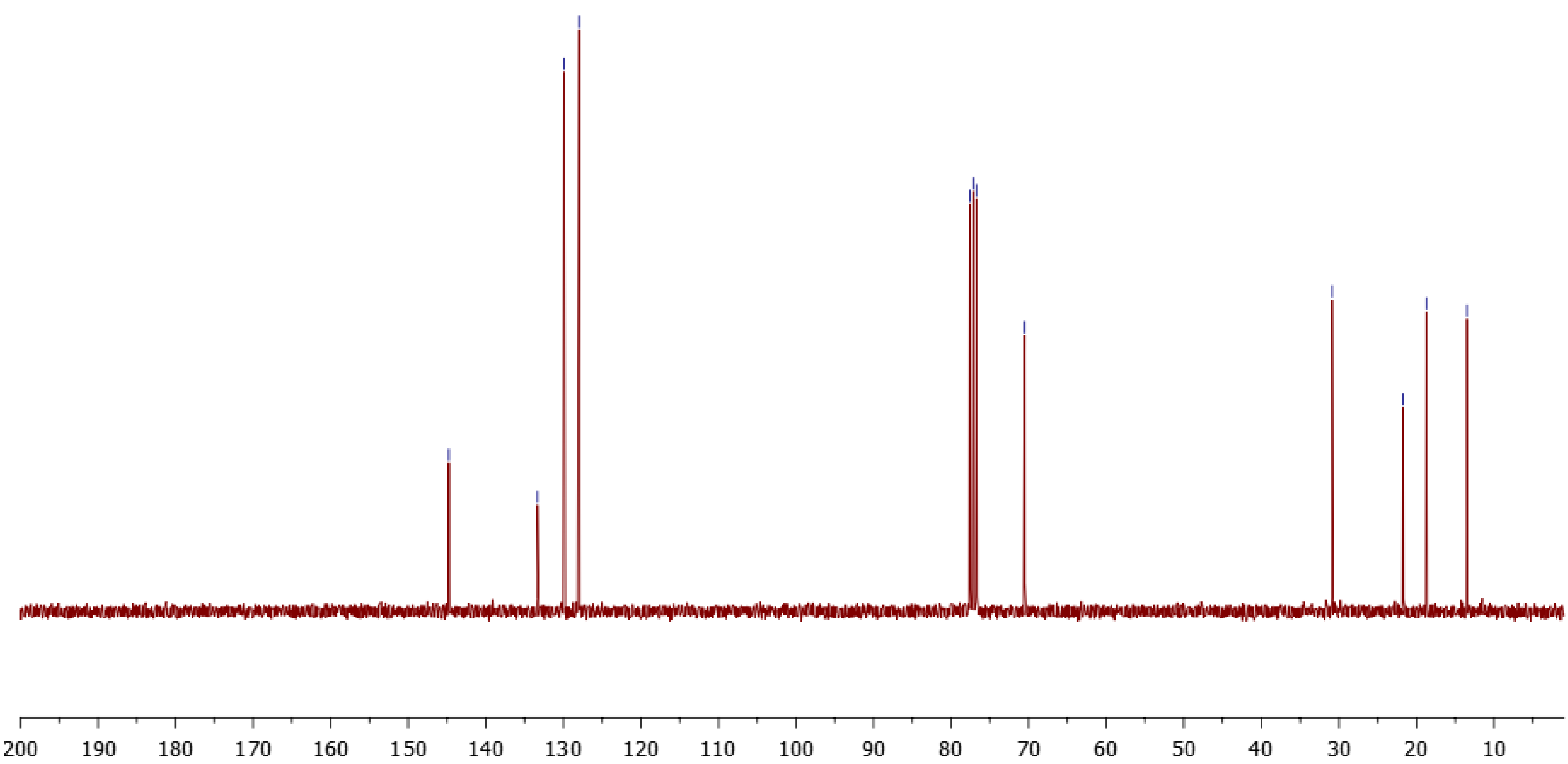

144.752
133.333
129.911
127.969
CDCl3, 75 MHz
77.583
77.160
76.736
70.505
30.882
21.723
18.694
13.473
200 190 180 170 160 150 140 130 120 110 100 90 80 70 60 50 40 30 20 10

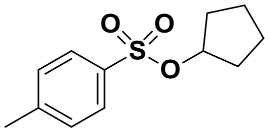

4p

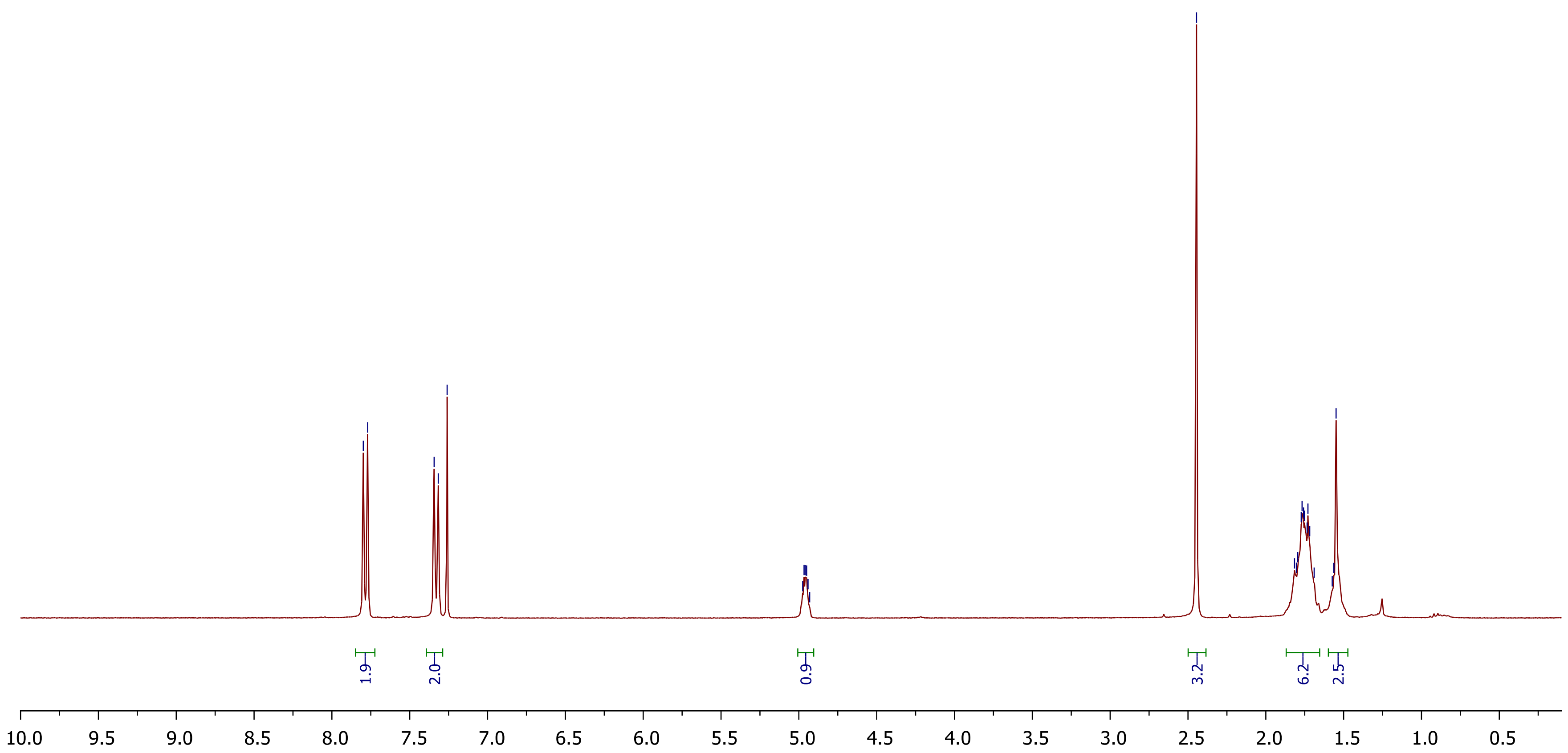
CDCl$_3$, 300 MHz
7.798
7.770
7.343
7.316
7.260
4.975
4.967
4.959
4.950
4.941
4.930
2.445
1.816
1.803
1.795
1.772
1.767
1.757
1.752
1.735
1.729
1.718
1.689
1.573
1.563
1.549
1.9
2.0
0.9
3.2
6.2
2.5
10.0
9.5
9.0
8.5
8.0
7.5
7.0
6.5
6.0
5.5
5.0
4.5
4.0
3.5
3.0
2.5
2.0
1.5
1.0
0.5

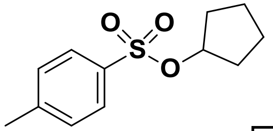

4p

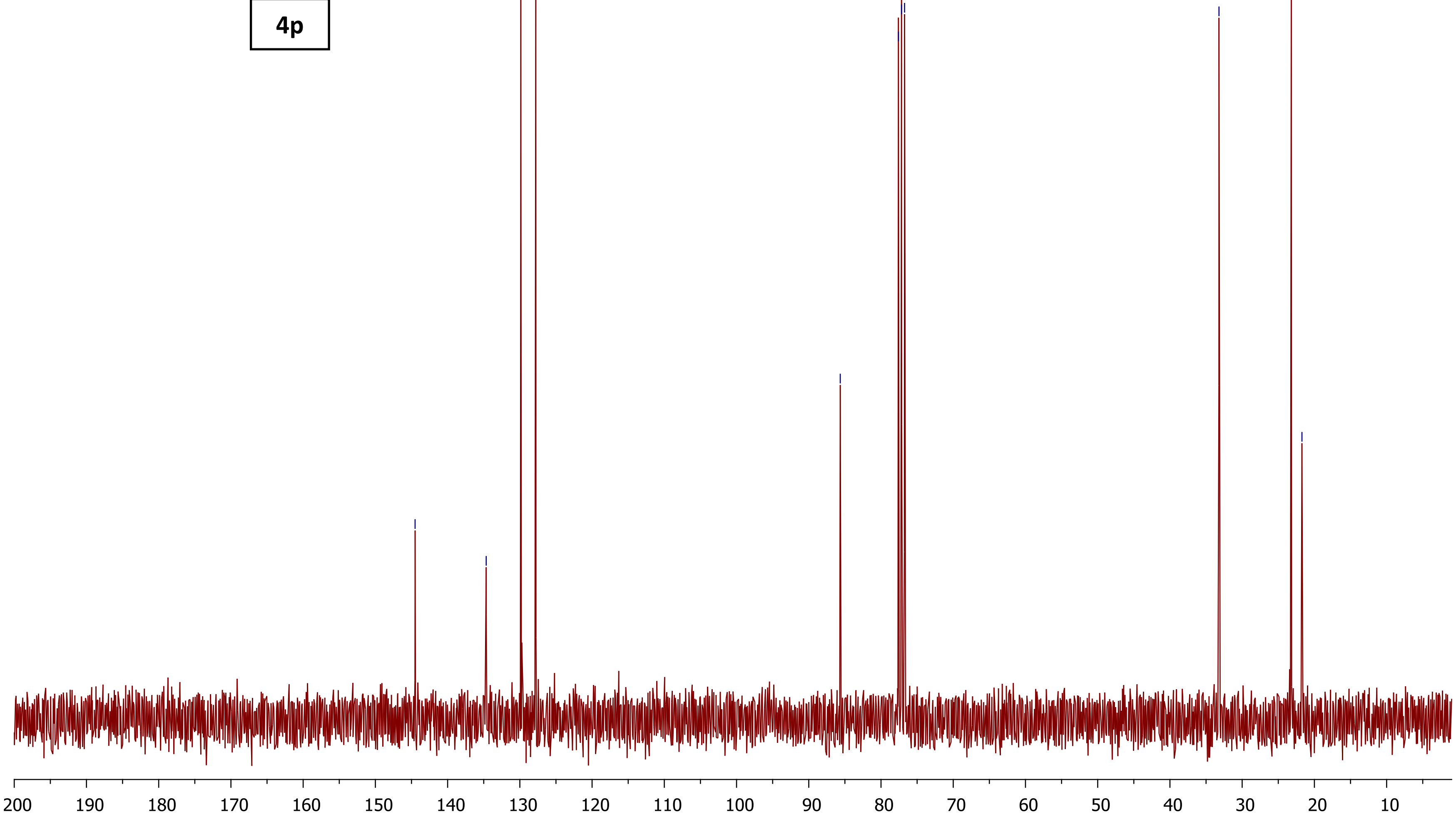
CDCl$_3$, 75 MHz
144.503
134.660
129.865
127.786
85.644
77.583
77.160
76.736
33.210
23.205
21.723
200 190 180 170 160 150 140 130 120 110 100 90 80 70 60 50 40 30 20 10

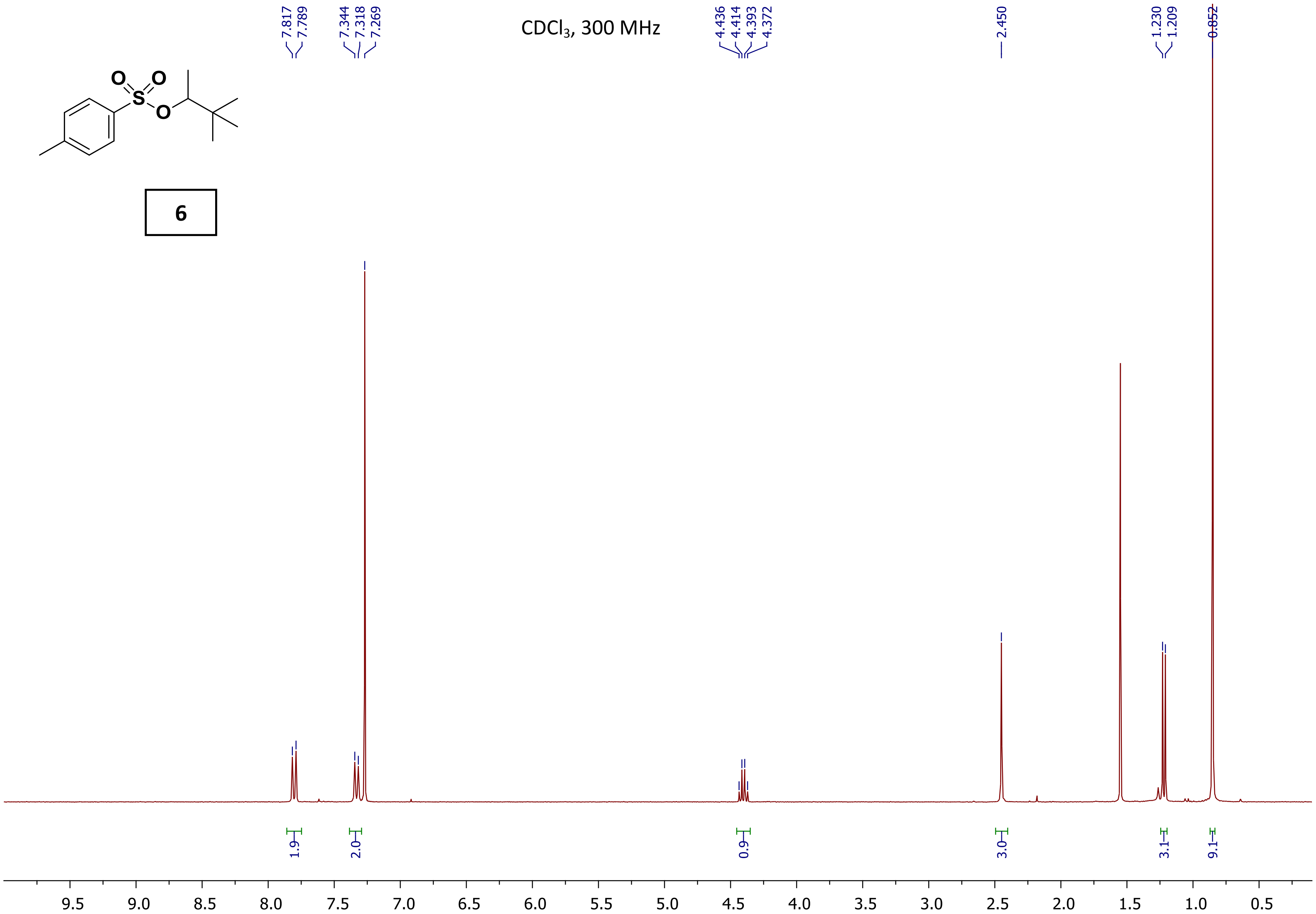
CDCl$_3$, 300 MHz
7.817
7.789
7.344
7.318
7.269
4.436
4.414
4.393
4.372
2.450
1.230
1.209
0.852
1.9
2.0
0.9
3.0
3.1
9.1
9.5
9.0
8.5
8.0
7.5
7.0
6.5
6.0
5.5
5.0
4.5
4.0
3.5
3.0
2.5
2.0
1.5
1.0
0.5

6

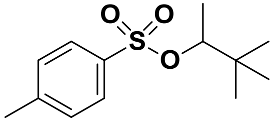
O
O
S
O

6

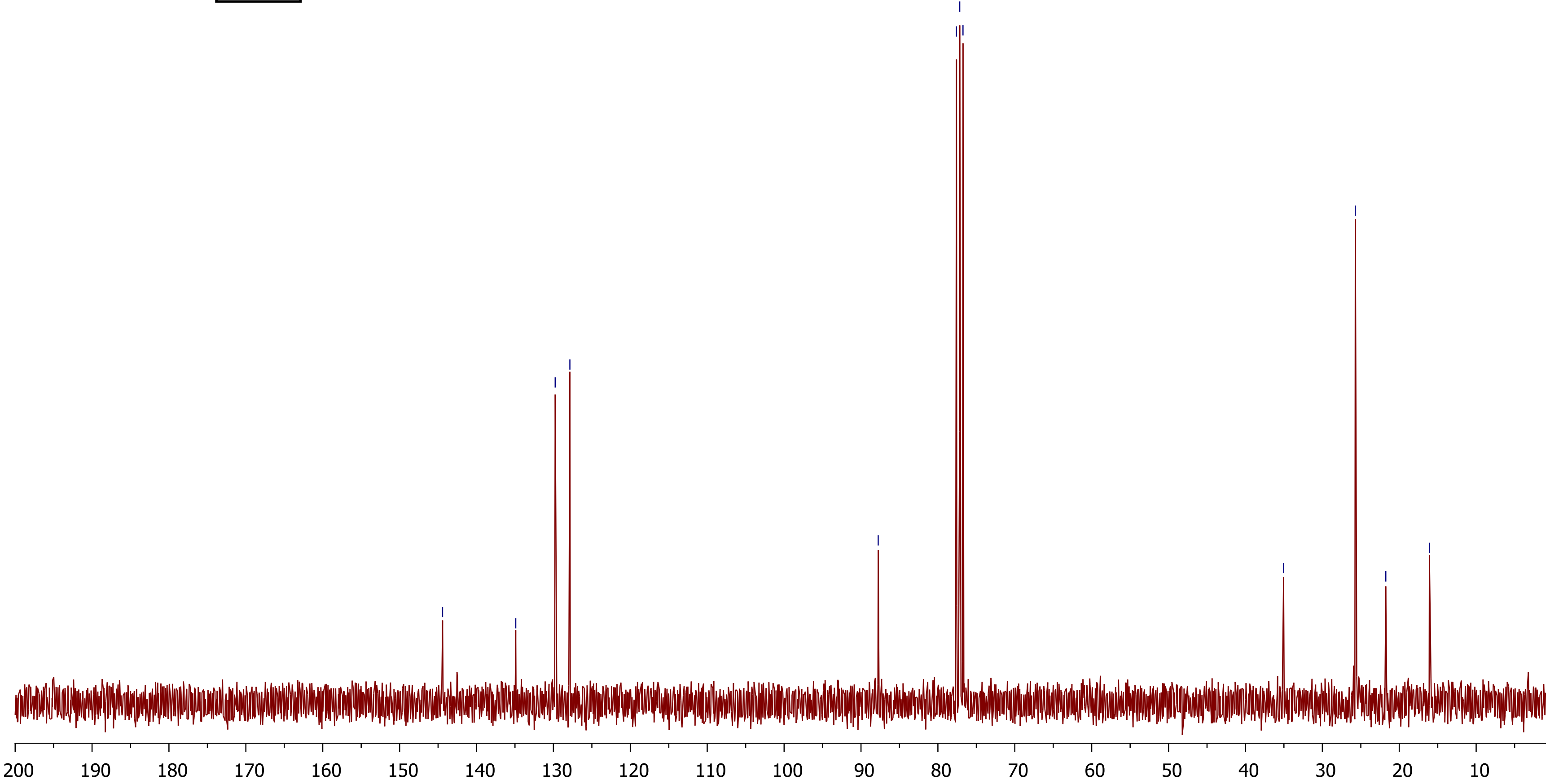
144.416
134.901
129.771
127.860
CDCl$_3$, 75 MHz
87.766
77.584
77.160
76.737
35.042
25.712
21.752
16.083
200
190
180
170
160
150
140
130
120
110
100
90
80
70
60
50
40
30
20
10

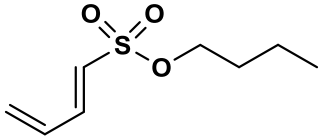
O
O
S
O

8a

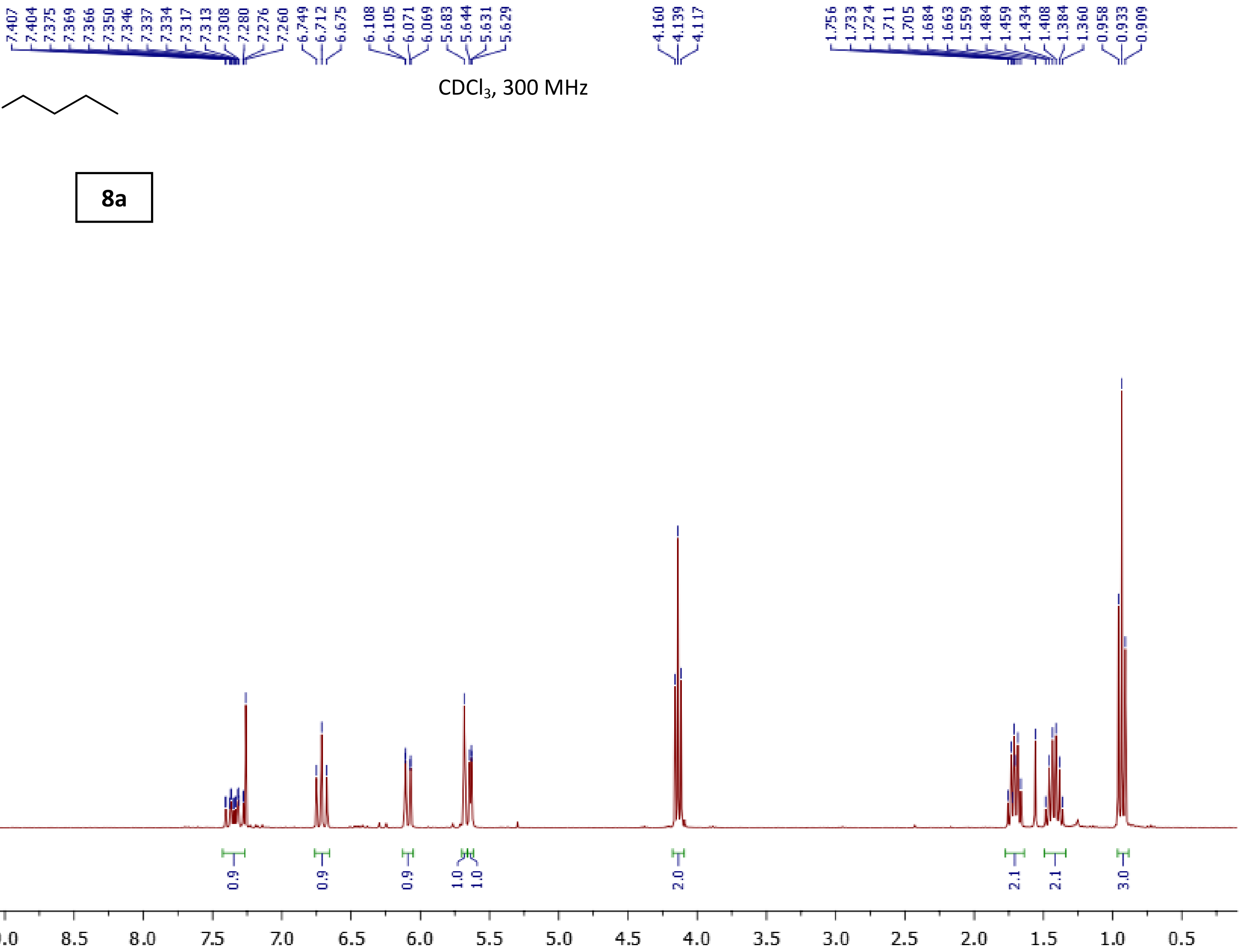
7.407
7.404
7.375
7.369
7.366
7.350
7.346
7.337
7.334
7.317
7.313
7.308
7.280
7.276
7.260
6.749
6.712
6.675
6.108
6.105
6.071
6.069
5.683
5.644
5.631
5.629
4.160
4.139
4.117
1.756
1.733
1.724
1.711
1.705
1.684
1.663
1.559
1.484
1.459
1.434
1.408
1.384
1.360
0.958
0.933
0.909
CDCl3, 300 MHz
0.9
0.9
0.9
1.0
1.0
2.0
2.1
2.1
3.0
10.0
9.5
9.0
8.5
8.0
7.5
7.0
6.5
6.0
5.5
5.0
4.5
4.0
3.5
3.0
2.5
2.0
1.5
1.0
0.5

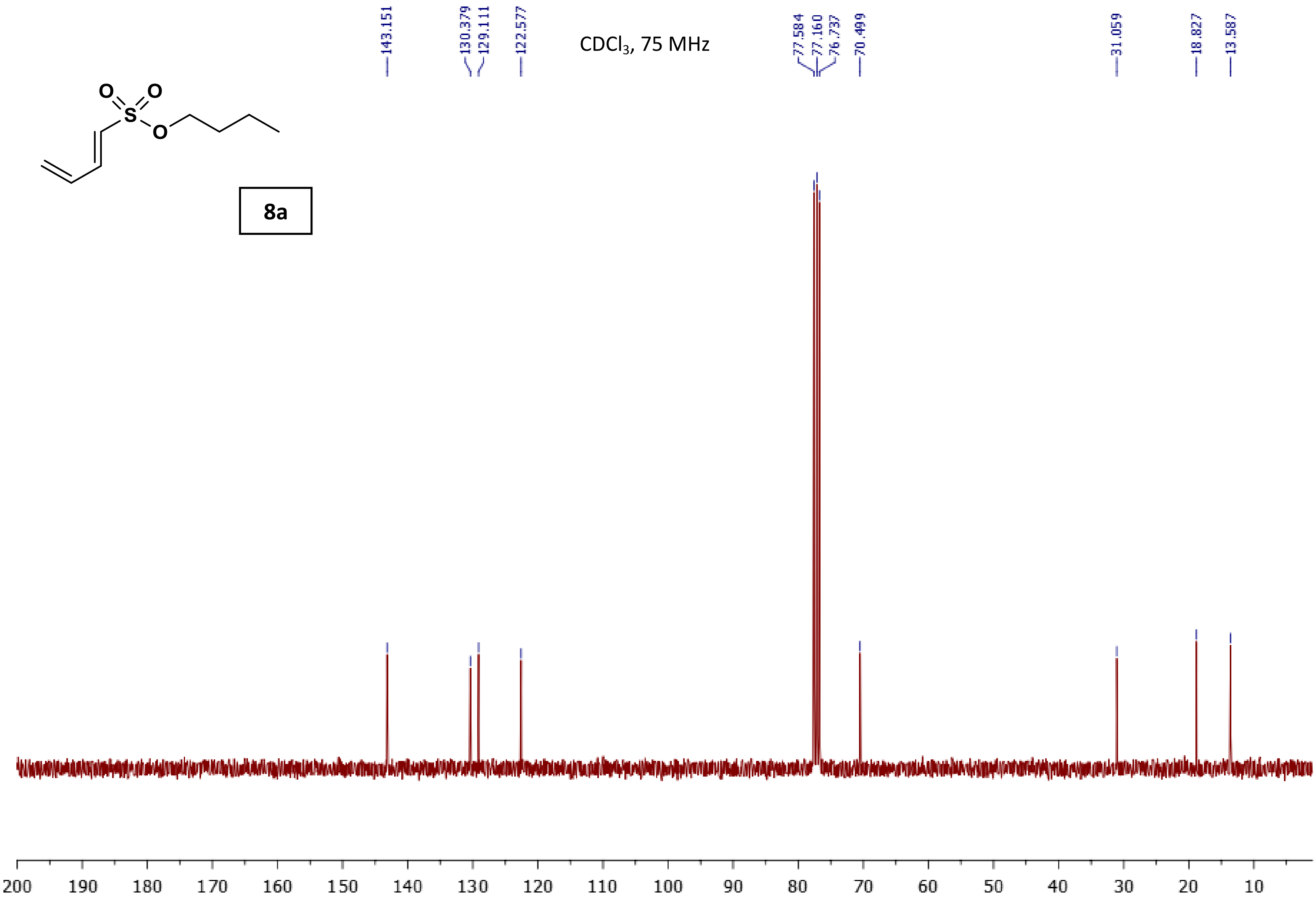
143.151
130.379
129.111
122.577
CDCl$_3$, 75 MHz
77.584
77.160
76.737
70.499
31.059
18.827
13.587
200 190 180 170 160 150 140 130 120 110 100 90 80 70 60 50 40 30 20 10

8a

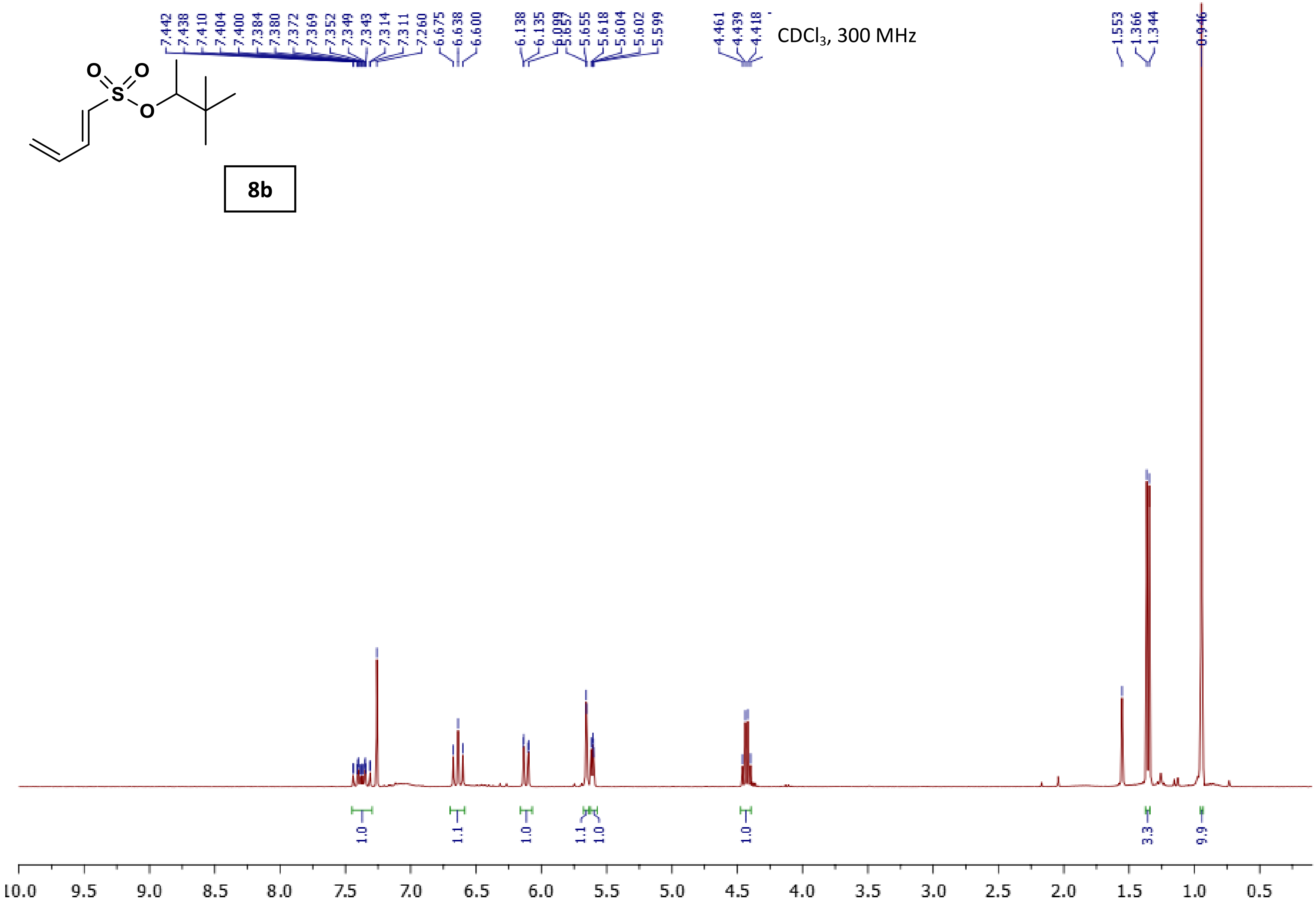

7.442
7.438
7.410
7.404
7.400
7.384
7.380
7.372
7.369
7.352
7.349
7.343
7.314
7.311
7.260
6.675
6.638
6.600
6.138
6.135
6.099
5.657
5.655
5.618
5.604
5.602
5.599
4.461
4.439
4.418
8b
CDCl$_3$, 300 MHz
1.553
1.366
1.344
0.946
1.0
1.1
1.0
1.1
1.0
1.0
3.3
9.9
10.0 9.5 9.0 8.5 8.0 7.5 7.0 6.5 6.0 5.5 5.0 4.5 4.0 3.5 3.0 2.5 2.0 1.5 1.0 0.5

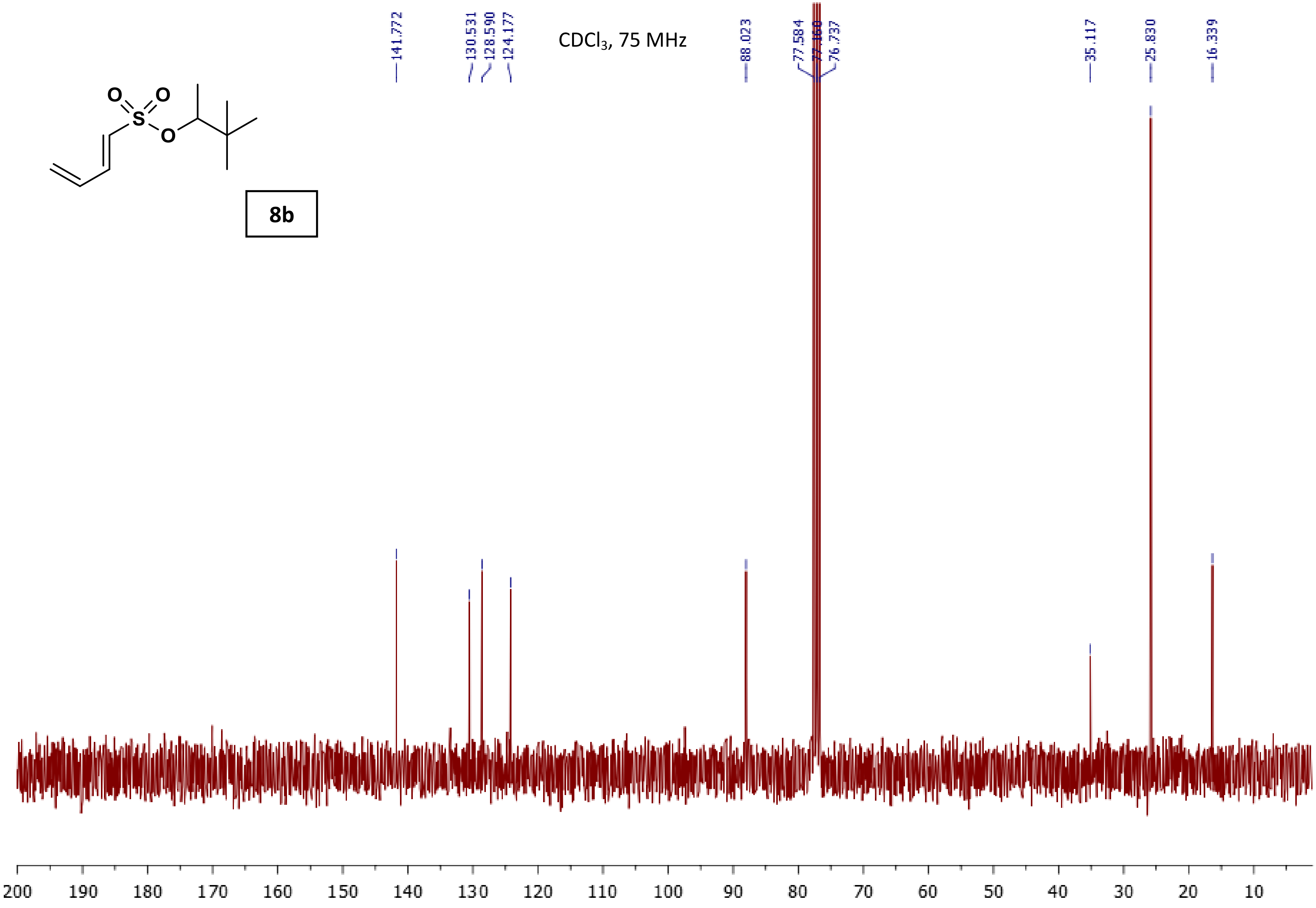

8b
CDCl$_3$, 75 MHz
141.772
130.531
128.590
124.177
88.023
77.584
77.160
76.737
35.117
25.830
16.339
200 190 180 170 160 150 140 130 120 110 100 90 80 70 60 50 40 30 20 10

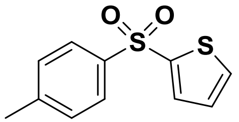

9a

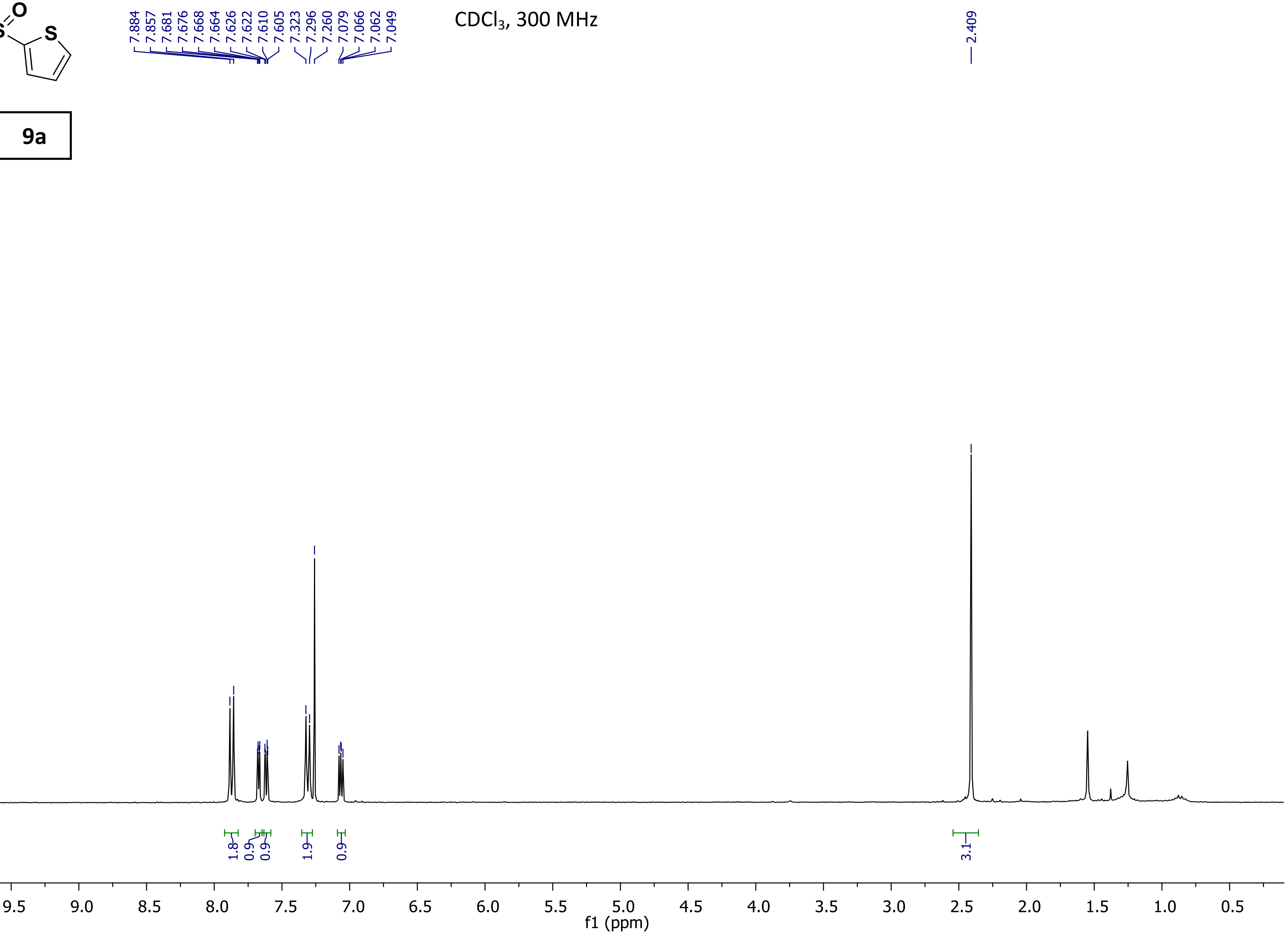
CDCl$_3$, 300 MHz
7.884
7.857
7.681
7.676
7.668
7.664
7.626
7.622
7.610
7.605
7.323
7.296
7.260
7.079
7.066
7.062
7.049
2.409
1.8
0.9
0.9
1.9
0.9
3.1
10.0 9.5 9.0 8.5 8.0 7.5 7.0 6.5 6.0 5.5 5.0 4.5 4.0 3.5 3.0 2.5 2.0 1.5 1.0 0.5
f1 (ppm)

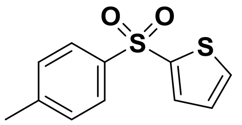

O
O
S
S

9a

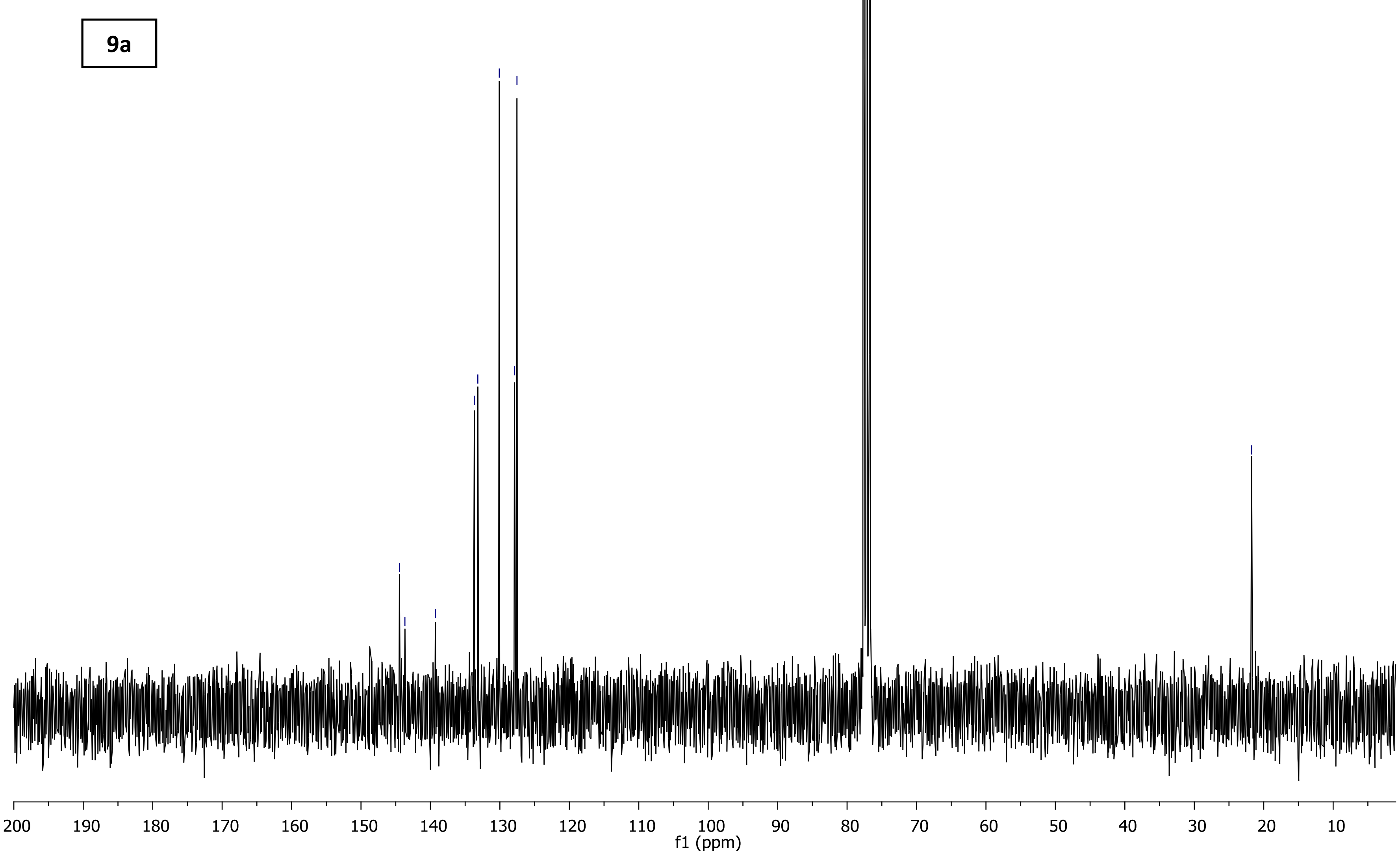

144.467
143.677
139.293
133.679
133.178
130.088
127.892
127.539
CDCl$_3$, 75 MHz
77.584
76.737
21.746
200
190
180
170
160
150
140
130
120
110
100
90
80
70
60
50
40
30
20
10
f1 (ppm)

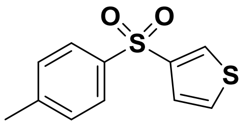
O
O
S
S

9b

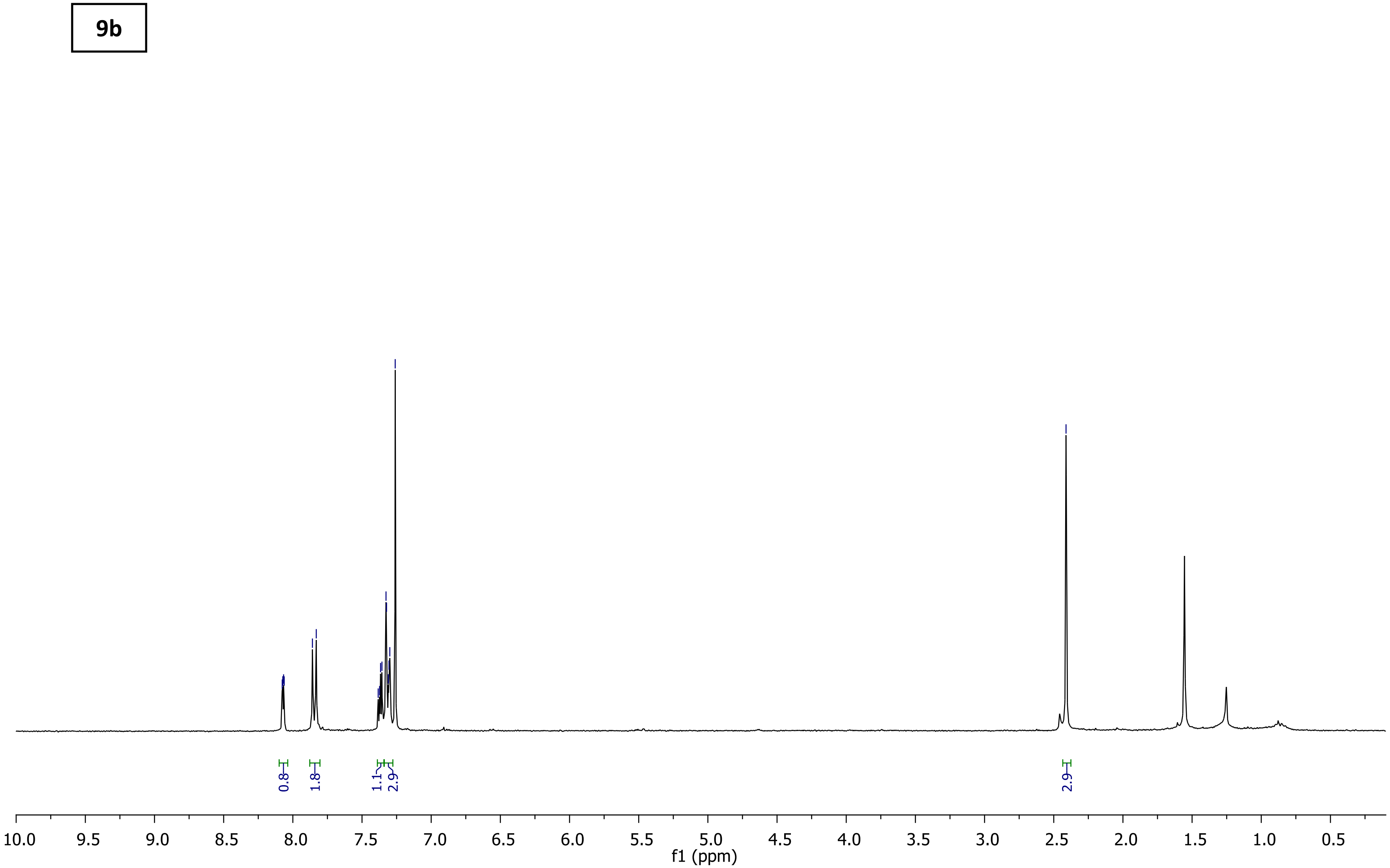
8.077
8.073
8.067
8.062
7.858
7.831
7.383
7.373
7.366
7.356
7.327
7.323
7.310
7.305
7.299
7.260
CDCl3, 300 MHz
2.411
0.8
1.8
1.1
2.9
2.9
10.0
9.5
9.0
8.5
8.0
7.5
7.0
6.5
6.0
5.5
5.0
4.5
4.0
3.5
3.0
2.5
2.0
1.5
1.0
0.5
f1 (ppm)

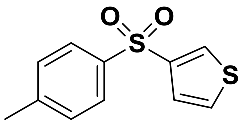

9b

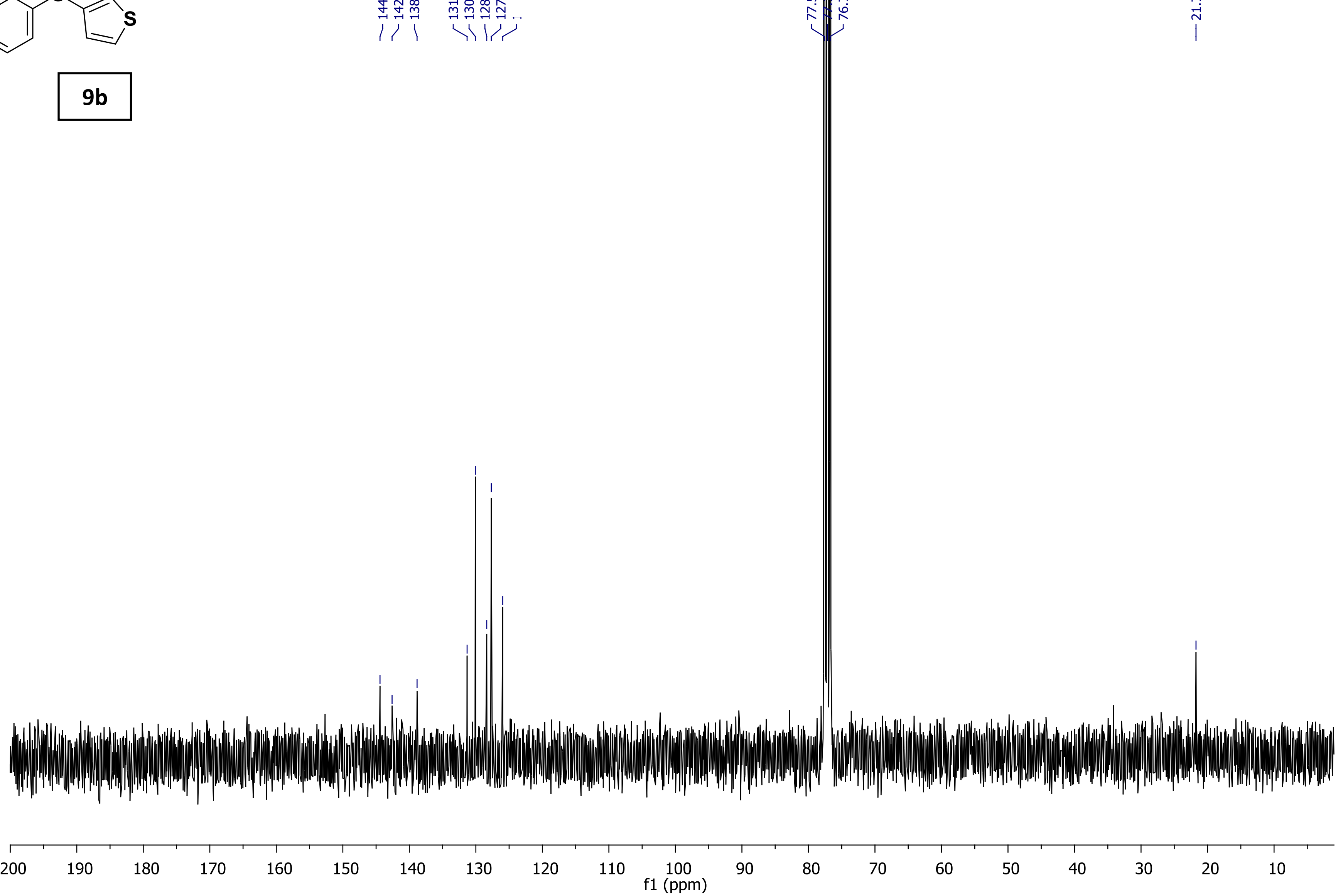
144.411
142.598
138.840
131.320
130.073
128.366
127.681
CDCl$_3$, 75 MHz
77.583
77.160
76.736
21.734
200 190 180 170 160 150 140 130 120 110 100 90 80 70 60 50 40 30 20 10
f1 (ppm)

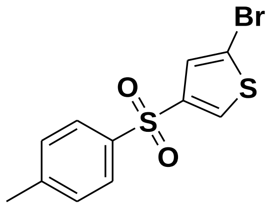

10a

CDCl$_3$, 300 MHz

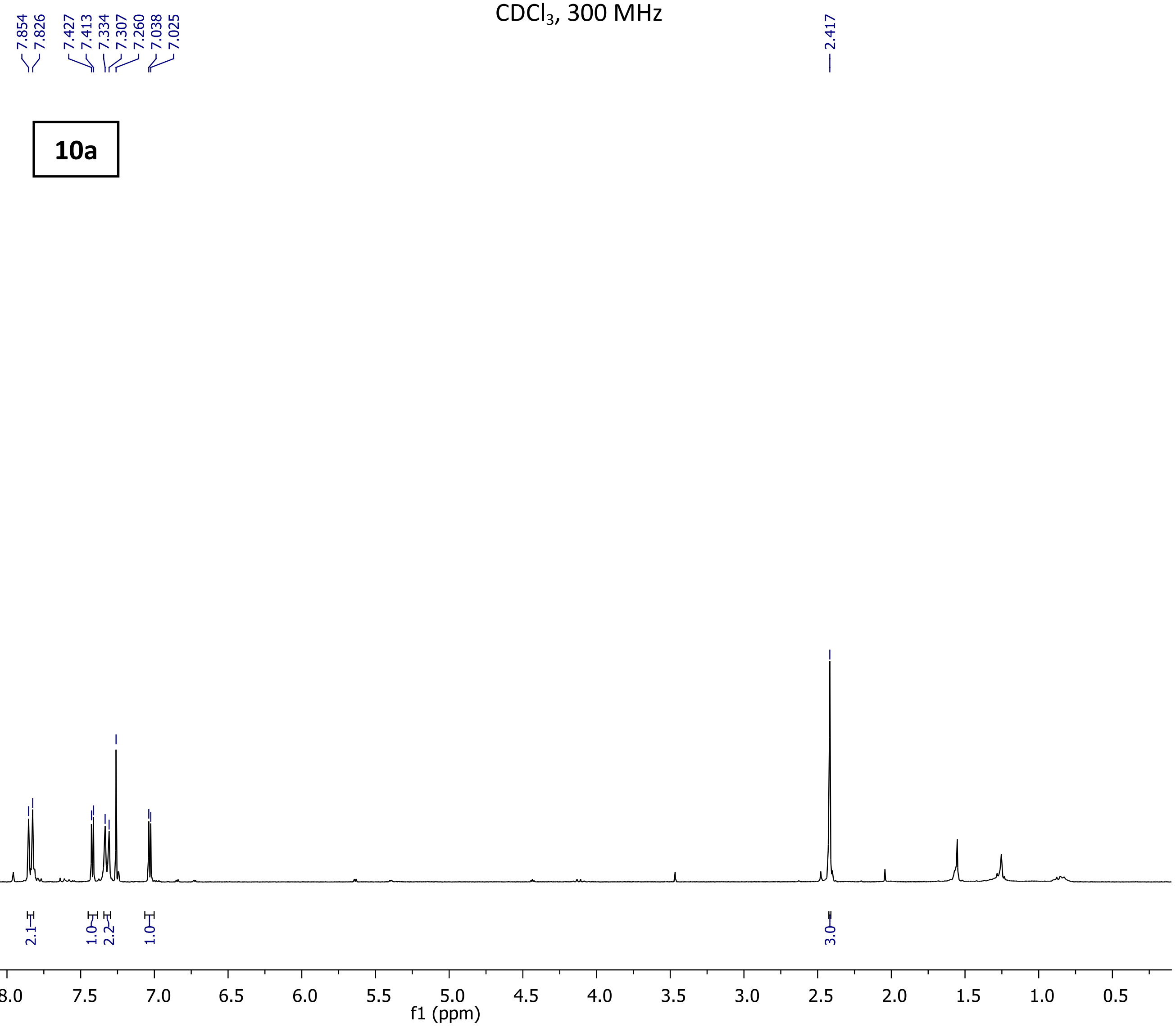

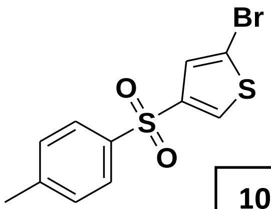

Br
S
O
S
O

10b

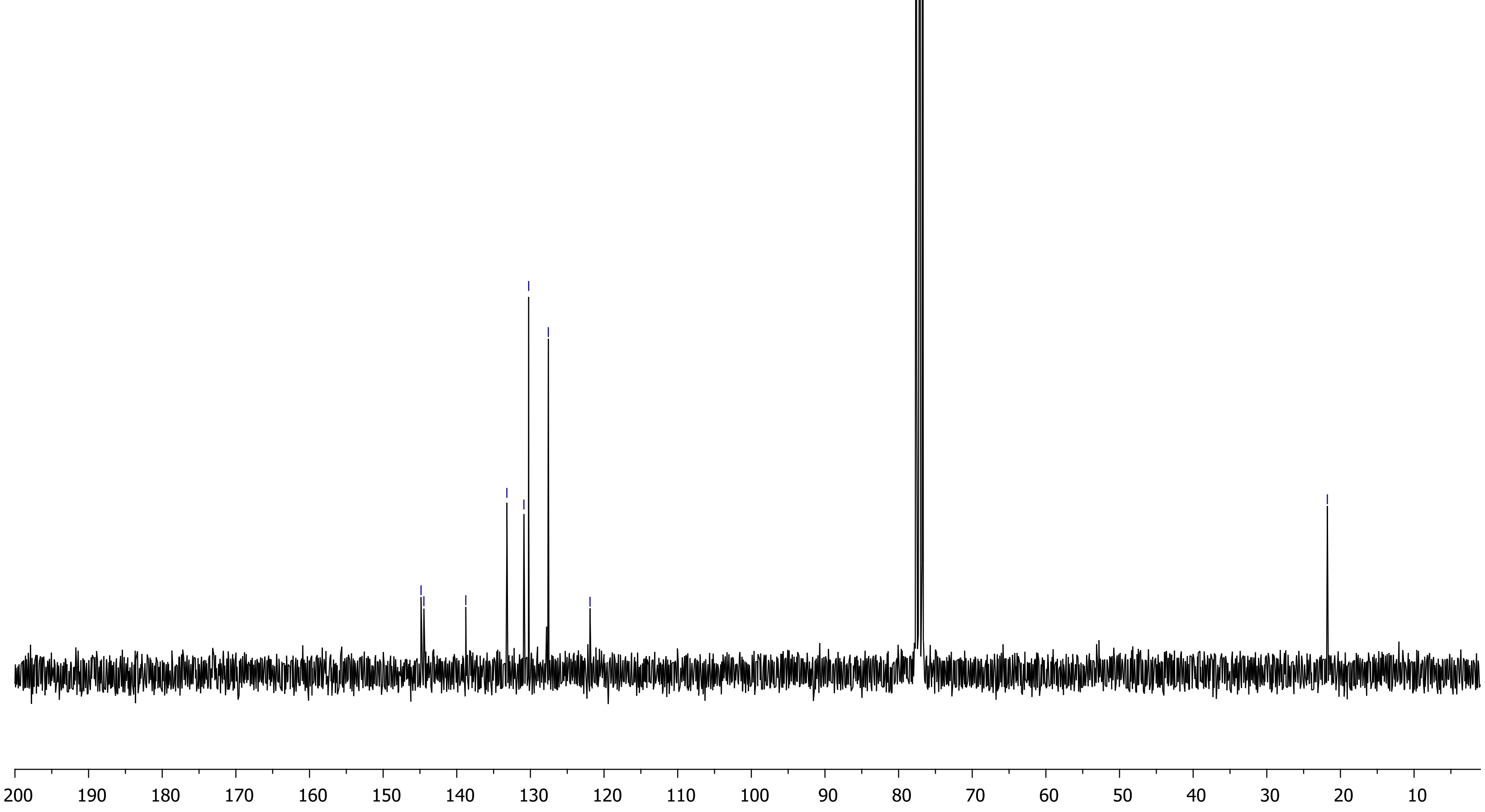

144.852
144.465
138.772
133.197
130.883
130.231
127.568
121.904
CDCl$_3$, 75 MHz
21.774
200 190 180 170 160 150 140 130 120 110 100 90 80 70 60 50 40 30 20 10

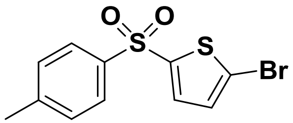

10b

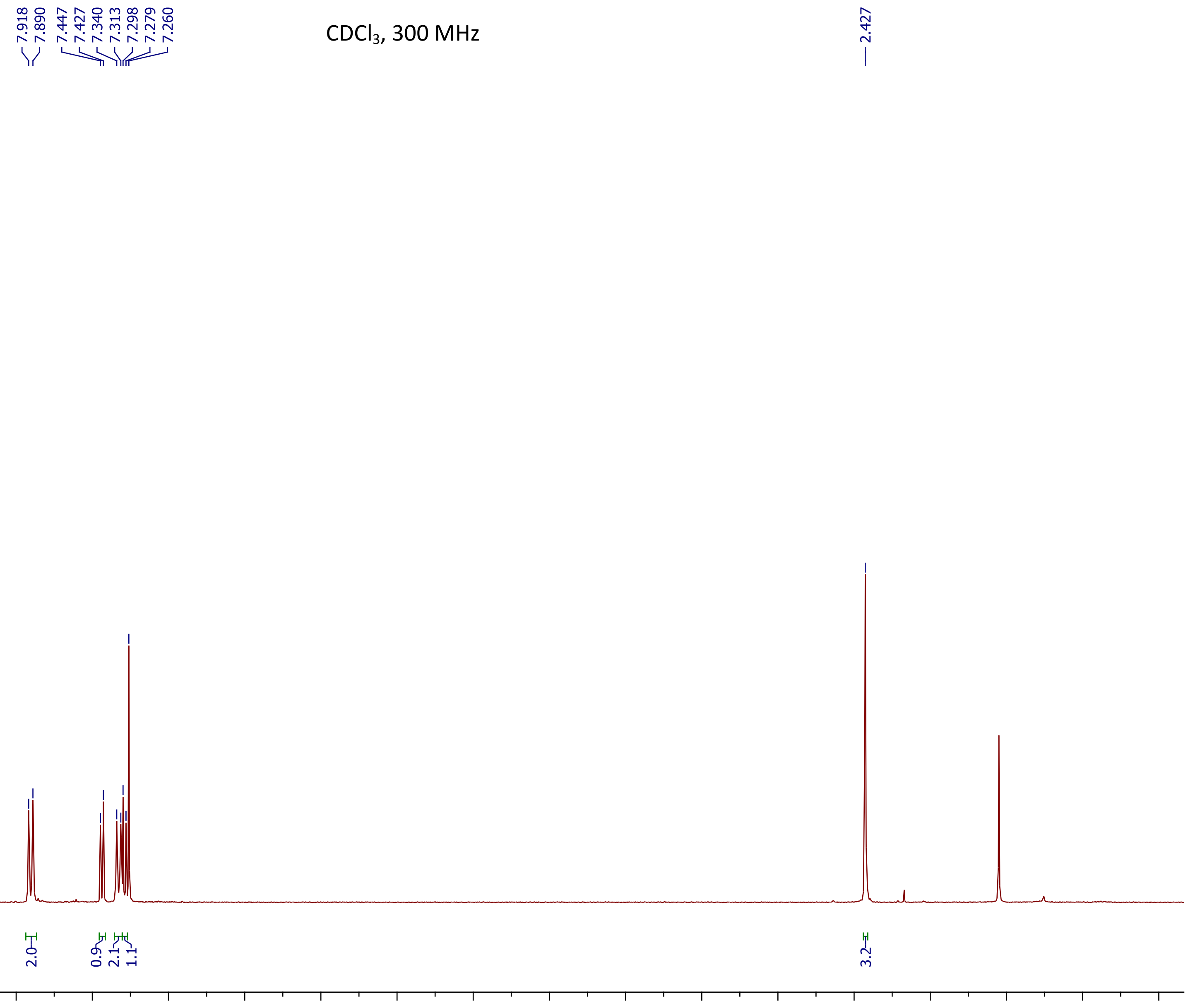
7.918
7.890
7.447
7.427
7.340
7.313
7.298
7.279
7.260
CDCl3, 300 MHz
2.427
2.0
0.9
2.1
1.1
3.2
10.0
9.5
9.0
8.5
8.0
7.5
7.0
6.5
6.0
5.5
5.0
4.5
4.0
3.5
3.0
2.5
2.0
1.5
1.0
0.5

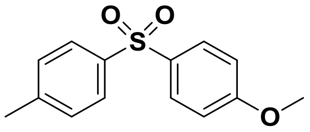

11b

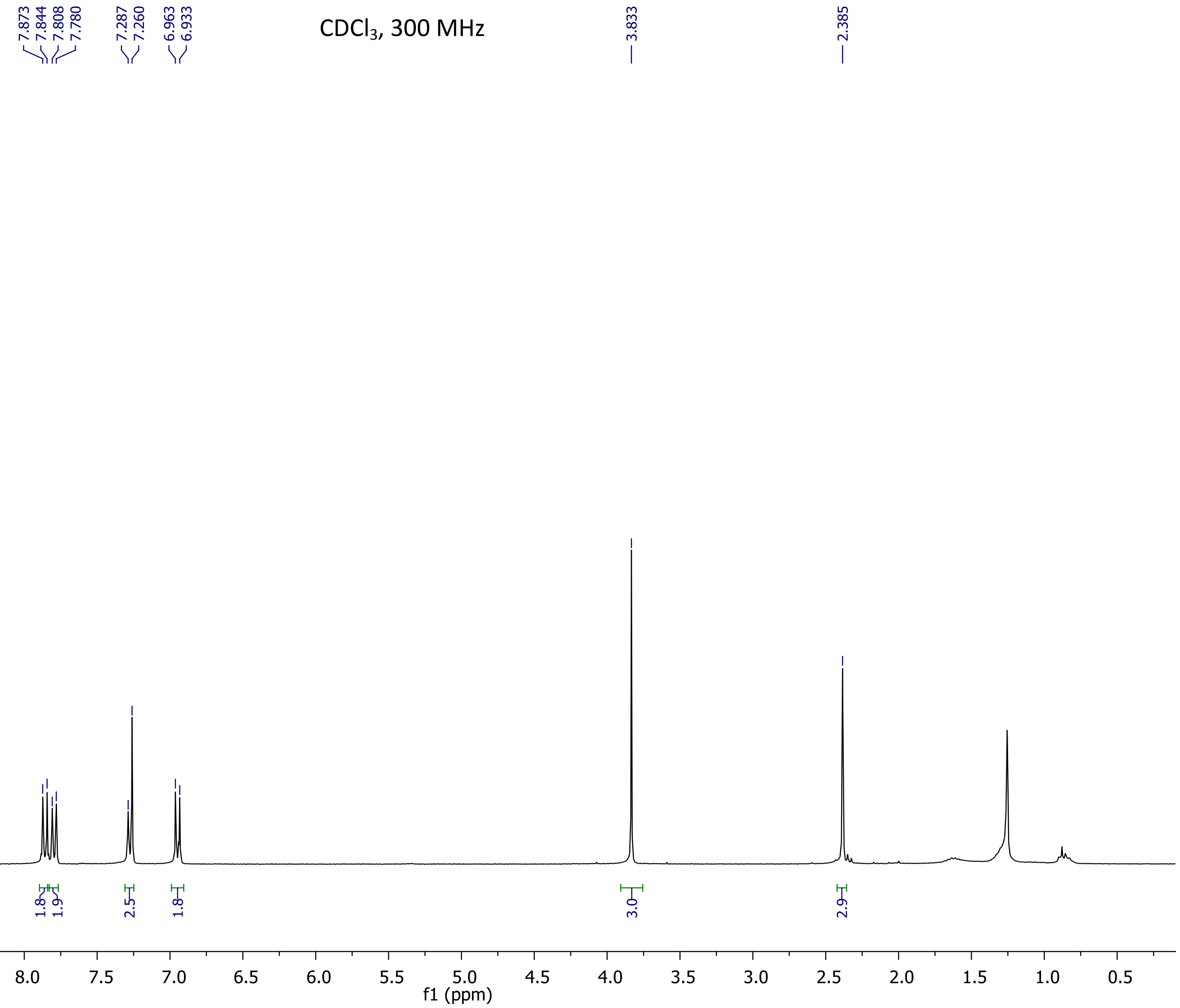

7.873
7.844
7.808
7.780
7.287
7.260
6.963
6.933
CDCl3, 300 MHz
3.833
2.385
1.8
1.9
2.5
1.8
3.0
2.9
10.0 9.5 9.0 8.5 8.0 7.5 7.0 6.5 6.0 5.5 5.0 4.5 4.0 3.5 3.0 2.5 2.0 1.5 1.0 0.5
f1 (ppm)

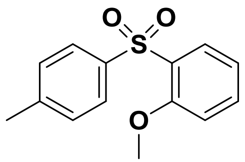

11a

CDCl$_3$, 300 MHz

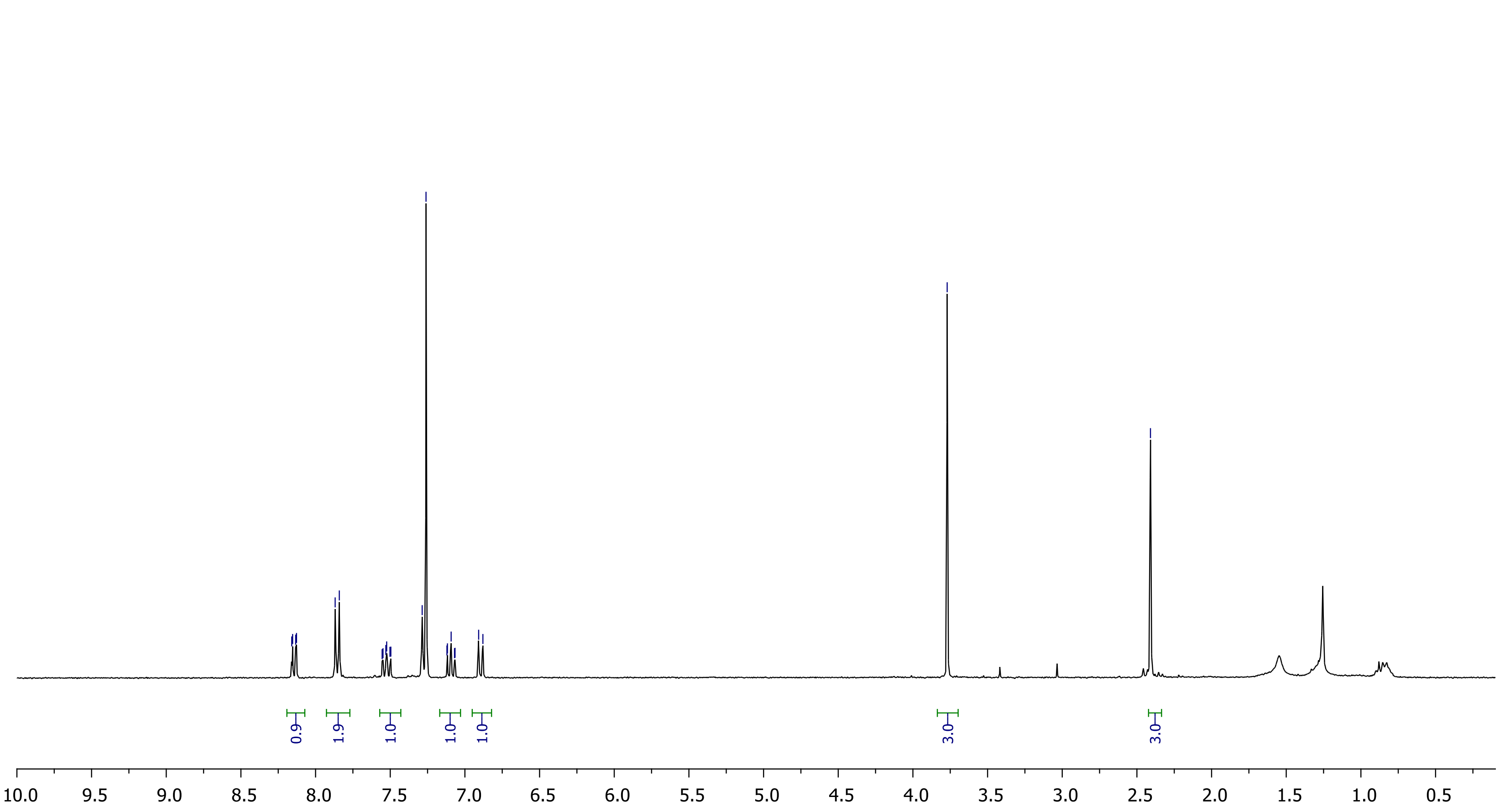

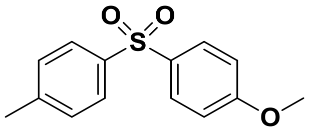

11b

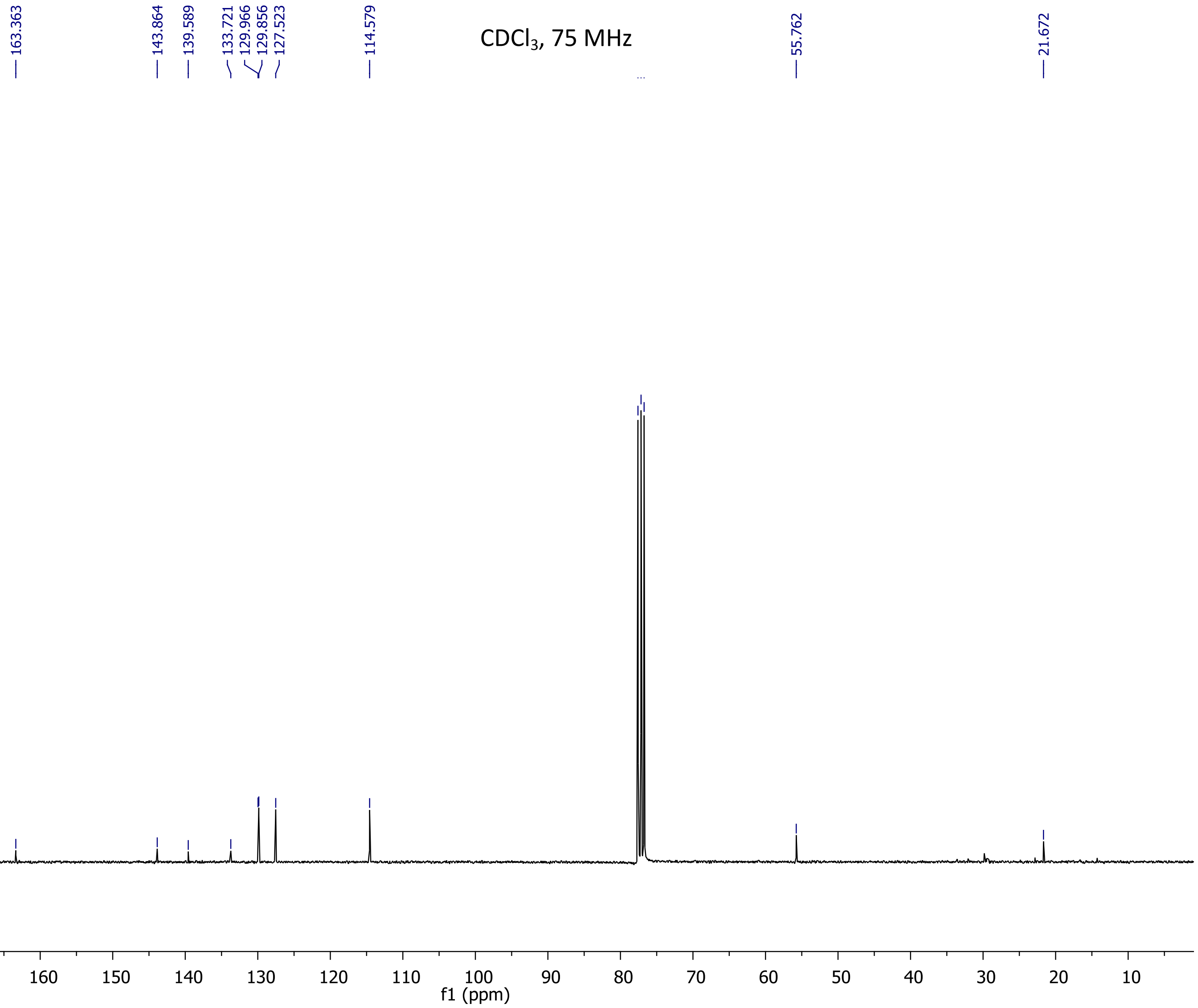

163.363
143.864
139.589
133.721
129.966
129.856
127.523
114.579
$CDCl_3$, 75 MHz
55.762
21.672
200 190 180 170 160 150 140 130 120 110 100 90 80 70 60 50 40 30 20 10
f1 (ppm)

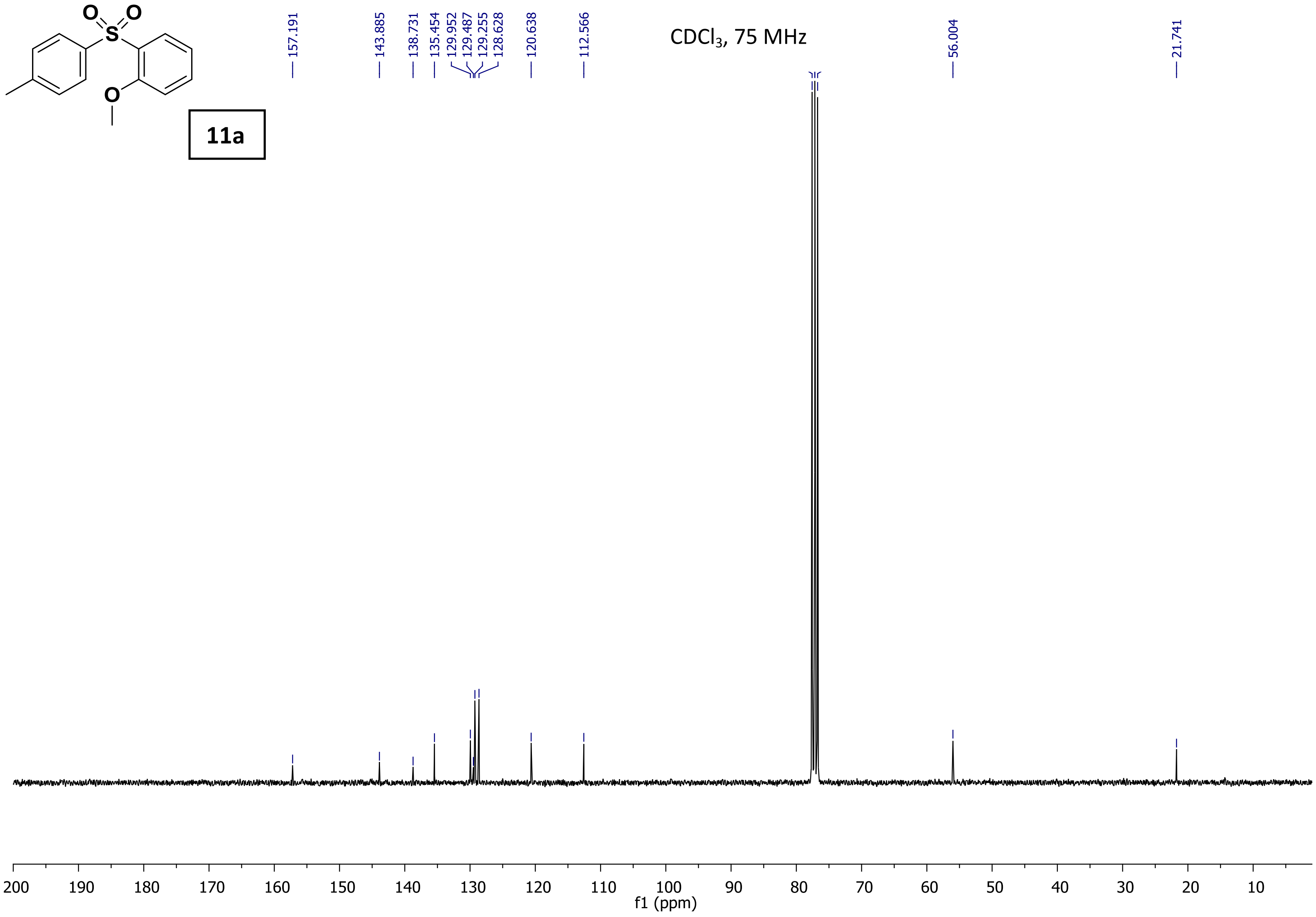

157.191
143.885
138.731
135.454
129.952
129.487
129.255
128.628
120.638
112.566
CDCl$_3$, 75 MHz
56.004
21.741
200 190 180 170 160 150 140 130 120 110 100 90 80 70 60 50 40 30 20 10
f1 (ppm)

11a

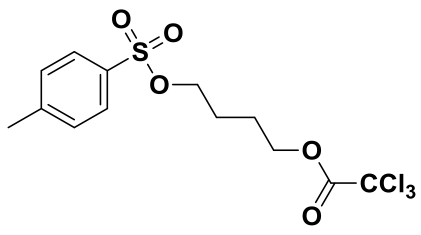

12

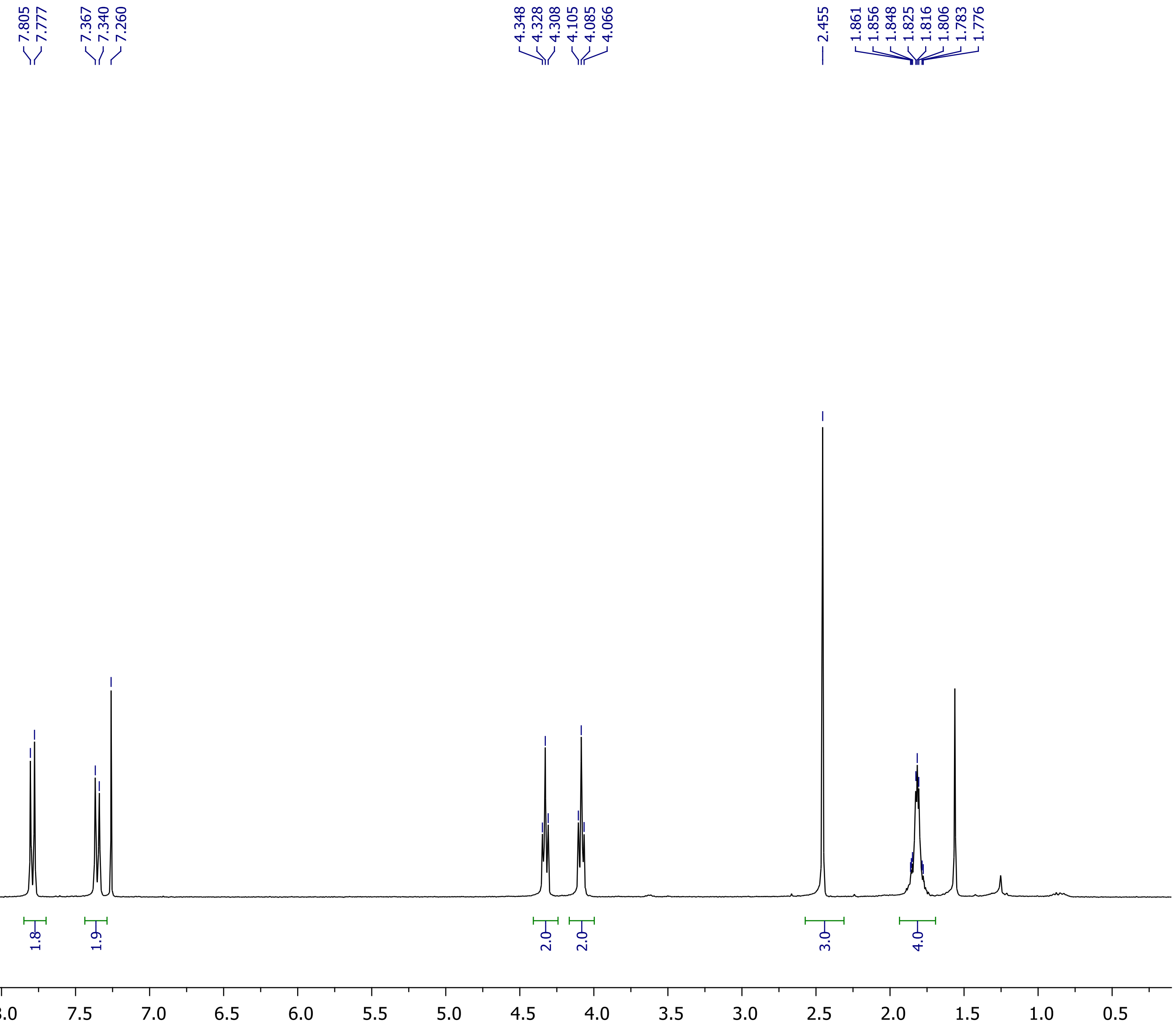

7.805
7.777
7.367
7.340
7.260
4.348
4.328
4.308
4.105
4.085
4.066
2.455
1.861
1.856
1.848
1.825
1.816
1.806
1.783
1.776
1.8
1.9
2.0
2.0
3.0
4.0
10.0 9.5 9.0 8.5 8.0 7.5 7.0 6.5 6.0 5.5 5.0 4.5 4.0 3.5 3.0 2.5 2.0 1.5 1.0 0.5

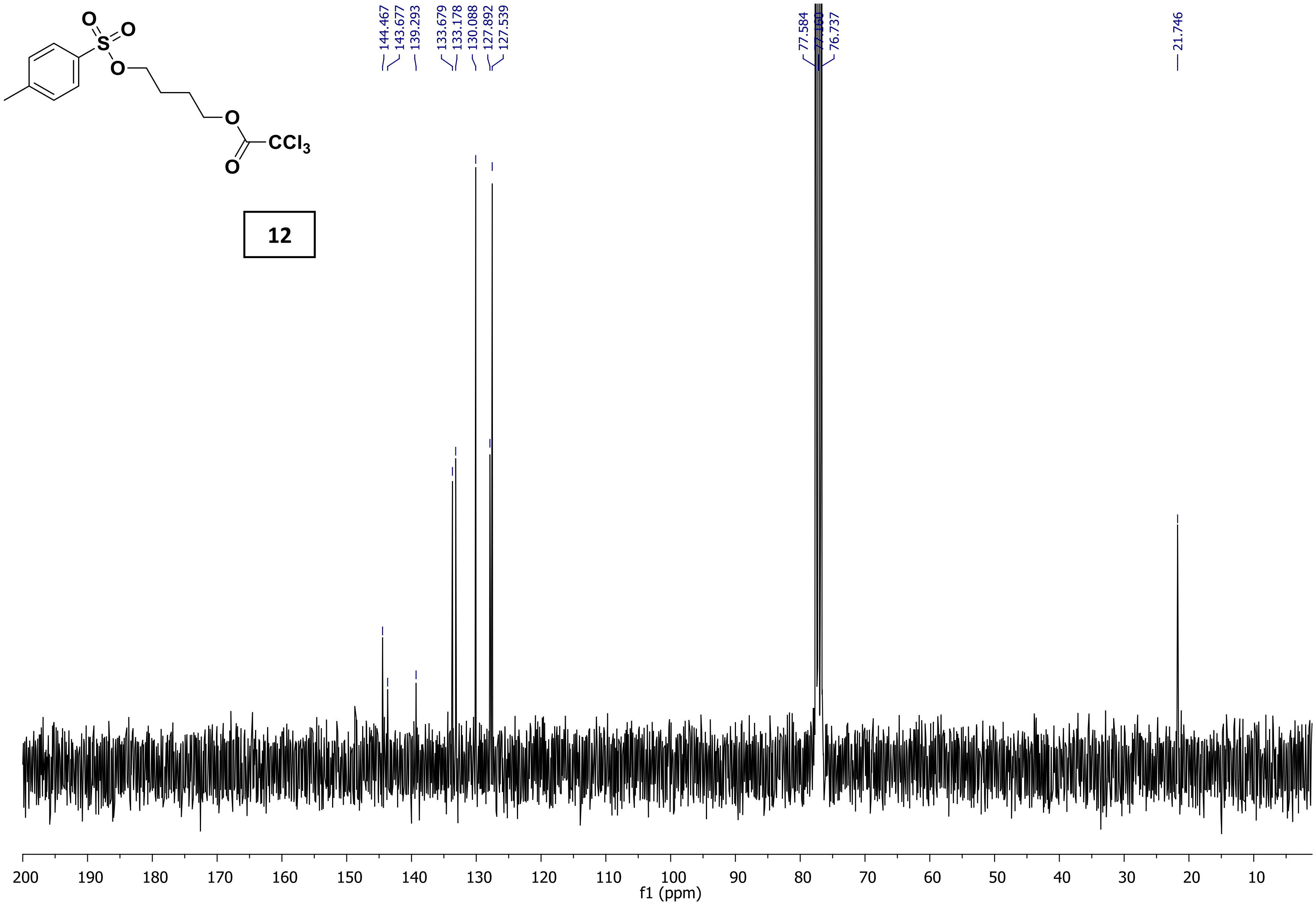
O
S
O
O
O
CCl3
O
144.467
143.677
139.293
133.679
133.178
130.088
127.892
127.539
77.584
77.160
76.737
21.746
200 190 180 170 160 150 140 130 120 110 100 90 80 70 60 50 40 30 20 10
f1 (ppm)

12
